# Mobile Phone Network Data in the COVID-19 era: A systematic review of applications, socioeconomic factors affecting compliance to non-pharmaceutical interventions, privacy implications, and post-pandemic economic recovery strategies

**DOI:** 10.1371/journal.pone.0322520

**Published:** 2025-04-29

**Authors:** Mohammed Okmi, Tan Fong Ang, Muhammad Faiz Mohd Zaki, Chin Soon Ku, Koo Yuen Phan, Irfan Wahyudi, Lip Yee Por

**Affiliations:** 1 Department of Computer System and Technology, Faculty of Computer Science and Information Technology, Universiti Malaya, Kuala Lumpur, Wilayar Persekutuan, Malaysia; 2 Department of Information Technology and Security, Jazan University, Jazan, Saudi Arabia; 3 Department of Computer Science, Universiti Tunku Abdul Rahman, Kampar, Perak, Malaysia; 4 Department of Communications, Faculty of Social and Political Sciences, Universitas Airlangga, Surabaya, Jawa Timur, Indonesia; Fred Hutch Cancer Center: Fred Hutchinson Cancer Center, UNITED STATES OF AMERICA

## Abstract

**Background:**

The use of traditional mobility datasets, such as travel surveys and census data, has significantly impacted various disciplines, including transportation, urban sensing, criminology, and healthcare. However, because these datasets represent only discrete instances of measurement, they miss continuous temporal shifts in human activities, failing to record the majority of human mobility patterns in real-time. Bolstered by the rapid expansion of telecommunication networks and the ubiquitous use of smartphones, mobile phone network data (MPND) played a pivotal role in fighting and controlling the spread of COVID-19.

**Methods:**

We conduct an extensive review of the state-of-the-art and recent advancements in the application of MPND for analyzing the early and post-stages of the COVID-19 pandemic, following Preferred Reporting Items for Systematic Reviews and Meta-Analyses (PRISMA) guidelines. Additionally, we evaluate and assess the included studies using the Mixed Methods Appraisal Tool (MMAT) and the Critical Appraisal Skills Programme (CASP). Furthermore, we apply bibliometric analysis to visualize publication structures, co-authorship networks, and keyword co-occurrence networks.

**Results:**

After the full-text screening process against the inclusion and exclusion criteria, our systematic literature review identified 55 studies that utilized MPND in the context of the COVID-19 pandemic: 46 (83.6%) were quantitative, and 9 (16.4%) were qualitative. These quantitative studies can be classified into five main groups: monitoring and tracking of human mobility patterns (n = 11), investigating the correlation between mobility patterns and the spread of COVID-19 (n = 7), analyzing the recovery of economic activities and travel patterns (n = 5), assessing factors associated with NPI compliance (n = 5), and investigating the impact of COVID-19 lockdowns and non-pharmaceutical interventions (NPI) measures on human behaviors, urban dynamics, and economic activity (n = 18). In addition, our findings indicate that NPI measures had a significant impact on reducing human movement and dynamics. However, demographics, political party affiliation, socioeconomic inequality, and racial inequality had a significant impact on population adherence to NPI measures, which could increase disease spread and delay social and economic recovery.

**Conclusion:**

The usage of MPND for monitoring and tracking human activities and mobility patterns during the COVID-19 pandemic raises privacy implications and ethical concerns. Thus, striking a balance between meeting the ethical requirements and maintaining privacy risks should be further discovered and investigated in the future.

## Introduction

Controlling and fighting infectious diseases often rely on pharmacologic treatments like vaccines and drugs. However, during the COVID-19 pandemic, non-pharmaceutical interventions (NPI) such as lockdowns, mobility restrictions, and social distancing became vital strategies to reduce and slow transmission. Kadi and Khelfaoui [1] found a strong positive correlation between population density and COVID-19 cases, suggesting that monitoring mass population movements and achieving a lower population density can help control its spread. Indeed, human mobility patterns have been linked to the transmission of COVID-19 [2]. As such, tracking these patterns is crucial for disease control and mitigation. Among several tools for capturing mobility patterns, MPND stands out due to its real-time capabilities.

In response to the increasing need for effective tools to monitor and control human mobility during the pandemic, the growth of big data technology and the proliferation of digital data sources have ushered in the era of digital epidemiology. This field leverages sources such as geospatial data, social data, and Mobile Phone Network Data (MPND) to track and analyze human movement and activities, which is crucial for understanding the dynamics of disease spread [3]. Specifically, MPND plays a key role in assisting health authorities and governments in managing and mitigating the spread of COVID-19. It aids in tracking confirmed cases, analyzing travel patterns, and identifying individuals who may have been in close proximity to confirmed cases using mobile tower location data [4]. Additionally, MPND facilitates the identification of high-risk areas and areas with high population density by analyzing mobility inflows and outflows between different locations [5].

During the COVID-19 pandemic, the value of MPND became even more apparent. Studies have found MPND useful in assessing public health interventions and ensuring compliance with NPI [6]. Due to the ubiquity of smartphones and cellular networks, MPND was instrumental in many areas, ranging from predicting infection dynamics [7] to real-time crowd monitoring [8], as well as assessing pandemic impacts on social behaviors.

As MPND’s role expanded during the pandemic, its applications surged, especially in monitoring mobility patterns and enforcing compliance with NPI [9–11]. However, this widespread use of MPND raised ethical and privacy concerns, given the sensitive nature of the data involved [12,13]. In response, measures have been suggested to address these concerns, such as legal frameworks aligned with data protection regulations [14].

Although non-pharmaceutical interventions were crucial in curbing COVID-19 transmission, they had broader socio-economic and health implications. For instance, during lockdowns, there was a spike in mental health drug purchases [15,16], and changes in mobility patterns affected economic activities [17]. Given the pandemic’s recency, much attention has centered on its immediate aftermath. More research is necessary to understand its longer-term impacts [18]. Thus, this research aims to provide innovative solutions and future applications that can be gained from MPND to develop more effective strategies to inform policy decisions and enhance public health interventions in future pandemics.

Specifically, future plans should focus on developing new privacy-preserving methodologies for MPND analysis to ensure public trust and compliance with data protection regulations. As the use of MPND raises privacy and ethical concerns [4], current solutions and recommendations to protect data privacy are not universally applicable. Despite suggestions for setting up legal frameworks and partnerships with mobile operators that comply with the General Data Protection Regulation (GDPR) and establishing data-sharing frameworks for rapid responses to future pandemics [19,20], these temporary solutions are not always applicable in other countries and may only be effective on a local basis. Hence, there is a need for enhanced privacy-preserving techniques that ensure data security while maximizing utility, along with secure data-sharing protocols. These steps can include agreements and collaborations between mobile network operators, public health authorities, and researchers to enhance the use of MPND in future public health interventions.

In addition to privacy concerns, future plans should also include post-pandemic recovery planning. The use of MPND in monitoring the recovery of economic activities and tourism post-pandemic has been reported [21]. However, other approaches for post-pandemic recovery planning should be explored, such as informing urban planning decisions by identifying changes in population movement patterns and density, improving future disaster preparedness and response, and assisting in developing targeted tourism recovery strategies by analyzing changes in visitor behavior.

Hence, this systematic literature review (SLR) delves into the latest findings related to MPND applications in the context of the COVID-19 pandemic while discussing the potential opportunities and challenges that mobile phone companies, decision-makers, governments, and public health experts should consider when ethically and effectively utilizing MPND. Despite prior research [5,6,19], there remains a lack of comprehensive reviews discussing the current state of this field. Specifically, the multiple practical applications and processing techniques built by utilizing mobile phone network data [22] to manage and control the spread of the virus through multiple waves of the COVID-19 epidemic and the adoption of the data in the post-COVID era to monitor economic recovery activities and provide real-time insights into business activities led to the need for a systematic literature review to fill this gap. Hence, our article aims to fill this gap, focusing on:

- Analyzing MPND’s Role Across Different COVID-19 Waves: This theme examines how MPND tracked mobility changes during various pandemic stages, correlating these changes with virus spread. It highlights the role of MPND in providing foundational insights into the effectiveness of initial NPI measures, setting the stage for broader applications and impacts.
- Diverse Applications of MPND in Public Health: Building on the first theme, this section explores the applications of MPND in managing public health measures. This includes evaluating the effectiveness of NPI policies such as social distancing, enhancing public health strategies, and adapting policies in real-time using mobility data feedback.
- Socioeconomic and Cultural Impacts on NPI Adherence: This theme extends the discussion by examining how NPIs influence broader human behavior and societal norms. MPND, enriched with demographic details like age, gender, and location, reveals patterns of adherence to NPIs among different socio-economic and cultural groups. It identifies demographics most affected by restrictions, such as lockdowns, and highlights challenges faced by specific groups in accessing essential services or adhering to public health guidelines.
- MPND’s Utility in Economic and Tourism Recovery Post-Pandemic: This theme shifts focus to post-pandemic recovery efforts, showing how MPND informs strategies for economic and tourism recovery. It illustrates how mobility data can be leveraged for long-term planning and rebuilding efforts.
- Privacy and Ethical Concerns with MPND Usage: The final theme addresses privacy and ethical challenges associated with sensitive mobility data. It underscores the need for robust privacy protections and ethical frameworks to ensure that the benefits of MPND are balanced against safeguarding individual privacy rights.

The paper unfolds as follows: The Materials and Methods section outlines the research methodology. The Results section presents search results, study features, research question responses, and our taxonomy. The Discussion and Future Directions section discusses study implications, findings, and future research. The Conclusions and Limitations section concludes the review.

## Materials and methods

This section details the study methodology used to conduct the systematic literature review (SLR). For a comprehensive understanding of the recent advancements and methodologies related to the use of MPND in informing COVID-19 responses, we followed the guidelines in S1 Table presented in the Preferred Reporting Items for Systematic Reviews and Meta-Analyses (PRISMA) [23]. PRISMA’s primary objective is to ensure detailed documentation of study methods, results, and limitations, thereby enhancing the reliability and validity of scientific findings. Specifically, PRISMA was instrumental in refining the scope of this review by systematically filtering studies that explicitly utilized MPND while excluding those relying solely on other mobility datasets, such as app-based GPS, travel surveys, or smart card data. Given the diverse methodologies in mobility research, PRISMA provided a structured selection process that ensured the inclusion of only relevant studies related to MPND applications. Additionally, this framework enhanced reproducibility, making it easier for future research to adopt similar approaches for systematic reviews on MPND-based mobility analysis.

To ensure a comprehensive and up-to-date evaluation of the literature on the use of MPND in controlling the spread of COVID-19, improving public health responses, tracking population movements, assessing the effectiveness of NPI, and evaluating the recovery of economic activities, this systematic review was conducted using multiple authoritative databases, including Scopus, Web of Science, and PubMed. The review covers MPND studies published between 2020 and 2023, with the most recent research conducted on 5 November 2023 to ensure the inclusion of the latest available research.

Throughout the pandemic, MPND played a pivotal role in assisting public health institutions and governments in combating COVID-19. This assistance took the form of monitoring and analyzing changes in individuals’ mobility patterns and investigating the correlation between these patterns and the daily incidence of COVID-19 cases. In the aftermath of the pandemic, or in the post-COVID-19 phase, the data has been crucial in analyzing and assessing economic activities and tourism trends. Notably, a myriad of applications has been developed based on MPND to analyze both the early stages and the aftermath of the COVID-19 pandemic. Therefore, this SLR seeks to identify studies related to this subject matter. The research questions guiding this review are: “What is the current state of applications and approaches that utilize mobile phone network data for addressing and managing the coronavirus?”; “What are the ethical implications and privacy concerns of using mobile phone network data in response to the COVID-19 pandemic?”; and “What role does mobile phone network data play in the post-COVID-19 era?”.

### Search strategy and information sources

#### Information Sources.

We conducted a search for pertinent research articles and conference papers using three primary databases: Scopus, Web of Science, and PubMed. The details of this search are presented in Table 1.

**Table 1:** Details of the search fields and filters for each database.

Database	Timespan	Content Type
Scopus	2020-2023	“Article, Review article, Conference paper”
PubMed	2020-2023	“Article, Review article”
Web of Science	2020-2023	“Article, Review article”

#### Search terms.

Two principal terms, “mobile phone network data” and “COVID-19”, were singled out as crucial for pinpointing relevant studies. To ensure comprehensive retrieval of all pertinent research, we expanded our search to include variations of the term “mobile phone data”. The specific keyword search employed in this study was: (“mobile phone network data” OR “mobile phone data” OR “mobile phone datasets” OR “call detail records” OR “call data records”) AND (“COVID-19” OR “SARS-CoV-2” OR “COVID” OR “2019-nCoV” OR “corona”).

#### Study eligibility criteria.

The studies were selected based on the inclusion (IC) and exclusion (EC) criteria detailed in Table 2.

**Table 2:** Eligibility criteria for the study.

IC	EC
1. Studies exclusively address the use of MPND generated by mobile network operators in response to the COVID-19 pandemic.	1. Studies utilizing mobile phone network data not sourced from mobile network providers, such as data from phone applications, GPS, or other app-based platforms.
2. Studies that offer scientific contributions to the use of MPND for controlling and monitoring COVID-19.	2. Studies exclusively focus on the use of mobile phone applications to control the COVID-19 outbreak.
3. Studies are written in the English language.	3. Studies are not written in the English language.
4. Studies were published between the years 2020 and 2023.	4. Studies published prior to 2020.

This study applied four inclusion criteria to ensure that the selected research aligned with the study’s objective of examining MPND applications during the COVID-19 pandemic. The criteria were designed to focus on studies that specifically utilized MPND generated by mobile network operators, allowing for a precise evaluation of its role in mobility analysis, public health responses, and economic recovery. The temporal criterion, restricting the selection to studies published between 2020 and 2023, was implemented to cover all phases of the COVID-19 pandemic, ensuring that the findings reflect MPND’s evolving applications from the outbreak to post-pandemic recovery. The decision to include only English-language publications was necessary to maintain interpretational accuracy and methodological consistency, as multilingual data synthesis introduces challenges related to terminological differences and potential misinterpretation in translated content.

Conversely, the exclusion criteria were structured to remove studies that did not meet the methodological focus of this review. Research that relied on non-MPND mobility datasets, such as GPS, smart card data, or mobile applications, was excluded due to fundamental differences in data collection methods, spatial resolution, and analytical frameworks. Studies published before 2020 were also excluded, as they predate the pandemic and do not contribute to understanding MPND’s role in COVID-19 mobility analysis. By defining clear selection parameters, this review ensures that the included studies provide a coherent and analytically comparable dataset for evaluating MPND’s effectiveness in pandemic response efforts.

A detailed summary of the excluded studies, including their titles, types of data used, and specific reasons for exclusion, is provided in S3 Table.

### Study selection process and quality assessment

#### Selection process.

The study selection process consisted of four phases, which were carefully conducted and evaluated by a team of four researchers. Two authors (M.O. and L.Y.P.) conducted the full-text screening, while the other two authors (T.F.A. and C.S.K.) assessed the selected studies for potential bias. At the identification phase, 267 studies were initially identified after we applied the search terms to the title, abstract, and keyword filters. Table 1 displays the configuration of the search query in the selected databases. The data retrieved from these databases was imported into a Microsoft Excel spreadsheet and subsequently organized by relevance for the next phase. In the screening phase, which encompassed two steps—identifying duplicate studies and manually screening titles and abstracts for relevancy—135 studies were discarded due to duplication, and an additional 12 were removed after title and abstract reviews. During the eligibility phase, characterized by a thorough full-text review against the inclusion and exclusion criteria, 120 studies were shortlisted for detailed examination. Studies leveraging phone application data or other app-based mobile data were excluded during this phase. Consequently, after the comprehensive text review, a total of 55 studies remained. The list of included and excluded studies, along with reasons for exclusion, is detailed in S4 Table.

#### Quality assessment.

A thorough quality assessment was conducted to evaluate the selected papers, aiming to systematically examine the strengths and limitations of both quantitative and qualitative studies in terms of their research design, sampling strategies, and analytical rigor. Given the complexities associated with MPND research, the authors (T.F.A., M.F.M.Z., and L.Y.P.) utilized established quality appraisal frameworks to ensure a robust evaluation of study methodologies.

For quantitative studies, the Mixed Methods Appraisal Tool (MMAT) [24] was chosen due to its structured approach in assessing methodological quality, particularly in studies employing non-traditional data sources such as MPND. The MMAT is specifically designed to evaluate studies using complex, heterogeneous data sources, making it highly suitable for mobility research where data quality varies based on network infrastructure, population coverage, and data processing techniques. This tool allowed for a systematic assessment of five key quality criteria that are particularly relevant to MPND-based mobility research:

1. Relevance of the sampling strategy: Given that MPND is collected through passive and active methods across cellular network infrastructure, including cell towers, base stations, and switching centers, sampling strategies were assessed for their ability to capture diverse geographic regions and demographic groups. This helped mitigate biases arising from the over-representation of urban areas or specific socio-economic segments.
2. Sample representativeness: MPND data is influenced by variations in network coverage and mobile phone usage patterns. Studies were evaluated on how well they accounted for these disparities, ensuring balanced representation across urban and rural populations.
3. Appropriateness of measurements: Studies were examined to determine whether the temporal and spatial granularity of MPND was sufficient to capture meaningful mobility patterns, particularly in areas with varying cell tower densities.
4. Risk of nonresponse bias: Since MPND does not uniformly cover all population segments (e.g., regions with lower mobile phone penetration), studies were assessed on whether they addressed potential data gaps through weighting techniques or integration with supplementary datasets.
5. Suitability of statistical analysis: Given the complexity of MPND, studies were reviewed for their use of rigorous statistical methods, including data cleaning techniques, aggregation strategies, spatial clustering algorithms, and mobility pattern comparison across pandemic phases.

For qualitative studies, the Critical Appraisal Skills Programme (CASP) checklists were applied to ensure methodological rigor in studies focusing on privacy concerns, ethical considerations, and public perception of MPND applications. The CASP framework was selected because it provides a structured approach for evaluating the trustworthiness and transparency of qualitative research, which is crucial when analyzing subjective aspects of MPND, such as ethical implications and societal concerns. The assessment covered:

1. Clarity of research aims: Studies were reviewed for well-defined objectives, particularly those analyzing human mobility shifts in response to pandemic-related interventions.
2. Rigor in research conduct: The methodological soundness of qualitative analyses was assessed, focusing on the depth of mobility pattern interpretation and robustness of evidence supporting the findings.
3. Ethical considerations: Given the sensitive nature of mobility data, studies were evaluated on how they balanced public health benefits with individual privacy protections, including the implementation of anonymization techniques and adherence to GDPR regulations.
4. Transparency in reporting: Research transparency was assessed by reviewing data processing workflows, potential biases, and limitations affecting result generalizability.
5. Clarity in presentation of findings: Studies were examined to ensure that conclusions were well-supported by the data, findings aligned with stated research objectives, and discussions on privacy risks were explicitly addressed.

### Data extraction and analysis

#### Data extraction.

The development of a data extraction methodology is a pivotal aspect of this study. It aids in probing the research questions, framing significant findings, and pinpointing recurring patterns and themes in studies related to mobile phone network data. Table 3 showcases the data items that this study intends to extract from the chosen papers. These items encompass the characteristics, features, and attributes of mobile phone data and the nations participating in the co-authorship network related to mobile phone data.

**Table 3:** List of data items.

Data items	Data description	Analysis types
Bibliographic information	Research title, year of publication, author name, and other relevant information were extracted from publishers’ websites and academic databases	Descriptive analysis
Adherence measures and factors influencing compliance with the NPI	The data was obtained from the literature	Descriptive analysis
MPND features and characteristics	The data was obtained from the literature	Content analysis and narrative synthesis
List of keywords and the total number of times the keyword appears across all publications	The data was gathered from the Scopus database	Mapping and bibliometric analysis
List of countries involved in the co-authorship network of the MPND	The data was extracted from the Scopus database	Mapping and bibliometric analysis

#### Data synthesis and analysis.

The data synthesis process aimed to analyze and summarize the findings from the selected papers and to present the data from these studies through charts, tables, network visualizations, and tree diagrams. This synthesized data forms the main body of evidence used to address the research questions, discuss the limitations of current studies, and draw conclusions on the topic.

Furthermore, this study incorporates bibliometric analysis to offer deeper insights into patterns, trends, and variances across mobile phone data studies. Bibliometric analysis is a statistical method that evaluates bibliographic data to visualize, assess, and quantify scientific outputs within a specific scientific field. This approach offers a comprehensive view of patterns and trends in studies within a particular domain, enhancing the overall scientific documentation. Notable quantitative bibliometric techniques include co-occurring keyword analysis, which depicts a network based on the frequency of keywords in MPND studies, and co-authorship analysis, which showcases collaboration patterns among authors and countries. The VOSviewer program is utilized to visually represent the co-occurrence of keywords in studies related to COVID-19 and mobile phone network data.

While this study incorporates various analytical approaches, including descriptive analysis, content analysis, mapping, and bibliometric analysis, it is essential to note that certain statistical techniques, such as measures of effect and meta-regression, were excluded from the scope of our study. The rationale behind this decision is rooted in the primary focus of our research, which centers on the current state of applications and approaches that utilize mobile phone network data for fighting and managing the coronavirus. Additionally, during the pandemic, tremendous growth in novel applications fueled by mobile phone network data emerged as part of efforts to control the COVID-19 pandemic. This growth raised ethical concerns and privacy issues owing to the confidential nature of the data it encompasses. Therefore, the present review is carefully crafted to provide an in-depth discussion of these concerns, among its primary objectives.

## Results

This section presents the results of the study selection process, charts the volume of MPND publications over time, addresses the research questions, introduces a taxonomy of MPND studies, and discusses findings related to ethical implications and privacy concerns arising from MPND usage.

### Study selection, publication trends, and quality assessment

#### Search results.

**Fig 1:**
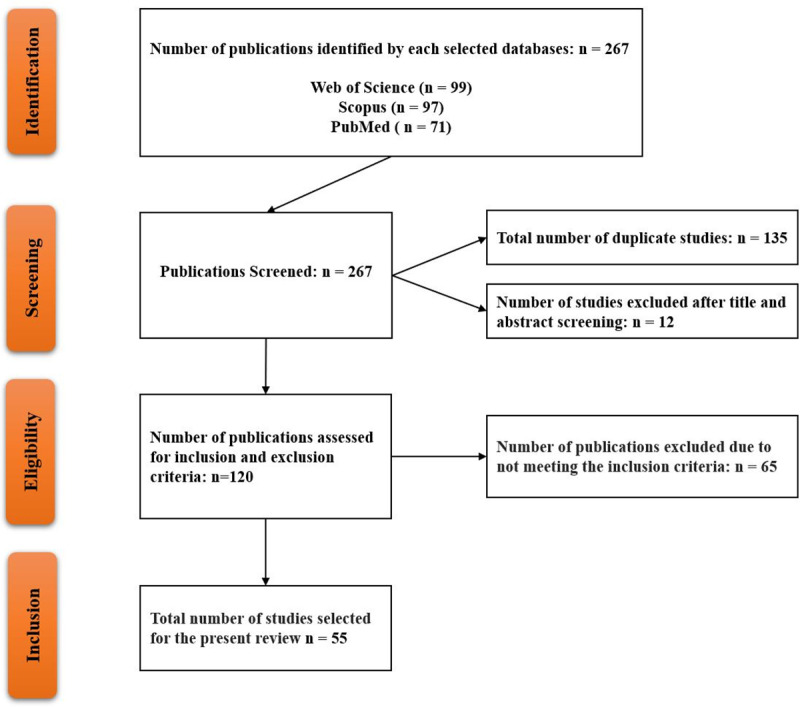
The PRISMA flowchart consists of four main stages: identification, screening, eligibility, and inclusion, used for selecting studies on the use of MPND in COVID-19 responses.

A comprehensive search of the selected databases resulted in 267 studies. Of these, 55 studies were selected to offer a current overview of research on the use of MPND in response to COVID-19. The selection process for these 55 primary studies, including screening titles and abstracts and conducting a full-text review for eligibility, is depicted in Fig 1.

#### Publication type.

Among the 55 primary studies analyzed, 53 were published in peer-reviewed journals and 2 in conference proceedings. The dominance of journal articles reflects the research community’s commitment to advancing well-substantiated studies that address complex issues such as epidemiological tracking, mobility pattern analysis, and the socioeconomic impacts of the pandemic. This trend highlights the maturity and depth of the research, which builds upon established knowledge bases and offers comprehensive insights into public health responses. In contrast, the limited number of conference presentations suggests their role as forums for the initial exploration of new methodologies and early-stage findings in monitoring the spread of the virus.

#### Publication over time.

**Fig 2:**
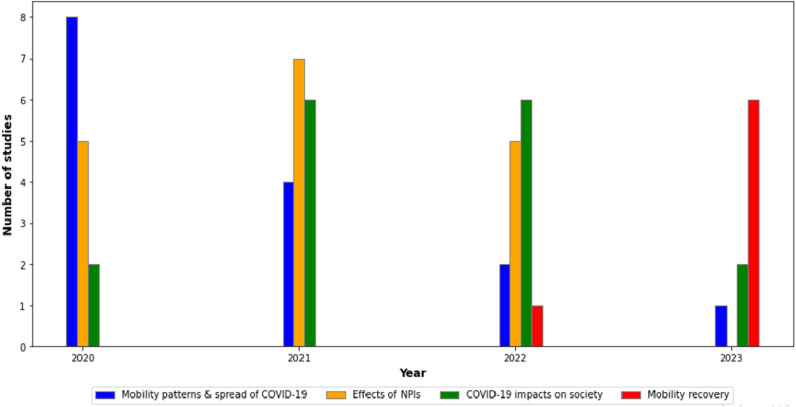
Temporal distribution of studies utilizing MPND from 2020 to 2023, categorized into four research focuses: mobility patterns and spread of COVID-19 (blue bars), effects of NPIs (orange bars), COVID-19 impacts on society (green bars), and mobility recovery (red bars).

Fig 2 provides a visual summary of the temporal distribution of studies concerning MPND research from 2020 through November 2023. A clear surge in research activity is visible between 2020 and 2022, aligning with the peak periods of the COVID-19 pandemic.

In the early stages of the pandemic in 2020, there was a notable concentration of studies focusing on “mobility patterns and spread of COVID-19,” as depicted by the blue bar, which underscores the urgency of understanding the virus’s propagation through human movement. During this period, studies extensively analyzed the association between mobility patterns and COVID-19 spread, utilizing MPND to track and monitor changes in travel and mobility behaviors.

The focus in 2021 and 2022 shifted more toward the “Effects of NPI,” as reflected by the orange bars. Research prioritized evaluating the impact of NPI, such as travel bans and stay-at-home orders, on controlling infection rates and curtailing the virus’s spread.

Concurrently, there was a growing body of research examining “COVID-19 impacts on society,” as the green bars reveal. These studies delved into the broader socio-economic implications of the pandemic, exploring the consequences on urban dynamics, city life, and the overall economy. They evaluated how various interventions have affected urban life, reducing travel and foot traffic, hurting the economy with job losses and company closures, and changing education to remote learning.

As we approach the end of 2022 and the beginning of 2023, the research lens shifts towards “mobility recovery” at the end of 2022 and the beginning of 2023, as indicated by the red bars. This emerging interest focuses on understanding the post-pandemic recovery, specifically examining the resilience and adaptability of societal mobility in the wake of lifted restrictions and returning to normalcy.

#### Quality of included studies.

Out of the 46 quantitative studies analyzed, 31 studies (67.39%) demonstrated high quality with MMAT scores of 100%, indicating complete adherence to the criteria. Fifteen studies (32.61%) demonstrated medium quality, with MMAT scores ranging from 60% to 80%. This indicates that these studies partially adhered to the criteria, as they did not meet all five criteria. There were no quantitative studies classified as low-quality.

Notably, certain quantitative studies fell short of fulfilling criterion 1.2 (sample representativeness) due to the presence of specific limitations. A recurring concern was the limited inclusion of specific groups in the sample, which may result in a lack of full representativeness of the entire target population. For instance, in the studies conducted by [25] and [26], the absence of demographic information or the lack of socio-demographic profiling hinders the ability to determine if the users are a representative sample of the entire population. This raises questions over the representativeness of these samples. In certain instances, like the study conducted by Lai [27], the traveler sample was limited to smartphone users of a specific app. This may lead to an inadequate and biased representation of travelers. Furthermore, the accuracy and reliability of mobile phone usage data recorded in rural areas have raised concerns about the representativeness of the sample due to the scarcity of cell towers outside urban areas. This scarcity may affect the representativeness and completeness of the data collected in non-urban regions. The quality appraisal of the quantitative articles is described in S2 Table.

Within the pool of qualitative research (n = 9), our analysis revealed that 4 of these studies have successfully satisfied the rigorous standards for high quality since they satisfy all of the criteria outlined by the CASP. The medium-quality studies exhibited weaknesses in terms of rigor, ethical considerations, and their effectiveness in presenting significant findings. Details of the CASP assessment for the qualitative studies are reported in S2 Table.

#### Study characteristics.

Table 4 presents the predominant characteristics observed in quantitative studies examining the impacts of NPI across various stages of the COVID-19 pandemic. These studies illustrate how the effectiveness of interventions such as lockdowns, travel restrictions, and social distancing varies depending on their timing, demographic focus, and type of intervention.

**Table 4:** Main characteristics of the quantitative studies included in the systematic review.

Reference	Study theme	Demographic	Country	Covid phase or stage	NPI measure	MPND features/mobility metrics
[2]	To monitor mobility patterns and their relationship with COVID-19 transmission	Residents, travelers	Spain	Pre-pandemic, lockdown, post-lockdown	Travel restrictions, social distancing	Origin-destination matrices
[3]	To observe variations in human movement patterns	General population	Hungary	First wave of COVID-19 (February-May 2020)	Social distancing, school closures	Relative mobility index
[7]	To examine the correlation between population outflow and the transmission and expansion of COVID-19	Travelers	China	Early outbreak (January– February 2020)	Travel restrictions	Mobility outflow index
[8]	To investigate the relationship between movement patterns and the occurrence of COVID-19 cases	Residents, visitors	Spain	First wave of COVID-19 (March 2020)	Lockdowns, travel restrictions	Mobility flow Index, hotspot risk index
[9]	To study the relationship between socioeconomic factors and people’s responses to the mobility restrictions implemented by the Italian government	Youth (ages 16–29), Adults (ages 30–49), Elderly (ages 65+)	Italy	Pre-pandemic, lockdown	Mobility restrictions, stay-at-home orders	Radius of gyration, average spatial range of users’ movements
[12]	To quantify the number of trips and daily entrances and examine their correlation with the increase in COVID-19 cases	Workers, residents, travelers	Andorra	Partial confinement (13 March), total confinement (March 18), full reopening (June 1)	Mobility restrictions, Stay-at-home orders, border restrictions	Total daily trips
[13]	To evaluate the effectiveness of mobility restrictions on curbing the spread of COVID-19 in China	Individuals within the age range of 15–65 years, residents	China	Pre-intervention phase (January 10–February 6, 2020), intervention (February 7 onward)	Mobility restrictions	Origin-destination matrices
[15]	To explore how the physical features of urban parks can affect visitation patterns	Visitors	South Korea	Pre-pandemic (Feb 1, 2019–Jan 31, 2020), Pandemic (Feb 1, 2020–Jan 31, 2021)	Social distancing, mobility restrictions	Park visit frequency
[16]	To examine the negative effects of lockdown and social distancing measures on mental health	Patients	Italy	Lockdown periods	Social distancing mobility restrictions	Total daily trips
[21]	To examine changes in tourist mobility patterns	Tourists: minors (under 18), adults (19–39), elderly (50 and above), gender groups (male, female)	China	Two COVID-19 waves: First wave (Jan–Feb 2020), Second wave (Jan–Feb 2021)	Travel restrictions, mobility restrictions	Daily inter-city tourist movements (inflow/outflow)
[25]	To explore the impact of COVID-19 lockdowns on human behavior	Residents, commuters	Italy	Pre-lockdown, lockdown (March–April 2020), post-lockdown (July–September 2020)	Mobility restrictions	Relative change in population size
[27]	To analyze travel patterns during the initial phase of the pandemic by estimating the total volume of population flow and inflow between Chinese cities	Travelers, air passengers, college students	China	Early pandemic phase (January–April 2020).	Travel restrictions	Origin-destination matrices
[28]	To explore the impact of mobility restriction measures on reducing the transmission of COVID-19	Workers, residents	United States of America	Early stages of the epidemic	Social distancing, stay-at-home orders	Total daily trips, origin-destination matrices
[29]	To analyze the impact of COVID-19 on human activities and travel behaviors	Workers, travelers, residents	China	Pre-pandemic, during the outbreak (January 2020–March 2020), after COVID-19 was under control (April 2020–August 2020)	Travel restrictions, stay-at-home orders	Number of active days, home-work distance
[30]	To explore the effects of COVID-19 on the changes in travel behaviors	Travelers, residents, low-income individuals, high-income individuals	China	Pre-pandemic, COVID peak phase, recovery phase	Travel restrictions, stay-at-home orders, closure of public venues	Travel frequency
[31]	To analyze the mobility patterns of Chinese workers	Rural migrant workers, youth (ages 16–29), adults (ages 30–49), seniors (ages 50–65)	China	Post-COVID-19 era	Social distancing, travel restrictions	Origin-destination matrices
[32]	To analyze the factors that impact changes in intracity mobility patterns	Travelers aged 20–60 years and above, low-income individuals, high-income individuals	China, Worldwide	Early pandemic phase	Stay-at-home orders, mobility restrictions	Proportion of people traveling within cities, Population flow intensity
[33]	To examine the changes in human movement patterns resulting from lockdown measures	Residents and non-residents aged 18–64 and those aged 65 and above	France	Pre-lockdown, lockdown period	Travel restrictions, school closures	Origin-destination matrices
[34]	To investigate the impact of political beliefs on adherence to stay-at-home and social distancing orders	Residents in U.S. counties categorized by political alignment (Republican vs. Democratic)	United States of America	Pre-lockdown (January 1–March 18, 2020), lockdown phase (March 19–April 23, 2020)	Stay-at-home orders, social distancing	Proportion of individuals confined to their residences
[35]	To examine the correlation between political factors and adherence to NPI measures	Republicans, workers, low-income individuals, high-income individuals	United States of America	Pre-lockdown (January–March 2020), NPI mandate phase (March–April 2020)	Travel restrictions, stay-at-home orders	Daily percentage change in mobility
[36]	To evaluate the effects of lockdown measures on mobility patterns	Travelers, tourists	Austria	Pre-lockdown, lockdown	Travel restrictions, stay-at-home orders	Radius of gyration, origin-destination matrices
[37]	To monitor population flows and changes in human mobility patterns	Travelers	China	Lockdown, recovery	Travel restrictions, stay-at-home orders	Radius of gyration, origin-destination matrices
[38]	To evaluate the effects of lockdowns on the reduction of COVID-19 transmission	Travelers, urban and rural resident	Ghana	Early stages of the COVID-19 outbreak (March–May 2020), lockdown (April 2020)	Travel restrictions, social distancing	Origin-destination matrices, normalized movement index
[39]	To detect regions with a high risk of COVID-19 transmission and analyze the movement patterns of individuals belonging to high-risk groups	Residents, travelers	China	Early stage of the pandemic (January–February 2020)	Travel restrictions	Origin-destination matrices, network centrality metric
[40]	To examine the variations in demographic and movement patterns	Workers, residents	Spain	Lockdown phase (April 2020), recovery phase (August 2020	Travel restrictions	Origin-destination matrices
[41]	To investigate how social connections influence compliance with mobility restrictions	Counties categorized by education levels and partisan alignment	United States of America	Early pandemic (Feb–Mar 2020)	Social distancing measures	Social distancing metric
[42]	To examine the structural changes in intercity mobility networks	Travelers	China	Pre-lockdown, lockdown, post-lockdown.	Travel restrictions	Intercity mobility flow matrix
[43]	To examine urban-to-rural mobility and its relationship with multi-local living to enhance crisis preparedness	Residents	Finland	First wave of COVID-19 (March–May 2020)	Travel restrictions	Origin-destination matrices
[44]	To detect mass gatherings and support the allocation of resources for COVID-19 response in a low-resource setting	Residents	Malawi	Lockdown	Social distancing measures	Number of active subscribers at cell towers
[45]	To propose a privacy-preserved and cost-efficient control scheme for COVID-19	Suspected COVID-19 individuals	Pakistan	During the pandemic (2020–2021)	Quarantine measures	Call history of a patient
[46]	To monitor COVID-19 patients	Suspected COVID-19 individuals	Pakistan	During the pandemic (2020–2021)	Quarantine measures	Call duration, call type
[47]	To integrate genomic data with MPND to map the geographical distribution and spatial spread of SARS-CoV-2 variants	General population	Bangladesh	First wave (March–July 2020)	Stay-at-home orders	Travel frequency, average distance traveled
[48]	To examine the relationship between human mobility and COVID-19 infections	General population	Japan	First wave of COVID-19	Mobility restrictions	Origin-destination matrices
[49]	To track mobility patterns during COVID-19 to inform policy responses	General population	The Gambia	First wave of COVID-19 (March–May 2020)	Mobility restrictions	Mobility inflow index
[50]	To study the impact of COVID-19 lockdown measures on urban dynamics and land use in	General population	Spain	First wave of COVID-19 (February–May 2020)	Mobility restrictions	Origin-destination matrices
[51]	To explore the effects of the COVID-19 pandemic on daily human activities	General population	Sweden	First wave of COVID-19 (January–March 2020)	Mobility restrictions	Standard Deviation metric
[52]	To explore visitation behavior to US national parks during the COVID-19 pandemic, focusing on racial disparities in access and visitation	Non-white communities, African-American, Hispanic, Asian-American, Native American	United States of America	First wave of COVID-19 (March 2020 onward)	Social distancing, travel restrictions	Visitation counts normalized by census block population; distance decay effect on visitations based on proximity to national parks
[53]	To investigate the impact of COVID-19 lockdown measures on human activities	General population	Japan	First wave of COVID-19 (January–May 2020)	Non-compulsory measures including requests for voluntary self-restriction	Aggregated mobility metric
[54]	To examine the impact of COVID-19 on regional socio-economic activity and behavioral dynamics	Municipal population	Latvia	Different COVID-19 phases	Mobility restrictions	Total number of unique users within a municipality
[55]	To analyze how socioeconomic status influenced mobility reductions	Urban residents	England	Early pandemic (March-April 2020 lockdown)	Mobility restrictions	Radius of gyration, average distance traveled
[56]	To examine the impact of social inequalities on mobility reduction and NPI compliance	Residents	Chile	Early pandemic (first lockdowns)	Social distancing, mobility restrictions	Average duration of trips, average distance traveled
[57]	To the impact of COVID-19 countermeasures on alcohol consumption	Students, residents, workers, and commuters	Belgium	Pre-pandemic, lockdown	Social distancing, mobility restrictions	Dynamic population proxy
[58]	To examine the impact of socioeconomic and racial inequalities on adherence to COVID-19 measures	Income levels, race (White, Black, Pardo)	Brazil	March–August 2020	Social distancing, teleworking measures	Daily isolation indexes
[59]	To examine the impact of socioeconomic inequalities on voluntary shelter-in-place adherence and mobility	General population	Chile	March–July 2020	Voluntary shelter-in-place directive	Origin-destination matrices
[60]	To study the influence of city size and socioeconomic factors on mobility behavior	General population	Colombia	Lockdown phase (March–April 2020)	Mobility restrictions	Travel frequency, average distance traveled
[61]	To investigate gender-specific behavioral responses during lockdowns	Gender (male, female), age cohorts (15–29, 30–44, 45–59, 60–74, 75+)	Austria	Pre-lockdown phase, lockdown phases (February–June 2020)	School closures, mobility restrictions	Call patterns (duration, frequency, time of calls), radius of gyration

During the initial phase of the pandemic, stringent lockdowns and travel bans were critical in reducing virus transmission and curbing population movement. For example, [28] demonstrated how mobility restrictions effectively curtailed transmission among workers and residents, while [7] highlighted a direct correlation between population outflow and the spread of COVID-19 in China. Similarly, in Ghana, [38] found that lockdowns and physical distancing measures significantly reduced transmission during the pandemic’s early stages, underscoring the importance of immediate, large-scale restrictions. However, the applicability of these interventions was influenced by demographic and socioeconomic factors. For instance, younger adults, often engaged in employment or educational pursuits, exhibited higher mobility levels compared to older adults, as noted in [29] and [33]. This disparity highlights the need for targeted NPI, such as workplace restrictions and school closures, to address mobility patterns among youth. Conversely, older adults, who generally moved less due to retirement or health reasons, were less directly affected by such measures.

As the pandemic progressed, mid-stage interventions focused on balancing public health objectives with economic activity. The effectiveness of NPI measures during this phase varied significantly across regions and income levels. In China, [30] revealed that lower-income individuals, who relied heavily on public transit, faced challenges in adhering to social distancing measures. In contrast, higher-income individuals with access to private vehicles demonstrated better compliance. Similarly, in Italy, [9] found that regions with higher education levels and older populations experienced larger mobility reductions, emphasizing the importance of tailoring strategies to regional demographics. Additionally, political beliefs influenced adherence, as [35] highlighted that individuals with differing political affiliations exhibited varying levels of compliance with stay-at-home orders in the United States.

In the later stages of the pandemic, as infection rates declined and restrictions eased, attention shifted to recovery and the revival of economic and social activities. For example, [31] analyzed mobility recovery patterns among Chinese workers, revealing significant disparities in return-to-work rates across regions and demographics, with younger and female workers experiencing slower recoveries. Likewise, [21] examined the resurgence of tourism in Beijing, noting that domestic travel rebounded strongly, driven by localized NPI that prioritized regional mobility while maintaining public health precautions.

These findings underscore that the effectiveness of NPI measures depends on the timing of their implementation and their alignment with the specific demographic and socioeconomic conditions of the target population. Lockdowns were particularly effective during the initial phase of the pandemic, significantly reducing transmission rates in densely populated urban centers, as demonstrated by [28]. Travel restrictions played a critical role in controlling intercity and international spread during different stages of the pandemic, as highlighted by [27] and [37]. In the later stages, targeted strategies became essential to support economic recovery and the resumption of mobility. For instance, localized NPI facilitated the revival of tourism in Beijing, as noted in [21], while efforts to address disparities in return-to-work rates among different demographic groups were crucial, as explored by [31]. These examples emphasize the importance of tailoring NPI to evolving pandemic conditions and the unique challenges faced by different populations.

On the other hand, Table 5 summarizes the main characteristics of the qualitative studies included in this systematic review. These studies predominantly adopt case study analyses, qualitative policy review, and thematic analysis or qualitative content analysis to examine privacy-preserving methods such as differential privacy and anonymization protocols while addressing broader ethical considerations surrounding MPND usage during COVID-19. Some studies [11,20,63] employ case study methodologies to explore the regulatory frameworks and governance mechanisms implemented in specific countries, highlighting legal barriers and socio-political challenges in MPND adoption for pandemic response. Others focus on policy reviews [5,6,14], critically analyzing data-sharing practices, transparency measures, and compliance with international regulations such as GDPR. Additionally, some studies [19,64] use thematic analysis or qualitative content analysis to investigate tensions between public health imperatives and individual privacy rights, particularly in regions with varying levels of digital surveillance acceptance.

**Table 5:** Main characteristics of the qualitative studies included in the systematic review.

Reference	Privacy considerations	Challenges in accessing MPND	Proposed solutions	Quality score	Scope
[5]	Differential privacy, GDPR compliance	Limited coordination, mistrust in data sharing	Standardized frameworks, cross-sector agreements	5	Global
[6]	Aggregated metrics (minimize identifiability)	Selection bias (uneven mobile usage)	Tailored data-sharing agreements	5	Global
[11]	Adherence to national data protection regulation, anonymization techniques	Conflict between contact tracing requirements and patient data privacy regulations	Ethical data use policies	4	Nigeria
[14]	Differential privacy, adherence to local regulations	Regulatory restrictions, legal agreements, stakeholder trust issues	Standardized privacy-compliant frameworks	4	Developing countries
[19]	Aggregated CDRs, GDPR compliance	Limited representativeness, trust gaps	Multi-operator collaboration, transparent communication	4	Ghana
[20]	Anonymization, GDPR compliance	Legal and regulatory fragmentation	Harmonized regulatory frameworks, ethical assessments	4	Europe, global
[62]	Anonymization, spatial aggregation	Minimal engagement, representation bias	Robust data-sharing policies	4	Sub-Saharan Africa
[63]	Anonymization	Tensions between public health ethics and clinical ethics	Transparent policies	3	South Korea, United States of America
[64]	Statistical thresholds, aggregation frameworks	Lack of standard frameworks, representativeness biases	Standardized aggregation syntax, privacy-preserving measures	4	Global

These qualitative studies complement the quantitative research by providing contextual depth and addressing the broader socio-ethical and regulatory dimensions that quantitative analyses alone cannot capture. While quantitative studies quantified mobility changes and assessed the impact of NPI using mobility metrics, the qualitative studies explained the underlying challenges in data accessibility, privacy concerns, and policy constraints. For instance, while quantitative findings demonstrated the effectiveness of lockdowns and travel restrictions in reducing transmission (e.g., studies analyzing origin-destination matrices and mobility indices), qualitative research highlighted barriers such as mistrust in data sharing, fragmented regulatory landscapes, and lack of standardized data-sharing agreements, which can hinder the real-time application of these insights. Moreover, proposed solutions from qualitative studies, such as standardized privacy frameworks and stakeholder engagement strategies, offer practical recommendations for improving data utilization, thereby enhancing the applicability and ethical deployment of MPND in future public health crises. The quality scores of these qualitative studies assessed using the CASP tool range from 3 to 5, with a score of 5 indicating that the study satisfies all five quality criteria, including adequately addressing ethical considerations, privacy concerns, research rigor, and bias mitigation.

### Network visualizations in COVID-19 Research

#### Network visualization of keyword co-occurrence.

**Fig 3:**
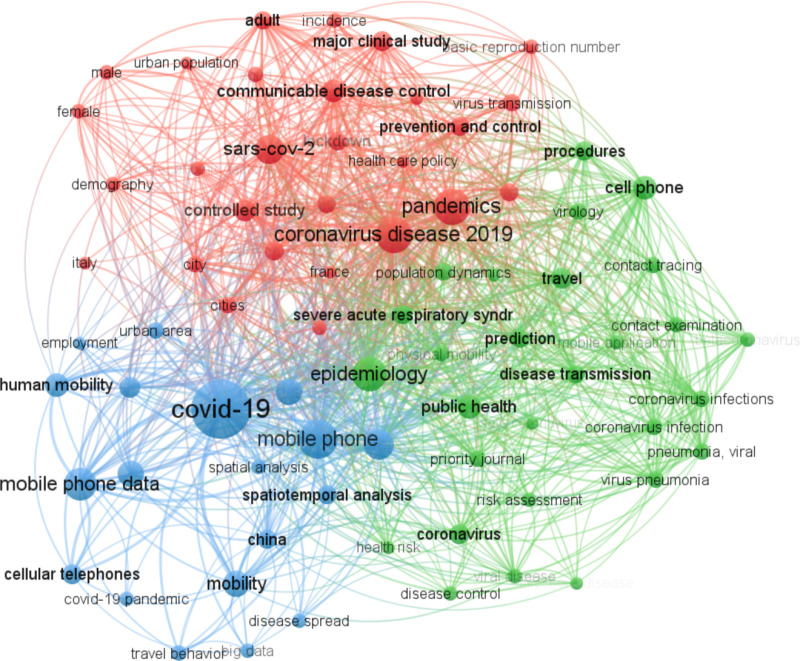
Keyword co-occurrence network in COVID-19 studies utilizing MPND. The network consists of three clusters: blue nodes focus on mobility patterns and COVID-19 spread, green nodes highlight public health and epidemiology, and red nodes emphasize government measures and NPIs. Node size indicates keyword frequency, and link thickness represents co-occurrence strength.

In Fig 3, a network visualization displays the keyword co-occurrence in studies related to COVID-19 and MPND. We gathered keyword co-occurrence data from the Scopus database and utilized VOSviewer for its graphical representation. This network offers a visual insight into the most frequently occurring keywords in the study. Here, each keyword is denoted by a node, with its size indicating its frequency of occurrence. The links between these nodes symbolize the co-occurrence of the keywords, while the weight of the link conveys the frequency of such co-occurrences. For example, the keywords “covid 19” and “mobile phone data” often appear together in several articles. They emerged as dominant search terms with 100 and 98 occurrences, respectively, boasting total link strengths of 749 and 614 when paired with other keywords.

Fig 3’s network encompasses three distinct clusters. For analytic clarity, we arranged these clusters in ascending order in a CSV file, sorting their weights (occurrences) in descending order. This arrangement displays related keywords (nodes) based on cluster, link weight, and overall link strength.

Blue Cluster: This primarily includes nodes related to the COVID-19 pandemic and its associated mobility patterns. Keywords such as “epidemics,” “disease spread,” “spatial analysis,” “travel behavior,” “spatial temporal analysis,” and “monitoring population dynamics” can be found here. They usually appear in studies investigating the link between mobility patterns and the spread of the virus.

Green Cluster: This focuses on epidemiology and infectious diseases. Keywords featured are “public health,” “risk assessment,” “viral disease,” “bacterial infections,” “beta-coronavirus,” “pneumonia,” “disease control,” and “coronavirus infection”. Studies in this cluster often focus on understanding the transmission dynamics of diseases and developing strategies for effective prevention and control measures.

Red Cluster: This cluster’s nodes emphasize government measures and NPI policies. Keywords such as “lockdown” and “social distancing” feature prominently. Additionally, factors affecting adherence to these measures, like “demographics” and “socioeconomics,” are highlighted. Research here frequently examines the social and economic impacts of government policies and how they influence public compliance with health guidelines.

#### Network visualization of co-authorship among countries.

**Fig 4:**
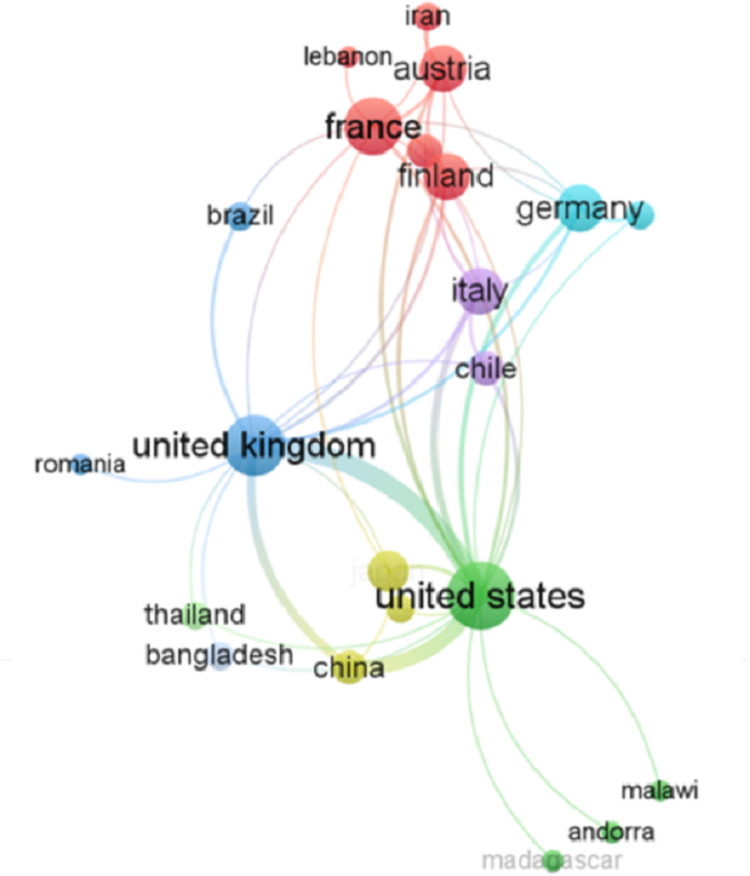
Network visualization of co-authorship patterns among countries in MPND-based COVID-19 studies. The network consists of nodes representing countries, with node size proportional to the number of publications. Nodes are color-coded to represent clusters of closely collaborating countries.

Fig 4 presents the co-authorship patterns among countries, visualized using VOSviewer, to illustrate the distribution of publications and the intensity of research collaborations in MPND-based COVID-19 studies. Each node in the network represents a country, with node size corresponding to publication volume, while the links between nodes indicate the strength of co-authorship ties. The analysis identifies over fifty countries actively contributing to MPND-related COVID-19 research.

The United States, the United Kingdom, and China are the leading contributors, producing 59, 21, and 18 publications, respectively. The United States exhibits the highest total link strength (52), indicating its central role in global research collaborations, followed by the United Kingdom (33) and China (20). These countries not only lead in publication volume but also form the core of the international co-authorship network, reflecting their influence in shaping global MPND research for pandemic response.

In addition to these dominant contributors, MPND-based research has also gained traction in several developing countries, including Iran, Madagascar, Indonesia, Thailand, Lebanon, Estonia, and Latvia. These countries have leveraged MPND as a cost-effective alternative to traditional mobility data sources, particularly in regions where censuses and surveys face logistical and financial challenges. Additionally, specific cities and states in China and the U.S., including Beijing, Shanghai, New York, Philadelphia, and Alabama, have employed MPND to enhance their efforts against the virus, as indicated by the prominence of their nodes.

Furthermore, the co-authorship visualization reveals distinct research clusters, highlighting regional collaboration patterns in MPND studies. The red cluster (the European research hub) represents an interconnected European research network, with France serving as a central hub, linking to Austria, Finland, and Iran. Notably, Lebanon and Iran are also integrated within this cluster, likely due to joint research on mobility trends and public health strategies. The strong connections between these countries indicate regional efforts to analyze pandemic-related mobility patterns and their implications for policy interventions.

The blue cluster (the Anglo-American network) highlights the United Kingdom as a significant research hub, demonstrating strong co-authorship links with Brazil and Romania. The presence of Brazil in this network suggests cross-regional collaboration, particularly in mobility analytics focused on Latin America. This cluster reflects a broad international research effort that spans both European and non-European nations, fostering knowledge exchange on mobility modeling.

The green and yellow clusters (the global research leaders) underscore the United States and China as the most prominent nodes, emphasizing their central role in MPND research. The green cluster connects the United States, Thailand, and Andorra with China and Japan, indicating extensive research on mobility analytics, economic recovery, and the application of MPND across diverse geographic regions. This cluster highlights the dominant position of the United States in mobility data-driven research, with substantial international collaborations shaping global mobility insights.

Additionally, the inclusion of countries such as Malawi, Andorra, and Madagascar underscores the increasing role of Southeast Asian and African nations in mobility research. These countries have leveraged MPND to address data limitations and explore how mobility patterns can inform policy decisions, particularly in resource-constrained environments where traditional mobility data collection methods, such as travel surveys and census data, may be less reliable. The presence of developing regions in the co-authorship network suggests a growing interest in MPND as a viable tool for public health and economic policy adaptation.

### Factors Influencing Non-Pharmaceutical Intervention Adherence

**Fig. 5:**
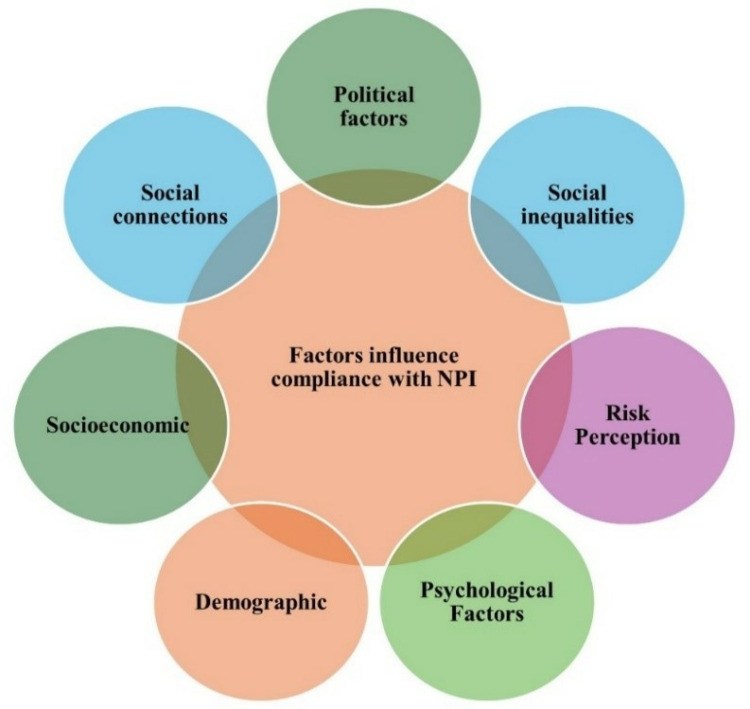
Factors influencing adherence to non-pharmaceutical interventions such as stay-at-home orders, social distancing, and travel restrictions.

As illustrated in Fig 5, multiple factors significantly influence adherence to non-pharmaceutical interventions (NPI) and their consequent impact on human mobility during the COVID-19 pandemic. Despite governments enforcing stringent regulations like travel bans, remote work policies, and stay-at-home orders, compliance with these measures exhibits regional and individual variations that are largely determined by demographic, socioeconomic, cultural, and political factors. These factors influence behavior by shaping access to resources, perceptions of risk, and the ability to comply with restrictions.

Understanding the impact of socioeconomic factors on adherence to NPI has been investigated by analyzing how socioeconomic disparities significantly influence how different populations respond to measures like social distancing, mask-wearing, and travel restrictions. In practice, socioeconomic disparities affect NPI compliance by determining an individual’s ability to work remotely, afford private transportation, and purchase protective equipment. Wealthier communities typically have greater flexibility and resources, enabling them to reduce their mobility more effectively, while lower-income groups often face structural barriers that limit their capacity to comply fully.

From a demographic and socioeconomic perspective, Liu et al. [32] found that a reduction in human mobility is strongly associated with socioeconomic factors, where Chinese cities with a lower socioeconomic level exhibited higher levels of mobility, particularly in intra-city movement intensity, compared to cities with higher socioeconomic status. Pullano et al. [33] examined the impact of demographic variables on mobility patterns amidst the COVID-19 outbreak in France, revealing a significant decrease in the mobility of individuals aged 65 years and older, in contrast to the age group of 18–64 years. These findings demonstrate that older adults, aware of their elevated risks, tend to stay home more, while younger populations—often compelled by jobs or education—maintain higher mobility and are thus less able to adhere.

Income disparities have also proven to be strongly associated with differences in mobility reductions during lockdowns. This is because higher-income individuals typically have the means to transition to remote work or use private transportation, making compliance with social distancing less disruptive to their lives. Conversely, lower-income individuals are more likely to rely on public transport or have in-person jobs that cannot be done from home, forcing them to risk higher exposure. Higher-income individuals are more likely to comply with lockdown measures due to their ability to work remotely and access private transportation, reducing the need to use public transit. Conversely, lower-income individuals often rely on public transportation and must continue working in essential or high-contact jobs, making adherence to lockdowns and social distancing measures more challenging. Huang et al. [65] found that median household income is a significant predictor of compliance with stay-at-home orders, with wealthier individuals more likely to increase their home-dwelling time. This suggests that economic privilege allows for better adherence to NPI, highlighting the need for targeted support for lower-income populations. Technically, Huang et al. [65] analyzed home-dwelling time during stay-at-home orders in twelve Metropolitan Statistical Areas (MSAs) in the United States, exploring various socioeconomic and cultural factors influencing this adherence. Notably, they found a significant positive correlation between median household income and increased home-dwelling time (rHDT), indicating better compliance among wealthier individuals. Conversely, there was a significant negative correlation between low-income households and rHDT. Additionally, a negative correlation was found between the percentage of individuals with low education levels and rHDT, while a higher percentage of individuals with graduate education correlated positively with rHDT, indicating better compliance among more educated populations. Thus, not only does income shape one’s material capacity to comply, but education level may increase awareness of pandemic risks and trust in health recommendations.

Huang et al. [65] also explored the impact of cultural factors on compliance with NPI, finding significant correlations between racial and ethnic composition and adherence to these measures. Specifically, there was a positive correlation between the percentage of the White population in a census block group (CBG) and increased home-dwelling time (rHDT). In contrast, there was a negative correlation between the percentage of Black residents and rHDT, reflecting lower compliance, possibly due to systemic inequalities and socioeconomic challenges faced by Black communities. These challenges include less access to remote-work opportunities, higher rates of public-facing service jobs, and longstanding mistrust in public health structures, all of which can reduce the feasibility or willingness to follow strict lockdowns.

Education and income levels also played a role in NPI adherence. Gauvin et al. [9] found that older and more educated individuals consistently exhibited larger mobility reductions during all three phases of COVID-19 lockdown measures. Conversely, younger adults had higher levels of movement due to employment and educational pursuits, leading to lower adherence. Liu et al. [32] found that regions with lower socioeconomic levels showed weaker responses to mobility changes, as individuals with lower incomes relied more on public transport and were often unable to work remotely due to the nature of their occupations. Garnier et al. [66] further investigate the impact of socioeconomic factors on adherence to social distancing measures during lockdowns. The results revealed that counties with higher income levels displayed significant reductions in mobility during lockdown phases. In contrast, lower-income regions struggled to reduce movement. Specifically, the proportion of poor individuals was found to be significantly related to increased travel distances (t = 11.1, p < 0.001), visitation rates (t = 7.92, p < 0.001), and encounter rates (t = 5.51, p < 0.001). (Again, these data confirm that financial constraints, job requirements, and reliance on public infrastructure collectively limit low-income communities’ capacity to stay home or maintain distancing.) This can be explained by their reliance on public transport and their inability to work remotely due to the nature of their jobs, such as in services, retail, cleaning, or agricultural labor.

On the other side, investigating cultural responses to the COVID-19 pandemic and how different groups might be affected by factors such as their religious beliefs, social connections, collectivistic, and individualistic cultures can affect their adherence to NPI. These cultural factors play an important role in shaping population behavior and response during a pandemic. In the United States, Charoenwong et al. [41] discovered that levels of adherence to COVID-19 social distancing orders are influenced by social connections, whereas [34] found they are influenced by political beliefs. Political ideology can shape how seriously individuals perceive the virus threat and whether they trust government-led interventions, thus altering their likelihood of masking, distancing, or staying home.

In countries with strong communal norms and a sense of civic responsibility, higher compliance with mask-wearing and other NPI was observed, driven by social pressure and collective consciousness. Conversely, in regions with individualistic cultures, lower compliance rates were often influenced by personal beliefs and political affiliations. For example, Lu et al. [67] studied the effect of the COVID-19 pandemic on regional culture and mobility. They found that cities with high cultural tightness (strict norms and punishments for deviance) exhibited better resilience to the pandemic’s mobility restrictions, indicating that regions with strict social norms and higher compliance could better maintain or regain mobility despite the pandemic. In contrast, cities with high dialect diversity experienced amplified negative effects. Cultural heterogeneity, reflected by diverse dialects, likely hindered coordinated public health responses, complicating NPI implementation and adherence, as heterogeneous populations may have varied responses to public health directives. In other words, shared values and cohesive communication channels can foster compliance, while cultural fragmentation can lead to inconsistencies in following guidelines. However, the predictors of adherence to different measures taken by governments in different populations may differ based on the region’s culture and the nature of the population [68].

Woodcock and Schultz [69] aim to provide valuable insights into how cultural and social factors influence NPI adherence, particularly mask-wearing during the COVID-19 pandemic. The study found that social norms play a critical role in shaping mask-wearing behavior. Social norms refer to the accepted behaviors within a group or society. When people observe that others in their immediate environment are wearing masks, they are more likely to do the same. This suggests that seeing others wear masks reinforces the behavior, creating social pressure or encouragement to conform to the norm of mask-wearing. This demonstrates that cultural factors, manifested through social norms and behaviors, are pivotal in driving compliance with public health guidelines. The study also found systematic variations in mask-wearing behavior based on political affiliation, education level, gender, age, and race/ethnicity. Mask-wearing was more prevalent among individuals in Democratic-leaning regions and those who identified as Democrats compared to Republicans. Hence, partisan identity often influenced how seriously people took official advice, with some groups more inclined to perceive mandates as essential civic duties, while others viewed them as infringements on personal freedom. This difference can be attributed to the political rhetoric and leadership during the pandemic, which varied significantly between different political groups.

In Chinese culture, the collectivism factor also plays a significant role in shaping behaviors, especially in response to public health crises. Lee et al. [70] found that this cultural value greatly influenced the early adoption of protective behaviors, such as mask-wearing, within the Chinese-Canadian community. Participants emphasized the importance of protecting not only themselves but also others in their community. This demonstrates how collectivistic values prioritize the well-being of the group, leading to higher compliance with NPI like mask-wearing to protect the community. By contrast, in more individualistic societies, personal freedom can outweigh collective norms, which may reduce mask adoption or adherence to social distancing. This finding aligns with the broader understanding that in collectivist cultures, there is a strong emphasis on community well-being and mutual protection, significantly influencing adherence to health guidelines and public health measures. Nevertheless, the adoption of mask-wearing as a cultural practice is significantly influenced by economic factors. Higher-income individuals are more likely to be able to afford protective measures such as masks, whereas those in lower-income brackets may encounter difficulty in obtaining these essential resources. Thus, economic capacity intersects with cultural values: even in collectivist cultures, a lack of financial means can limit adherence if protective tools are not accessible.

Previous findings indicate that socioeconomic and cultural variables do not operate in isolation but intersect to shape how populations respond to NPI measures. For instance, lower-income individuals, already disadvantaged by limited access to remote work and private transportation, may also belong to demographic or cultural groups that hold different norms regarding mask-wearing or perceive government restrictions differently. Conversely, wealthier or more educated groups may have stronger initial adherence due to remote-work flexibility and higher access to protective supplies, yet this adherence can be further influenced by political or social affiliations.

Cultural norms also play a compounding role in shaping responses to these socioeconomic realities. For example, collectivist cultures, as seen in parts of Asia, have a higher propensity for adherence to NPI [71] due to strong communal norms and civic responsibilities, even among low-income groups. In contrast, individualistic cultures, common in many Western countries [72], often prioritize personal freedoms over collective health measures, leading to lower compliance regardless of income levels. This interplay between economic privilege and cultural values suggests that targeted interventions must address both dimensions simultaneously.

Studies by Lu et al. [67] and Lee et al. [70] highlight these interactions. For example, cities with higher dialect diversity (a marker of cultural heterogeneity) showed amplified non-compliance with NPI, particularly in lower socioeconomic regions. Such heterogeneity often hinders communication and coordination in public health campaigns, further compounding the challenges faced by economically disadvantaged communities. Similarly, Woodcock and Schultz [69] found that political beliefs—shaped by cultural and social affiliations—intersect with income disparities to influence mask-wearing behavior. For instance, individuals in lower-income, Republican-leaning communities exhibited both cultural resistance to NPI and economic barriers, creating a dual-layered challenge to compliance.

These findings underscore the importance of intersectional approaches that address overlapping vulnerabilities. Public health policies should prioritize equity by addressing structural barriers, such as income disparities and access to resources, while tailoring interventions to align with cultural values and norms. Future research should explore dynamic models to predict adherence patterns and develop inclusive strategies for health crises.

### The role of MPND in the POST-COVID-19 era

The COVID-19 pandemic greatly affected urban areas and cities, leading to reduced human activities and interactions as a result of remote work arrangements and the temporary closure of numerous businesses. The resulting disruption presented substantial economic and commercial difficulties for the cities. Consequently, MPND has been leveraged to enhance urban resilience and facilitate the creation of data-driven urban environments.

Prior to the pandemic, urban areas thrived through conventional economic pursuits and in-person social interactions. However, the pandemic has necessitated a reassessment of these urban dynamics. In the pandemic’s wake, as it has begun to wane, there’s been an increasing interest in using MPND to aid in recovery and lay the groundwork for future development.

MPND studies aim to explore changes in travel behavior and mobility patterns as the pandemic’s impact diminishes. These studies assess mobility patterns before and after the pandemic, with the purpose of determining if movement has reverted to the ordinary patterns seen prior to the pandemic [73]. For instance, a study in China [42] aimed to analyze mobility recovery patterns and observed that there is a change in intercity mobility flows during the post-lockdown period as compared with the pattern during the pre-lockdown phase. In the post-lockdown period, the authors quantified the recovery in intercity travel and observed a significant rebound to approximately 300,000 daily trips. This figure represents a threefold increase from the lockdown period and accounts for 60% of the mobility levels recorded before the pandemic. In addition, the recovery process was not uniform across regions; it was more pronounced within regions than between them. This indicates regional fragmentation in the recovery process, where local travel within short distances (below 445 km) rebounded more quickly than longer-distance travel. This pattern was particularly noticeable between cities within the same urban agglomerations or neighboring megaregions, such as from Wuhan to Changsha and Hangzhou to Shanghai.

Another key focus is the recovery of economic activities, particularly by investigating tourist behavior and tourism recovery in the post-COVID era. A study conducted in Beijing [21] tracked the resurgence of tourism using MPND, observing a significant 40% increase in tourist influx compared to pre-pandemic levels in 2019. This analysis included data from 277.15 million tourists over three years to identify shifts in domestic tourism and intercity mobility after lockdown measures were lifted. The findings revealed an increase in domestic tourism that surpassed pre-pandemic levels, demonstrating the resilience and adaptability of urban centers in response to eased travel restrictions. Studies have shown that while there was a severe drop in tourist mobility during the lockdown periods, there was a robust recovery afterward, with intercity flows increasing significantly once restrictions were lifted. However, this recovery was uneven across different demographic groups, with elderly and female tourists experiencing a slower recovery initially but showing a quicker rebound in subsequent phases. On the other side, Liu et al. [31] explored the recovery of economic activities in China by evaluating the returning-to-work patterns of rural migrant workers (RMWs) in three major urban agglomerations post-COVID-19. This analysis helps to understand the dynamics of economic recovery in the post-COVID-19 era and how these patterns varied with location, city level, and human attributes. The study results show that the return-to-work patterns significantly differed across the Beijing-Tianjin-Hebei region, the Yangtze River Delta, and the Pearl River Delta. In March 2020, the ratio of RMWs returning to work drastically decreased to 53.29% in the Beijing-Tianjin-Hebei region, 68.42% in the Yangtze River Delta, and 68.18% in the Pearl River Delta compared to March 2019. By October 2020, the numbers in the Yangtze River Delta had fully recovered to their pre-pandemic levels, and the other two regions also saw recovery rates exceeding 95% of the October 2019 figures. Notably, the rate at which these workers returned to their jobs varied significantly by their hometowns, indicating that geographic proximity to major urban centers influenced the speed and likelihood of returning to work. Additionally, there was a notable difference in the recovery patterns based on age and gender, with younger and female workers experiencing slower return-to-work rates. The overall observation indicates that the spatial patterns of workers’ return to offices have been influenced by several factors, including workplace location, population size, gender, age, and the city tier system.

Going one step further, Yu et al. [74] aimed to examine changes in urban park use behavior during and after the COVID-19 pandemic by correlating MPND, socioeconomic profiles, and urban park attendance records. The study findings revealed a significant decline in park visitation, with over 50% fewer visitors during the pandemic. Interestingly, smaller and more remote parks experienced an increase in visitor numbers post-pandemic, indicating a shift in visitor preferences to less crowded areas. The study also highlighted the pandemic’s disproportionate impact on vulnerable populations, including females, the elderly, juveniles, and low-income groups. Notably, the elderly and low-income individuals experienced a more substantial reduction in park usage, exacerbating existing social inequalities. These findings underscore the importance of considering demographic and spatial factors in urban planning and public health strategies.

Drawing on the collective insights from these findings, it becomes evident that MPND is a valuable tool for monitoring and predicting phases of economic recovery, offering insights crucial for tailoring interventions that effectively support recovery processes. These insights are crucial for policymakers tasked with managing public health and economic strategies, especially in mitigating the impacts of future pandemics or similar crises. For example, the ability to track a recovery in tourist inflow in cities like Beijing provides a strong indication of an economic resurgence, primarily driven by a boost in local tourism.

Furthermore, the integration of MPND with socioeconomic data has revealed significant socio-economic disparities in how different populations experience and recover from public health crises. The studies highlight that vulnerable groups, such as women and the elderly, face more substantial challenges in regaining their pre-pandemic mobility levels. This finding underscores the necessity for targeted strategies that not only mitigate the impacts of pandemics on these populations but also promote the equitable distribution of resources, such as urban green spaces.

### Study taxonomy

**Fig. 6:**
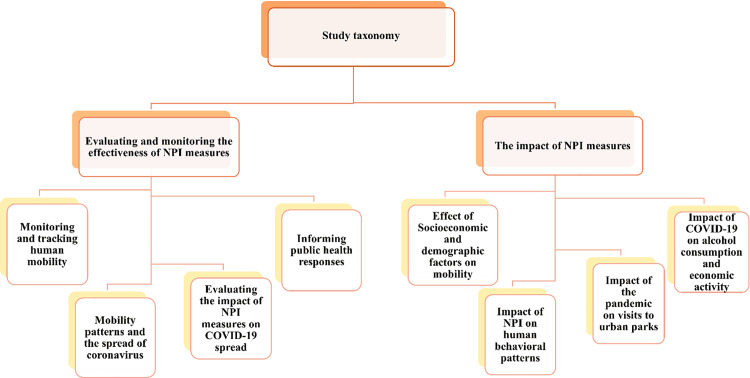
A taxonomy of COVID-19 studies utilizing MPND, consisting of two categories: evaluating and monitoring the effectiveness of NPI measures and the impact of NPI measures. Each category is further divided into subcategories to represent the diverse applications of MPND.

This section introduces a structured taxonomy of recent studies that have explored the application of MPND in guiding responses to the COVID-19 pandemic. The taxonomy is designed to categorize existing COVID-19-related studies based on their thematic alignment, with the intent of providing a useful tool for researchers and policymakers. This categorization offers a comprehensive view of the diverse applications of MPND, as illustrated in Fig 6.

The taxonomy encompasses various primary categories and corresponding subcategories, each defined by specific criteria that align with the unique applications and outcomes of MPND studies during the pandemic. During the early stages of the COVID-19 pandemic, public health authorities prioritized implementing measures to effectively reduce the spread of the virus. Non-pharmaceutical interventions (NPI), such as stay-at-home mandates, travel bans, and lockdowns, were quickly deployed worldwide. In this context, MPND provided crucial real-time insights into the impact of these measures on human mobility and dynamics.

Hence, MPND was instrumental in monitoring, tracking, and estimating changes in human dynamics and mobility patterns to evaluate compliance with NPI measures and assess the effectiveness of these interventions. For instance, analyzing whether decreases in movement are correlated with reductions in COVID-19 transmission rates provides evidence on the efficacy of NPI. If mobility significantly decreases following the introduction of NPI and this reduction coincides with a drop in COVID-19 case numbers, it suggests the effectiveness of the measures implemented. Consequently, the creation of the first category, ‘Evaluating and Monitoring the Effectiveness of NPI Policies,’ was driven by the need to understand and quantify the impact of NPI during the critical early phases of the pandemic.

While the primary focus of NPI is to curb the spread of COVID-19 by controlling physical interactions, these measures also invariably result in significant changes to daily life, affecting mental health, economic stability, and societal norms. Hence, a second category, ‘The Impact of NPI Measures on Human Lifestyles and Well-Being,’ has been created. This category encompasses studies that assess the broader impacts of travel bans, lockdowns, and other restrictions on human behavior and community dynamics. As NPI effectively reduce virus transmission through mobility restrictions, they simultaneously impose changes in lifestyle that can disrupt normal social and economic activities. This category was developed to capture the extensive range of impacts that these health measures have on different aspects of life.

#### Evaluating and monitoring the effectiveness of NPI policies (First Category).

MPND was employed to examine human behaviors and mobility patterns during the pandemic, primarily to gauge the efficacy of various NPI policies in reducing mobility and, consequently, curbing the spread of COVID-19. This data enabled an assessment of the relative effectiveness of different NPI measures, such as stay-at-home mandates, travel bans, and lockdowns, in terms of adherence and compliance. Understanding the effectiveness of these measures is crucial for informing recommendations in response to future pandemics. This category includes 25 studies (n = 25/55) that investigated the effectiveness of NPI.

#### First application type: monitoring and tracking human mobility patterns.

Controlling the spread of COVID-19 required close monitoring and tracking of human mobility, as mobility plays a pivotal role in virus transmission. NPI measures such as lockdowns, travel bans, and social distancing were implemented globally to limit mobility, reduce interpersonal interactions, and contain the virus. MPND has been an invaluable resource in assessing the compliance and effectiveness of these NPI strategies.

Lockdowns represent the most stringent form of non-pharmaceutical interventions (NPIs), characterized by significant restrictions on population mobility. Mobile phone data has proven instrumental in quantifying the impacts of these measures. For instance, Vinceti et al. [75] analyzed anonymized MPND during Italy’s nationwide lockdown and found a direct correlation between mobility reductions and decreased COVID-19 transmission rates. Their findings highlighted that mobility reductions of up to 82% in certain regions significantly curtailed viral spread, with provinces experiencing the highest reductions also seeing the most pronounced declines in case numbers. In Finland, Willberg et al. [43] investigated urban-to-rural migration patterns during lockdowns. Their analysis revealed that while overall mobility declined, rural areas experienced increased activity as individuals sought refuge from densely populated urban centers.

Additional studies reinforce the utility of MPND in understanding the impact of lockdowns. Szocska et al. [3] in Hungary demonstrated a sharp reduction in mobility during lockdown periods, with a pronounced shift toward localized movements, reflecting adherence to stay-at-home directives. Tan et al. [37] provided a detailed analysis of mobility during China’s 2020 lockdowns, leveraging data from 318 million mobile phone users. Their findings showed that lockdowns caused a dramatic 70% reduction in cross-city mobility, effectively halting large-scale population movements. This reduction, sustained for nearly three weeks after the Lunar New Year, delayed the return of over 72.89 million individuals to major urban centers such as Beijing and Shanghai.

Travel bans, while less intrusive than lockdowns, primarily targeted inter-regional and international transmission pathways. Zhou et al. [13] showed that early travel restrictions in China delayed the global spread of COVID-19 by several weeks, providing critical time for governments to strengthen preparedness. Green et al. [44] in Malawi monitored population densities and movement reductions to evaluate adherence to travel bans. Tan et al. [37] found that travel bans significantly curtailed long-distance travel in China, increasing reliance on local travel.

Social distancing measures aim to reduce person-to-person interactions by encouraging physical separation in public and private spaces. The success of these measures depends heavily on individual compliance, which is shaped by socio-economic and cultural factors. For instance, Khatib et al. [8] monitored high-density zones in Spain to identify areas with heightened transmission risks, enabling targeted interventions. Gibbs et al. [38] in Ghana demonstrated significant correlations between reduced human movement patterns and declines in infection rates. However, densely populated cities often posed challenges to effective social distancing due to the impracticality of maintaining physical separation.

Beyond tracking general population flow, MPND has also been leveraged for targeted interventions. Nisar et al. [44] and [46] tracked infected individuals and those with suspected COVID-19 infections by extracting their location and call history from MPND. Similarly, Gan et al. [39] proposed a systematic risk assessment framework focusing on higher-risk individuals by analyzing their trajectories. These targeted approaches complement broader mobility analyses by providing insights into specific transmission risks.

While individual NPI measures, such as lockdowns and social distancing, are effective in mitigating the spread of COVID-19, combining these strategies often enhances their overall effectiveness. This synergistic effect arises because different NPIs target distinct aspects of human behavior and mobility. For example, lockdowns significantly reduce inter-district travel and large-scale congregation, as observed in the Vodafone mobility data analyzed by Gibbs et al. [36]. In contrast, social distancing limits close-contact interactions within districts. Gibbs et al. specifically examined the effects of multiple NPIs implemented in Ghana during the early stages of the pandemic, including partial lockdowns in the Ashanti and Greater Accra regions, movement restrictions, and mask mandates. By leveraging Vodafone mobility data and Google’s residential mobility data, the authors demonstrated a strong association between reductions in mobility and a decline in the effective reproduction number (Rt), particularly during periods of stringent interventions.

Gauvin et al. [9] similarly analyzed the effects of different types of NPIs, including lockdowns and social distancing orders, on mobility patterns across regions and urban centers. Their findings emphasized that lockdowns primarily curtailed large-scale movements, particularly in densely populated urban areas, while social distancing measures further reduced localized mobility by restricting close-contact interactions. This layered approach was shown to enhance the overall reduction in mobility, especially in urban centers where transmission risk was highest.

Hsiehchen et al. [35] extended this understanding by investigating mobility reductions across U.S. states during the pandemic. They identified significant variability in adherence to NPIs, with reductions ranging from 34% to 69%. Comprehensive strategies, such as stay-at-home orders, mask mandates, and closures of nonessential businesses, yielded the highest mobility reductions. However, the effectiveness of these NPIs was influenced by sociopolitical factors, such as political alignment. States with higher proportions of Republican-leaning populations demonstrated lower adherence to NPIs, even when stringent measures were enacted. This underscores the role of sociopolitical and contextual factors in shaping the effectiveness of NPI strategies.

Although combined strategies demonstrate significant potential in reducing mobility and mitigating disease transmission, their effectiveness is not uniform across contexts. Factors such as geographic clustering, political dynamics, and socioeconomic conditions play critical roles in shaping outcomes. While studies highlight the benefits of NPIs, these associations often fluctuate over time and between regions. Effectiveness is often observed to be highest during the early stages of an epidemic but can diminish as circumstances evolve. These findings underscore the need to tailor NPI combinations to specific epidemic phases, recognizing that diverse factors significantly influence their outcomes. The variability across regions and timing highlights the importance of considering political, social, and economic contexts when designing multi-layered interventions to maximize their adaptability and impact.

#### Second application type: investigating the correlation between mobility patterns and the transmission of the coronavirus.

Seven studies (n = 7) investigated the relationship between mobility patterns and the transmission of the coronavirus. Jia et al. [7] and Zhou et al. [13] were among the first reports in the literature of an investigation of the human mobility dynamics and the spread of COVID-19 during the pandemic’s early phases. Zhou et al. [13] developed an epidemic compartmental model to evaluate the effectiveness of travel and mobility restrictions on the dynamic behavior of COVID-19. They found that a decrease in people’s movement had a substantial effect on reducing the number of COVID-19 cases. Specifically, a reduction in human mobility of 20–60% was shown to help reduce the peak number of infected subjects by 33%, thus delaying the peak of the pandemic by up to two weeks.

Berke et al. [12] and Badr et al. [28] introduced estimation and prediction models that utilized both MPND and COVID-19 infection data. This dual-data approach aimed to explore the connection between movement patterns and transmission rates of COVID-19 and how shifts in mobility influenced the virus’s spatial spread. MPND refined the accuracy of detecting evolving population patterns by quantifying trips between origins and destinations and by estimating daily departures from cell towers. Simultaneously, infection data facilitated the visualization of daily reported cases.

In Spain, authors [2] developed a geographic information system that integrated two datasets: MPND, representing human movement patterns, and daily reports of COVID-19 cases. The system aimed to visualize the mobility patterns of individuals on a daily basis in combination with COVID-19 incidence on a single geographical layer to allow observation and tracking of any changes and to thus investigate the influence of mobility patterns on the transmission of COVID-19. In Bangladesh, Cowley et al. [47] aimed to monitor population movements and trace the geographical distribution of SARS-CoV-2 variants. This was accomplished by analyzing mobility data from Facebook users and MPND and correlating it with genomic data, which includes the genetic sequences of the SARS-CoV-2 virus. This correlation approach facilitates the visualization of real-time updates of the SARS-CoV-2 genome sequences and its variants and enables a comprehensive understanding of how genetic variations within the virus relate to population movements and the spatial spread of COVID-19. Finally, in Japan, Kawakami et al. [48] applied cross-correlation analysis to demonstrate whether fluctuations in human mobility have a direct impact on the acceleration or deceleration of COVID-19 infection growth. The study found that in the first half of 2020, there was a strong connection between the rate of change in COVID-19 infections and human mobility, and this connection was at its highest, with a value of 0.551.

#### Third application type: informing public health responses to the pandemic and developing further actions (Preparedness).

This section presents seven studies (n = 7) that explore the theoretical application of MPND to enhance public health responses during the COVID-19 pandemic. The degree of preparedness shown by governments before the outbreak substantially impacted the enforcement of various NPI, including those related to mobility and travel. In this regard, Olivera et al. [5] and Grantz et al. [6] underscored the importance of promoting collaborative efforts between governments, mobile phone operators, and public authorities. They advocate for the establishment of interdisciplinary teams to ensure timely access to MPND, bolstering public health initiatives. This collaborative approach not only expedites responses to outbreaks but also enables the evaluation of the effectiveness of intervention measures.

Other studies [11,19,20,4962] discussed the potential opportunities and challenges that mobile phone companies, decision-makers, governments, and public health experts should take into account when considering the ethical and effective utilization of MPND. The potential opportunities involved investing more efforts into educating government agencies about the possible applications of MPND in controlling COVID-19 and creating project frameworks for public-private research that foster productive and trusting research environments. Challenges, on the other hand, revolved mainly in terms of legal and ethical implications and potential hurdles related to data access.

#### The impact of NPI measures (Second Category).

This section focuses on studies (n = 18) that discuss the impacts of NPI policies on human lifestyles and well-being. These include their impact on the global economy, the exacerbation of social and racial inequalities, the psychological impact on individuals, and the damage to cities resulting from the decline in tourism activities.

Romanillos et al. [50] sought to assess the effects of NPI on Madrid’s dynamics by analyzing how different land uses (such as residential, commercial, and industrial) influenced population distribution. Notably, during the lockdown periods, significant declines were observed in activities related to commerce, entertainment, and education. Lanza et al. [25] investigated the influence of COVID-19 lockdowns and social distancing measures on human behavior in Italy. Their finding showed that these movement restrictions led to an increase in near-home tourism as tourists preferred places close to their homes, as well as an increase in remote working.

In terms of the economic impacts of the COVID-19 pandemic, a great deal of damage to the tourism economy has occurred due to changes in tourist mobility, such as international travel restrictions. Yu et al. [21] explored the alterations in tourist movement patterns at the onset of the COVID-19 pandemic. They found that during the initial wave of 2020, Beijing experienced a sharp decrease in domestic tourist numbers. Notably, tourists aged 19–39 years old exhibited a swifter recovery compared to older age groups.

Toger et al. [51] aimed to explore the negative impacts of the pandemic on individuals’ daily activities, such as lunch meetings, restaurant visits, and public transportation usage. They documented a drastic reduction in such individual activities during the pandemic. A further impact of COVID-19 on human lifestyles and well-being behaviors was found by many researchers in changes in human travel behaviors based on travel restrictions and bans. For instance, Pan and He [30] assessed COVID-19’s impact on reducing travel mobility patterns in China. Their study revealed a more significant impact on non-commuting travel (trips for shopping, visiting friends, and social activities) than on commuting travel (travel solely between home and school or work). Furthermore, a greater decrease in mobility and travel frequency was observed in lower-income groups, migrants, and the elderly than in higher-income groups and local residents. The latter group generally had better access to safety measures such as private cars, higher-quality masks, and hand sanitizer.

#### COVID-19’s impact on society and economy.

The global COVID-19 pandemic significantly influenced human behavior and economic activities across the world. Table 6 summarizes the various applications derived from MPND to examine the pandemic’s impact on aspects such as economic activity, human behavior, tourism, and travel.

**Table 6:** Overview of studies investigating COVID-19 effects on human behaviors and city dynamics.

Reference	COVID‑19 impact examined	Location	Description
[52]	Visitation behaviors.	USA	Investigation of COVID-19’s effects on park visitation.
[53]	The impact of NPI on individual behavioral habits such as going to work, hanging out, purchasing, and traveling.	Japan	Although the Japanese government’s NPI measures were voluntary, they were effective in reducing the mobility of the population and thus had a significant impact on human lifestyles and activities. Notably, shopping activity showed a sharp decline of 75.2%, while working activities decreased by 47.8% following the government’s remote working directive.
[15]	Effects of the pandemic on urban park visits.	South Korea	The COVID-19 pandemic has resulted in both negative and positive changes in human behavior. Visiting natural green spaces like parks, rivers, and forests plays a crucial role in preserving people’s overall well-being and aiding in the management of mental health challenges like anxiety and depression. Consequently, there was a notable increase in the number of visitors to these parks during the pandemic.
[54–56]	Economic activity.	Chile, England, and Latvia	The alterations in human movement patterns due to lockdowns and travel restrictions not only affected human lifestyles, habits, and quality of life but also caused a substantial decline in global economic activity. These changes led to an increase in socio-economic inequality, a rise in poverty rates, and a worsening of income disparities. To analyze the socio-economic landscape during the pandemic, many studies utilized phone records to capture human activity and dynamics alongside socio-economic data containing details about income, education, employment, income, address, age, and gender to analyze the socioeconomic status of individuals. For instance, Do Lee et al. [56] explored the effects of social inequalities and socio-economic differences in England during the pandemic, revealing that areas with non-English-speaking residents, individuals engaged in lower-middle-class occupations, and high-income households experienced the most significant reductions in mobility, particularly when compared to regions with a higher proportion of self-employed workers.
[57]	Alcohol consumption.	Belgium	Another negative change in physical and mental health behaviors due to lockdown and restrictions on physical contact and mobility, particularly where recreational, sporting, and cultural activities were suspended, was the increased consumption of alcohol. This study investigated changes in the consumption patterns of alcohol during the pandemic, determining that while alcohol consumption decreased during the exam season, it was higher during the holiday season.
[9]	Impact of mobility restrictions.	Italy	This study investigated the potential effect of socio-economic determinants on adherence to mobility restrictions in Italy, with results that suggested that areas characterized by a larger agricultural workforce were significantly associated with smaller reductions in mobility during lockdowns. This observation can be attributed to the need to ensure food provisioning. Areas characterized by workers in the industrial and service sectors experienced higher levels of mobility reductions during the lockdown, as all non-essential companies were urged to let employees work remotely or to close if this was not possible. In addition, larger reductions in mobility were significantly associated with older and more educated populations.
[58]	Social and racial inequalities and their social and psychological impact.	Brazil	This study investigated the effects of socio-demographic characteristics on the population in São Paulo during the epidemic. The study revealed that patients residing in the least wealthy regions and receiving medical care in public hospitals exhibited a higher probability of mortality in contrast to patients residing in affluent regions and receiving medical care in private hospitals.
[59, 60]	Socioeconomic inequality.	Chile, Colombia	These studies investigated how socioeconomic status differences, as found between low- and high-income areas in developing countries such as Chile and Colombia, affected human compliance with NPI such as lockdowns and shelter-in-place restrictions. The results showed that locations with higher socioeconomic levels and more commercial and industrial locations were associated with larger reductions in human mobility as compared with low-income areas.
[61]	Stress and anxiety.	Austria	This study aimed to investigate human behavioral changes during lockdowns with respect to social relationships (calling patterns) and movement patterns based on extracting call and spatiotemporal features, including the number of calls, timestamps, call duration, numbers of outgoing and incoming calls, individual trajectories, and identification of the user’s home location. The findings indicated notable shifts in communication patterns during lockdowns. There was a remarkable increase in female-female calls, surging by as much as 140%, while calls between females and males, as well as males among themselves, saw substantial growth, with respective increases of up to 81% and 97%.
[33]	Socioeconomic and demographic impacts of lockdown measures.	France	Previous studies considered the effects of socioeconomic variations and NPI measures such as lockdowns having a significant impact on reducing human mobility; however, this study investigated other factors such as the labor market, demographics, risk perception, and socioeconomic indicators. These indicators were found to play an essential role in the levels of reduction seen in French population mobility. Older people (65 years of age or older) decreased the number of trips taken, with most traveling only over distances shorter than 100 km. In addition, employees at hotels, food services, and finance and insurance firms were more negatively affected by the lockdown because of a large reduction in mobility.

### Ethical implications and privacy concerns

The availability of MPND provides invaluable insights into human behavioral patterns, significantly aiding applications such as crime prevention, optimization of public transportation, and preparedness for emergencies like natural disasters. However, the use of MPND comes with critical ethical and privacy challenges, as mobility data often contains sensitive personal information, including home locations and frequently visited places. These concerns have been amplified during the COVID-19 pandemic, where the utilization of MPND to monitor and assess the impact of NPI raised questions about the balance between public health benefits and individual privacy rights. While researchers and policymakers have recognized the societal benefits of MPND, they have also emphasized the necessity of implementing robust privacy-preserving measures to safeguard users’ data.

One of the foremost privacy concerns surrounding MPND is the risk of re-identification despite anonymization techniques. Even when aggregated, mobility patterns can reveal individual identities when cross-referenced with external datasets. Studies [11,22] have demonstrated that even a small subset of mobility data can be re-identified with high accuracy, raising concerns about potential misuse by third parties, including government agencies, advertisers, and private companies. Additionally, MPND carries function creep risks, where data initially collected for public health or mobility studies could later be used for unintended purposes such as surveillance, profiling, or law enforcement. This raises legal and ethical questions about informed consent and whether individuals should have greater control over how their data is used.

To address these challenges, various solutions have been proposed in MPND research. Privacy-preserving techniques [64], such as statistical thresholds, differential privacy, and secure multiparty computation, have been recommended to anonymize mobility data effectively and limit the risk of re-identification. However, these methods often involve trade-offs—greater privacy protection can lead to a loss in data utility, which affects the granularity of mobility insights [4].

Data use agreements (DUAs) [62] have also emerged as a key mechanism for regulating the sharing of sensitive mobility data, ensuring accountability among stakeholders in the public, academic, and private sectors. During the pandemic, Anom [63] discussed these issues from a legal and ethical perspective, aligning recommendations with the core values of clinical ethics, such as non-maleficence, beneficence, and autonomy. Similarly, Kishore et al. [64] emphasized the importance of combining technical safeguards, like differential privacy, with enforceable DUAs to enhance trust and transparency in MPND usage.

Regional examples further illustrate how data privacy frameworks can be tailored to specific legal and cultural contexts. In Nigeria, for example, Ekong et al. [11] emphasized the importance of collaboration between public authorities, mobile network operators, and technology firms in compliance with the National Data Protection Regulation. This collaboration ensures that patient data privacy is preserved while granting access to MPND for critical analyses. Measures such as secure on-premise data processing and strict adherence to privacy standards have been highlighted as essential components of this approach. Similarly, in Ghana, Li et al. [19] discussed a collaboration between Vodafone Ghana and the Ghana Statistical Service, which was governed by multiple agreements adhering to Ghana’s data privacy laws. Under these agreements, MPND analysis is conducted exclusively on Vodafone Ghana’s premises, and only aggregated and anonymized data is shared externally. These case studies demonstrate how ethical and legal considerations can be successfully integrated into MPND applications.

Despite these advancements, significant research gaps remain. One major challenge is the lack of global standardization in data privacy frameworks, which creates inconsistencies across regions and hinders international collaborations involving MPND. While regional guidelines such as the GDPR in Europe offer strong protections, many countries lack comparable frameworks, raising concerns about the scalability of MPND applications in global public health initiatives. Additionally, privacy-preserving methods like differential privacy often come at the cost of reduced data utility, limiting their effectiveness for high-resolution analyses. Addressing this trade-off between privacy and utility remains a critical area for future research.

The ethical challenges of using MPND also have direct implications for research findings. Many studies analyzing human mobility and NPI compliance during COVID-19 relied on aggregated data provided by private companies (e.g., telecommunications firms, social media platforms), which raises concerns about data accessibility, transparency, and potential biases. For instance, datasets collected from app-based mobility sources (e.g., Google, Facebook, Apple) primarily represent smartphone users, potentially excluding lower-income populations, the elderly, or rural residents with limited mobile device usage. Similarly, CDR-based mobility datasets are often controlled by mobile network operators, restricting access and potentially introducing selection bias in research outcomes.

Hence, building public trust in MPND applications remains a significant challenge. Without clear policies on data collection, retention, and access, public resistance can undermine even well-intentioned public health and policy initiatives. Transparency in data governance, ethical data-sharing frameworks, and robust enforcement mechanisms are essential to ensuring accountability and fostering trust. Public confidence in MPND usage can be strengthened through clear communication about how mobility data is utilized, who has access, and how privacy protections are implemented. Additionally, advancing privacy-preserving technologies should be prioritized to develop hybrid approaches that integrate encryption, anonymization, and multiparty computation, allowing for the ethical and effective use of MPND while maintaining privacy safeguards.

Despite the progress made in privacy-preserving methodologies, unresolved ethical concerns continue to challenge MPND research and application. A unified approach that integrates legal oversight, technological advancements, and ethical considerations is essential for ensuring that MPND is used in a manner that benefits society while protecting individual privacy. The lack of harmonized global standards remains a major obstacle to large-scale MPND applications, as privacy frameworks differ widely across jurisdictions. Developing standardized international regulations could help facilitate responsible data-sharing for public health and policy purposes while maintaining consistency in privacy protections. Future research should also focus on refining encryption and anonymization techniques to maximize data utility without compromising privacy, as well as promoting interdisciplinary collaboration between policymakers, ethicists, and technologists to establish robust ethical guidelines for MPND applications.

## Discussion and future directions

The study underscores that human mobility patterns play a pivotal role in controlling and combating the spread of COVID-19, especially in the absence of pharmacological interventions. As such, extensive efforts have been mobilized to aid governments, decision-makers, and public health authorities in leveraging mobile phone network datasets—a strategy that has proven effective under current circumstances.

Many applications have been derived from MPND to control and fight COVID-19, starting with monitoring the changes in human movement and tracking COVID-19 patients. Other applications include investigating the consequences of COVID-19 on the tourism sector, gauging the impact of mobility restrictions on virus transmission, assessing the impact of socioeconomic inequality on the transmission of the COVID-19 virus, and investigating behavioral differences during lockdown. The diversity of applications of these methodologies is contingent upon the specific objectives of the study and the different aspects of human behavior under investigation, including mobility metrics and spatiotemporal features extracted to describe human behaviors.

For example, Liu et al. [29] seek to track changes in two daily human activities: dwelling and working activities. To depict these activities, the authors extracted users’ spatiotemporal information, such as the number of days a user stayed at home or another location, the number of days a user was active during a week, and the distance between these locations. These attributes allow the detection of users’ dwelling and working locations, in which the home location has been identified as the most contacted cell tower during dwelling time (22:00–06:00) for at least three hours and the workplace has been identified as the most contacted cell tower during working time (09:00–12:00 and 14:00–17:00).

Romanillos et al. [50] aim to capture additional human activities such as transport, culture, education, and commerce based on spatiotemporal characteristics that visualize the time they remain in one location (stay times), the number of trips per week based on estimating origin-destination matrices (the origin and destination of a trip) for each user, and the sum of the average weekly trips of users. Estimating origin-destination metrics (or origin-destination mobility metrics) has been widely used in the literature to capture different aspects of human mobility patterns and dynamics during the pandemic. Heydari et al. [26], for example, depicted inter-regional mobility in Finland; Gan et al. [39] depicted dynamic population flow in China; Zhou et al. [13] depicted daily activities in Shenzhen, China; Ponce-de-Leon et al. [2] captured daily population mobility descriptions in Spain; Lai et al. [33] captured changes in travel behavior in Chinese cities; Jia et al. [7] tracked human mobility outflow in Chinese cities; and Kawakami et al. [48] captured human mobility flow (both intra- and inter-prefectural) in Japan.

Identifying “home” has been a recurrent theme in literature. Defining and identifying places like home, workplace, and areas of recreation is fundamental to mobile phone data research, often serving as the foundational step for subsequent analyses [22]. The definition of home and work locations has been established based on two distinct criteria. Specifically, the place of residence has been determined as the location that is visited most frequently during the evening hours, while the workplace place is identified as the location that is most commonly visited during the day hours, as reported in [22]. Empirically, the process of identifying an individual’s place of residence involves designating a single-cell tower as the residence’s location. So, the cell tower that has been most constantly contacted during the nocturnal period, spanning from 9 p.m. to 7 a.m., is considered to be the closest representation of where they live.

Some researchers, such as Lanza et al. [25], have identified home locations to capture two aspects of human activities: near-home tourism activities and remote working activities. Pan and He [30] detected individuals’ homes and workplaces to investigate the impact of COVID-19 on human travel patterns. While studies [3,43,51] detected residential areas for assessing individuals’ compliance with NPI measures, such as stay-at-home mandates.

The results also show that NPI measures significantly reduce human movement and dynamics. A large swathe of research has focused on leveraging MPND to monitor, track, and estimate changes in human dynamics and mobility patterns to evaluate human compliance with NPI measures. This was followed by studies that utilized MPND to investigate the impact of COVID-19 lockdowns and mobility restrictions on human behavior, travel patterns, city dynamics, and economic activity. In addition, we also found that factors such as demographics, political party affiliation, socioeconomic inequality, and racial inequality had a significant impact on individuals’ compliance with NPI measures, including social distancing and stay-at-home guidelines.

Furthermore, the effectiveness of NPI during the COVID-19 pandemic was influenced by an intricate interplay of demographic, socioeconomic, and cultural factors, creating regional disparities in adherence and outcomes. For example, socioeconomic conditions significantly dictated compliance with NPI like social distancing and lockdowns. Liu et al. [32] observed that cities with lower socioeconomic levels in China exhibited higher intra-city movement intensity, indicating weaker adherence to mobility restrictions compared to wealthier regions. Similarly, Huang et al. [65] highlighted a strong correlation between higher median household income and increased home-dwelling time during stay-at-home orders, underscoring economic privilege as a key enabler of compliance. In contrast, lower-income individuals often faced barriers to compliance, such as dependence on public transportation and the inability to work remotely, as noted by Garnier et al. [66]. These disparities reveal that economic resources are critical to enabling populations to adhere to stringent NPI, suggesting the need for tailored interventions that address income-based inequalities.

In addition to socioeconomic disparities, cultural factors also played a pivotal role in determining the effectiveness of NPIs across different regions. In collectivist societies, such as China, a heightened emphasis on community well-being fostered better adherence to health measures, as observed by Lee et al. [47], who found that collectivist values prioritized protecting others through actions like mask-wearing. Conversely, in individualistic cultures, personal beliefs and political ideologies often played a more substantial role in shaping compliance. For instance, Charoenwong et al. [41] noted that social connections significantly influenced adherence to distancing measures in the United States, while political affiliations created systematic variations in mask-wearing behaviors. Lu et al. [44] demonstrated that cities with higher cultural tightness, characterized by strict norms and penalties for deviation, exhibited greater resilience to mobility restrictions. These findings underline the importance of considering cultural and societal norms when designing NPI, as adherence may depend on the alignment of public health strategies with regional values and behaviors. To further illustrate these differences, Huang et al. [65] found that in U.S. metropolitan areas, compliance with stay-at-home orders was strongly correlated with higher household incomes and educational levels, underscoring the role of economic privilege in enabling voluntary adherence to NPI. In contrast, regions with lower socioeconomic levels faced challenges in compliance, necessitating stricter lockdowns to mitigate the spread of COVID-19.

While MPND records a larger sample size of population activities due to the wide reach of telecommunication networks, it is subject to selection bias, as it only captures data from users carrying smartphones. Consequently, achieving a comprehensive picture of population activity and mobility patterns necessitates incorporating additional data sources. Correlating mobile phone data with external mobility data—such as social media platforms (e.g., Facebook and Twitter), travel card systems, GPS data, censuses, and surveys—is essential for improving the monitoring of mobility changes and tracking human behavior, potentially achieving near-total population coverage.

Throughout the COVID-19 pandemic, various mobility datasets, including MPND, smartphone-based GPS tracking data, travel smart card data, COVID-19 apps, travel surveys, and census data, have played a crucial role in monitoring human mobility, informing public health responses, and assessing the effectiveness of NPI [5,6]. Among these data sources, MPND stands out due to its extensive reach, capturing data at national or even global levels through the widespread use of smartphones and the rapid growth of telecommunication networks [22]. This broad coverage facilitates comprehensive insights into population-level mobility patterns [4], provides real-time observations of regional movement, and allows for the assessment of how mobility restrictions affect different demographic groups.

Furthermore, the ability of different data collection methods to record active and passive location details plays a critical role in capturing human mobility patterns during the COVID-19 pandemic. MPND, derived from cellular network interactions, captures both active and passive data [4]. Active data collection occurs when users make calls or access the Internet, while passive data collection happens as devices periodically connect with cell towers for signal maintenance. This duality allows MPND to offer extensive coverage and continuous temporal patterns, making it invaluable for assessing population-level mobility trends and the effectiveness of non-pharmaceutical interventions across wide geographic areas. Travel surveys and travel smart card data, in contrast, generated actively when passengers used their cards for public transit access, offering valuable insights into travel patterns within the covered transit networks. In essence, the choice of data source impacts the scope and depth of mobility analysis. MPND provides broad coverage and insight into general mobility trends important for large-scale public health strategies. However, the scope of these data types is inherently limited. Travel smart card data is geographically confined to areas with electronic transit systems, which do not capture mobility outside the public transit network. Similarly, travel surveys, although they provide detailed demographic and travel-specific data, typically cover a relatively representative sample of the entire population and focus mostly on travel-related activities.

While MPND has demonstrated its effectiveness in large-scale mobility monitoring, its spatial resolution remains a key limitation, as mobile device locations are only approximated to the coverage areas of cell towers, binding geographic data to the location of base tower stations. In contrast, GPS data and COVID-19 apps offer higher spatial resolution, which is critical for detailed tracking of individual movements [76,77]. Such precision proves invaluable in public health applications, particularly contact tracing, where accurately determining individuals’ locations and proximity to others can significantly enhance isolation measures and mitigate virus transmission.

Moreover, in terms of timeliness, coverage, and scope, MPND outperforms traditional datasets in real-time mobility tracking because it passively collects location data from mobile phone users, capturing human movements continuously rather than at pre-specified intervals. This makes MPND ideal for assessing the immediate effects of NPI, mobility restrictions, and economic disruptions during health crises, whereas census data, travel surveys, and smart card records offer lagging, event-dependent, or geographically constrained snapshots of mobility. However, privacy considerations, data anonymization processes, and regional variations in network coverage require complementary data integration with higher-precision datasets such as GPS, COVID-19 apps, and travel card data to enhance analytical accuracy. Integrating these data types is essential to addressing biases and improving analytical accuracy, enabling more robust and comprehensive public health strategies.

In conclusion, the utilization of MPND extends beyond its application in pandemic response, presenting a transformative opportunity for shaping public policy in urban planning, healthcare, and economic forecasting. During the pandemic, MPND enabled real-time monitoring of population movements and adherence to NPI guidelines. However, future research should focus on leveraging MPND to capture large-scale mobility patterns due to its ability to record users’ interactions within a mobile network, including timestamps, durations, and geographic coordinates. This capability provides policymakers with critical datasets for designing adaptive and resilient urban environments.

For example, during the COVID-19 pandemic, MPND proved invaluable in monitoring compliance with NPIs and enhancing public health surveillance and intervention. It provided real-time insights that allowed policymakers to rapidly adjust strategies, improving the management of virus transmission. This experience has established a precedent for using MPND in future public health emergencies. The methodologies developed and the empirical findings gained during the pandemic are applicable to a wide range of public health challenges, including future infectious disease outbreaks, natural disasters, and urban health crises. For instance, MPND applications used to monitor population movement during pandemics can also be adapted to manage evacuation processes during emergencies. By analyzing real-time MPND data, authorities can determine the most efficient evacuation routes, predict congestion areas, and ensure the safety of vulnerable populations. Additionally, MPND can be utilized to predict the spread of seasonal influenza by monitoring travel patterns during peak movement periods, improving proactive health interventions.

Beyond public health applications, MPND also plays a crucial role in urban planning by optimizing public transportation networks, infrastructure development, and spatial planning. By analyzing population movement patterns, city planners can identify areas requiring improved connectivity, manage traffic congestion, and design more sustainable urban layouts that respond effectively to population growth and evolving mobility needs. The integration of MPND into urban development strategies ensures that cities can adapt to long-term shifts in mobility trends, creating resilient and efficient infrastructures.

Furthermore, insights gained from applying the MPND framework during the COVID-19 pandemic have significant policy implications. The data-driven approaches developed can be leveraged to assess the effectiveness of various public health interventions. For example, findings highlight the impact of socioeconomic disparities on public health measure adherence. Understanding these disparities enables policymakers to develop targeted strategies that mitigate the socioeconomic consequences of public health crises, ensuring equitable access to healthcare and mobility resources.

From an economic forecasting perspective, MPND provides valuable insights into economic resilience by tracking business recovery, workforce mobility, and regional economic activity. Post-pandemic economic analysis using MPND has demonstrated its ability to assess labor market dynamics, consumer foot traffic in commercial areas, and tourism sector recovery. Enhancing economic resilience through MPND analysis involves monitoring and predicting economic risks related to community depopulation and business disruptions during health crises. MPND can track and analyze the causal impacts of COVID-19 on economic vulnerability by observing changes in mobility patterns—such as declining foot traffic in commercial areas or shifts in transportation usage. These insights allow analysts to assess sectoral economic health and identify regions at higher risk of long-term economic decline.

## Conclusions and limitations

Although pandemics and infectious diseases can be managed and controlled with antibiotics and antivirals, big data technologies, including MPND, have played a vital role in tackling and slowing the spread of COVID-19. The unique spatiotemporal digital traces inherent in MPND make it especially suited to monitor changes in human mobility behaviors, observe the dynamics of social distancing behavior, evaluate population-level adherence to NPI such as lockdowns, and travel restrictions, and investigate the effects of NPI on slowing the spread of SARS-CoV-2. Upon examination of the aforementioned applications, it is evident that MPND represents an effective solution to substantially reduce the risks associated with COVID-19.

In this paper, we survey the methods and techniques harnessed by MPND to fight the spread of COVID-19. We also delve into the concerns about protecting individual privacy and how this has been an obstacle in many countries, as well as strategies that should be considered to overcome data access challenges. Furthermore, this study encompasses a review of numerous mobile phone network data-driven applications, such as monitoring and tracking human mobility, investigating the correlation between movement patterns and the spread of coronavirus, investigating the effect of NPI on containing COVID-19, investigating the impact of NPI on human behavioral patterns, and investigating the effects of socioeconomic factors on human compliance with NPI policies.

However, a significant limitation of MPND lies in the lack of transparency in pre-processing methods used by data providers. Currently, pre-processing methods for mobile phone location data often function as black boxes, with proprietary workflows for data cleaning, aggregation, and anonymization potentially introducing biases that are obscured from researchers. These hidden processes pose challenges to the reliability and reproducibility of analyses, particularly in studies of mobility patterns and non-pharmaceutical interventions. To address this, we recommend adopting standardized pre-processing protocols across mobile network operators, data providers, and research organizations. This standardization would ensure consistency in data handling and comparability across studies, enhancing the overall credibility of findings. Additionally, requiring data providers to disclose essential details about their pre-processing workflows would improve transparency, allowing researchers to identify and mitigate potential biases. Validating MPND against external benchmarks, such as census or survey data, can also enhance reliability and address biases introduced during pre-processing.

While our research adheres to standard SLR methodologies, the results are not all-inclusive and should not be seen as a complete summary of all relevant studies. First, the survey solely encompasses research conducted within the timeframe of 2020–2023. Second, scholarly publications written in languages other than English have been excluded. Finally, studies that apply mobile phone data that was not generated by mobile network operators, such as phone application data, GPS data, or other mobile phone app-based data, have been excluded due to the plethora of literature and the selected study goal.

In light of the reviewed applications, challenges, and limitations of MPND, our findings emphasize the valuable role of MPND in guiding public health strategies, urban planning, and economic recovery. To maximize its utility in future health crises, policymakers and practitioners should adopt the following strategic approaches.

First, public health authorities should establish regulatory frameworks for the real-time integration of MPND into epidemiological surveillance systems. Spatiotemporal insights derived from MPND can assess the effectiveness of non-pharmaceutical interventions, detect outbreak hotspots, and inform targeted public health responses. Future applications should emphasize the development of adaptive intervention models that enable rapid responses to emerging health threats while ensuring compliance with data protection regulations.

Second, governments and urban planners should leverage MPND to enhance infrastructure resilience and emergency preparedness. Findings indicate that lower-income individuals are disproportionately affected by mobility restrictions during crises, whereas higher-income groups benefit from private transportation and remote work flexibility. To address these disparities, urban mobility policies must be adaptable to rapidly changing conditions. Integrating MPND-driven mobility insights into transportation planning, city infrastructure, and public service accessibility will allow for the development of urban environments that respond effectively to population movement fluctuations. The post-pandemic shift in commuting behavior also necessitates transport systems that accommodate hybrid work models while ensuring equitable access to mobility options.

Third, economic policymakers should utilize MPND to track economic recovery, labor market shifts, and consumer behavior patterns. Analyzing workforce mobility and commercial activity in real time enables the implementation of targeted economic stimulus programs, business recovery strategies, and supply chain resilience plans. The integration of MPND with financial and employment data can help formulate informed fiscal policies that mitigate the long-term economic impact of health crises.

Fourth, enhancing economic resilience through MPND analysis is crucial for governments and public health authorities. MPND’s application in assessing economic vulnerability offers valuable insights into the pandemic’s impact on businesses. Developing a vulnerability index to monitor and predict economic risks, such as community depopulation and business disruptions, will assist policymakers in anticipating long-term economic trends and ensuring sustainable economic recovery.

Finally, strengthening urban health system resilience through community health workers (CHWs) is essential for improving emergency response strategies. Integrating CHWs into urban planning can minimize the need for extensive travel during health crises, ensuring faster and more localized healthcare delivery. Establishing health stations in densely populated or mobility-limited areas enhances accessibility to essential services, particularly for vulnerable populations.

## Supporting information

S1 Table PRISMA 2020 checklist. (DOCX)

S2 Table Assessment of study quality. (DOCX)

S3 Table List of Excluded Studies. (DOCX)

S4 Table List of Included and Excluded Studies. (DOCX)
