# Supplementary material for: Mobile Phone Network Data in the COVID-19 era: A systematic review of applications, socioeconomic factors affecting compliance to non-pharmaceutical interventions, privacy implications, and post-pandemic economic recovery strategies
Source: PLoS One. 2025 Apr 29;20(4):e0322520. doi: 10.1371/journal.pone.0322520 (PMC12040144; doi:10.1371/journal.pone.0322520)
Supplement: S2 Table — (DOCX) [file pone.0322520.s002.docx]

| **No.** | **Title** | **Quantitative studies**  **(MMAT Tool)** | **1.1: Is the sampling strategy relevant to address the research question?** | **1.2: Is the sample representative of the target population?** | **1.3: Are the measurements appropriate?** | **1.4: Is the risk of nonresponse bias low?** | **1.5: Is the statistical analysis appropriate to answer the research question?** | **Comments** |
| --- | --- | --- | --- | --- | --- | --- | --- | --- |
| 1 | COVID-19 Flow-Maps an open geographic information system on COVID-19 and human mobility for Spain | Ponce-de-Leon [2] | Yes | Yes | Yes | Yes | Yes | - |
| 2 | Countrywide population movement monitoring using mobile devices generated (big) data during the COVID-19 crisis | Szocska [3] | Yes | Yes | Yes | Yes | Yes | - |
| 3 | Population flow drives spatio-temporal distribution of COVID-19 in China | Jia [7] | Yes | Yes | Yes | Yes | Yes | - |
| 4 | Mass Tracking in Cellular Networks for the COVID-19 Pandemic Monitoring | Khatib [8] | Yes | Yes | Yes | Yes | Yes | - |
| 5 | Using mobile phone data to estimate dynamic population changes and improve the understanding of a pandemic: A case study in Andorra | Berke [12] | Yes | Yes | Yes | Yes | Yes | - |
| 6 | Effects of human mobility restrictions on the spread of COVID-19 in Shenzhen, China: a modelling study using mobile phone data | Zhou [13] | Yes | Yes | Yes | Yes | Yes | - |
| 7 | Are All Urban Parks Robust to the COVID-19 Pandemic? Focusing on Type, Functionality, and Accessibility | Sung [15] | Yes | Yes | Yes | Yes | Yes | - |
| 8 | Psychotropic drug purchases during the COVID-19 pandemic in Italy and their relationship with mobility restrictions | Marazzi [16] | Yes | Yes | Yes | Yes | Yes | - |
| 9 | Association between mobility patterns and COVID-19 transmission in the USA: a mathematical modelling study | Badr [28] | Yes | Yes | Yes | Yes | Yes | - |
| 10 | How did human dwelling and working intensity change over different stages of COVID-19 in Beijing? | Liu [29] | Yes | Yes | Yes | No | Yes | Certain changes in human activities were overlooked, and not all activities were thoroughly examined, indicating that the risk of nonresponse bias may not be low. |
| 11 | Analyzing COVID-19’s impact on the travel mobility of various social groups in China’s Greater Bay Area via mobile phone big data | Pan and He [30] | Yes | Yes | No | Yes | Yes | The limitations of the data's accuracy and temporal frequency in terms of an hourly time frame and weekly averages of travel frequency and activity indicate that the measurements used in the study are not fully appropriate for providing a detailed depiction of individuals' travel trajectories. |
| 12 | Changes in tourist mobility after COVID-19 outbreaks | Yu [21] | Yes | No | Yes | Yes | Yes | The study's sample is limited to Beijing city. Specifically, the study's focus on Beijing may not fully capture the diversity of pandemic responses and characteristics in other cities or regions. Therefore, the sample limitation could impact the generalizability of the study's findings to a larger target population with different pandemic responses and characteristics. |
| 13 | Assessing spread risk of COVID-19 in early 2020 | Lai [27] | Yes | Yes | No | Yes | Yes | Limitation to individuals with smartphones using the Baidu app might result in an incomplete and biased representation of travelers. |
| 14 | Associations between changes in population mobility in response to the COVID-19 pandemic and socioeconomic factors at the city level in China and country level worldwide: a retrospective, observational study | Liu [32] | Yes | Yes | Yes | Yes | Yes | - |
| 15 | Evaluating the effect of demographic factors, socioeconomic factors, and risk aversion on mobility during the COVID-19 epidemic in France under lockdown: a population-based study | Pullano [33] | Yes | Yes | Yes | Yes | Yes | - |
| 16 | Political beliefs affect compliance with government mandates | Painter [34] | Yes | Yes | Yes | Yes | Yes | - |
| 17 | Political partisanship and mobility restriction during the COVID-19 pandemic | Hsiehchen [35] | Yes | Yes | Yes | Yes | Yes | - |
| 18 | Country-wide Mobility Changes Observed Using Mobile Phone Data During COVID-19 Pandemic | Heiler [36] | Yes | No | Yes | Yes | Yes | The sample might not be fully representative of the target population. |
| 19 | Mobility in China, 2020: a tale of four phases | Tan [37] | Yes | Yes | Yes | Yes | Yes | - |
| 20 | Association between mobility, non-pharmaceutical interventions, and COVID-19 transmission in Ghana: A modelling study using mobile phone data | Gibbs [38] | Yes | Yes | Yes | Yes | Yes | - |
| 21 | Intracity Pandemic Risk Evaluation Using Mobile Phone Data: The Case of Shanghai during COVID-19 | Gan [39] | Yes | Yes | Yes | Yes | Yes | - |
| 22 | Social connections with COVID-19–affected areas increase compliance with mobility restrictions | Charoenwong [41] | Yes | Yes | Yes | Yes | Yes | - |
| 23 | Using mobile phone big data to discover the spatial patterns of rural migrant workers’ return to work in China’s three urban agglomerations in the post-COVID-19 era | Liu [31] | Yes | No | Yes | Yes | Yes | The study relies on data from a single telecom operator, which may not accurately represent all RMWs. Variations in smartphone usage and migration patterns across demographic groups and regions are not fully captured, impacting the sample's representativeness. |
| 24 | Structural changes in intercity mobility networks of China during the COVID-19 outbreak: A weighted stochastic block modeling analysis | Zhang [42] | Yes | Yes | Yes | Yes | Yes | - |
| 25 | Using mobile phone data for epidemic response in low resource settings—A case study of COVID-19 in Malawi | Green [44] | yes | No | No | yes | yes | The sample may not be representative of the entire population due to variations in mobile phone ownership. Also, the appropriateness of measures might be influenced by fluctuations in data coverage and accuracy. |
| 26 | A Privacy-Preserved and Cost-Efficient Control Scheme for Coronavirus Outbreak Using Call Data Record and Contact Tracing | Nisar [45] | Yes | No | Yes | Yes | No | Lacks details regarding the accuracy and effectiveness of the measurements used to trace and track COVID-19 patients. |
| 27 | A robust tracking system for COVID-19 like pandemic using advanced hybrid technologies | Nisar [46] | Yes | No | Yes | Yes | No | Lacks details regarding the accuracy and effectiveness of the measurements used to trace and track COVID-19 patients. |
| 28 | Escaping from Cities during the COVID-19 Crisis: Using Mobile Phone Data to Trace Mobility in Finland | Willberg [43] | Yes | Yes | Yes | Yes | Yes | - |
| 29 | Genomics, social media and mobile phone data enable mapping of SARS-CoV-2 lineages to inform health policy in Bangladesh | Cowley [47] | Yes | Yes | Yes | Yes | Yes | - |
| 30 | The hidden potential of call detail records in The Gambia | Arai [49] | Yes | No | Yes | Yes | No | The limitations revolve around the sample's coverage and potential over-representation of certain user groups. |
| 31 | The city turned off: Urban dynamics during the COVID-19 pandemic based on mobile phone data | Romanillos [50] | Yes | Yes | Yes | Yes | Yes | - |
| 32 | Impacts of the Covid-19 pandemic in inner areas Remote work and near-home tourism through mobile phone data in Piacenza Apennine | Lanza [25] | Yes | No | Yes | Yes | Yes | Limitations in the representativeness of mobile phone data in certain rural areas and the lack of socio-demographic profiling. |
| 33 | Mobility during the COVID-19 Pandemic: A Data-Driven Time-Geographic Analysis of Health-Induced Mobility Changes | Toger [51] | Yes | No | Yes | Yes | Yes | Limitations in the representativeness of mobile phone data in certain rural areas. |
| 34 | COVID-19’s impact on visitation behavior to US national parks from communities of color: evidence from mobile phone data | Alba [52] | Yes | Yes | Yes | Yes | Yes | - |
| 35 | Analysis of the impact of non-compulsory measures on human mobility in Japan during the COVID-19 pandemic | Wu [53] | Yes | Yes | Yes | Yes | Yes | - |
| 36 | The association between socioeconomic status and mobility reductions in the early stage of England’s COVID-19 epidemic. Health Place | Lee [56] | Yes | Yes | Yes | Yes | Yes | - |
| 37 | Socio-Economic Situation in Latvia’s Municipalities in the Context of Administrative-Territorial Division and Unexpected Impact of COVID-19 | Arhipova [55] | Yes | Yes | Yes | Yes | Yes | - |
| 38 | Evaluating the impact of COVID-19 countermeasures on alcohol consumption through wastewater-based epidemiology: A case study in Belgium | Boogaerts [57] | Yes | Yes | Yes | Yes | Yes | - |
| 39 | Higher risk of death from COVID-19 in low-income and non-White populations of São Paulo, Brazil | Li [58] | Yes | Yes | Yes | Yes | Yes | - |
| 40 | The Social Divide of Social Distancing: Shelter-in-Place Behavior in Santiago During the Covid-19 Pandemic | Carranza [59] | Yes | Yes | Yes | Yes | Yes | - |
| 41 | COVID-19 policy analysis: labour structure dictates lockdown mobility behaviour | Heroy [60] | Yes | Yes | Yes | Yes | Yes | - |
| 42 | Behavioral gender differences are reinforced during the COVID-19 crisis | Reisch [61] | Yes | Yes | Yes | Yes | Yes | - |
| 43 | Estimating the effect of social inequalities on the mitigation of COVID-19 across communities in Santiago de Chile | Gozzi [54] | Yes | No | Yes | Yes | Yes | The absence of demographic details makes it difficult to assess whether the users constitute a representative sample of the overall population. |
| 44 | Novel indicator for the spread of new coronavirus disease 2019 and its association with human mobility in Japan | Kawakami [48] | Yes | No | Yes | Yes | Yes | The reported infection numbers were very small to effectively assess the impact of states of emergency on infection spread and human mobility during the initial stages of the epidemic. |
| 45 | Estimation of mobility and population in Spain during different phases of the COVID-19 pandemic from mobile phone data | [Osorio Arjona](https://www.nature.com/articles/s41598-023-36108-1#auth-Joaqu_n-Osorio_Arjona-Aff1) [40] | Yes | No | Yes | Yes | Yes | Mobile phone usage patterns may not represent the entire population, as certain groups, like children and the elderly, may not be adequately captured. Therefore, the sample may not be fully representative of the entire target population. |
| 46 | Socio-economic determinants of mobility responses during the first wave of COVID-19 in Italy: from provinces to neighbourhoods | Gauvin [9] | Yes | Yes | Yes | Yes | Yes | - |

| **No.** | **Title** | **Qualitative Studies**  **(CASP Tool)** | **2.1: Was there a clear statement of the aim?** | **2.2: Is the research method appropriate for addressing the research question or objectives?** | **2.3: Was the research conducted with rigor and integrity to address the aims of the research?** | **2.4: Have ethical and privacy issues been taken into consideration?** | **2.5: Is there a clear presentation of key insights or takeaways from the study?** |
| --- | --- | --- | --- | --- | --- | --- | --- |
| 1 | Mobile phone data for informing public health actions across the COVID-19 pandemic life cycle | Oliver [5] | Yes | Yes | Yes | Yes | Yes |
| 2 | The use of mobile phone data to inform analysis of COVID-19 pandemic epidemiology | Grantz [6] | Yes | Yes | Yes | Yes | Yes |
| 3 | COVID-19 Mobile Positioning Data Contact Tracing and Patient Privacy Regulations: Exploratory Search of Global Response Strategies and the Use of Digital Tools in Nigeria | Ekong [11] | Yes | Yes | Yes | Yes | Yes |
| 4 | Challenges and opportunities in accessing mobile phone data for COVID-19 response in developing countries | Milusheva [14] | Yes | Yes | No | Yes | Yes |
| 5 | Public health research using cell phone derived mobility data in sub-Saharan Africa: Ethical issues | Rennie [62] | Yes | Yes | No | Yes | Yes |
| 6 | Analysis of call detail records to inform the COVID-19 response in Ghana—opportunities and challenges | Li [19] | Yes | Yes | No | Yes | No |
| 7 | Data sharing and collaborations with Telco data during the COVID-19 pandemic: A Vodafone case study | Lourenco [20] | Yes | Yes | No | Yes | Yes |
| 8 | The ethical dilemma of mobile phone data monitoring during COVID-19: The case for South Korea and the United States | Anom [63] | Yes | Yes | No | Yes | No |
| 9 | Measuring mobility to monitor travel and physical distancing interventions: a common framework for mobile phone data analysis | Kishore [64] | Yes | Yes | No | Yes | Yes |
