# Supplementary material for: Mobile Phone Network Data in the COVID-19 era: A systematic review of applications, socioeconomic factors affecting compliance to non-pharmaceutical interventions, privacy implications, and post-pandemic economic recovery strategies
Source: PLoS One. 2025 Apr 29;20(4):e0322520. doi: 10.1371/journal.pone.0322520 (PMC12040144; doi:10.1371/journal.pone.0322520)
Supplement: S3 Table — (DOCX) [file pone.0322520.s003.docx]

**S3 Table. List of Excluded Studies**

| **No.** | **Title** | **Reason for exclusion** | **Type of mobility data used** |
| --- | --- | --- | --- |
| 1 | Effects of social distancing on the spreading of COVID-19 inferred from mobile phone data | Studies utilizing mobile phone network data not sourced from mobile network providers, such as data from phone applications, GPS, or other app-based platforms. | Apple maps data |
| 2 | Varieties of Mobility Measures: Comparing Survey and Mobile Phone Data during the COVID-19 Pandemic | Studies utilizing mobile phone network data not sourced from mobile network providers, such as data from phone applications, GPS, or other app-based platforms. | GPS location data |
| 3 | Mobile phone GPS data and prevalence of COVID-19 infections: Quantifying parameters of social distancing in the U.S. | Studies utilizing mobile phone network data not sourced from mobile network providers, such as data from phone applications, GPS, or other app-based platforms. | GPS location data |
| 4 | Using crowd-sourced data for real-time monitoring of food prices during the COVID-19 pandemic: Insights from a pilot project in northern Nigeria | Studies exclusively focus on the use of mobile phone applications to control the COVID-19 outbreak. | Mobile phone application data |
| 5 | Measuring the impact of COVID-19 on China's population migration with mobile phone data | Studies are not written in the English language. | N/A |
| 6 | Traffic Analysis Zone-Based Epidemic Estimation Approach of COVID-19 Based on Mobile Phone Data: An Example of Wuhan | Studies are not written in the English language. | N/A |
| 7 | La Continuidad de la Educación Superior en Brasil en Covid-19: Proyecto Estudiantes Conectados | Studies are not written in the English language. | N/A |
| 8 | Ethics of instantaneous contact tracing using mobile phone apps in the control of the COVID-19 pandemic | Studies exclusively focus on the use of mobile phone applications to control the COVID-19 outbreak. | N/A |
| 9 | Where do people meet? Time-series clustering for social interaction levels in daily-life spaces during the COVID-19 pandemic | Studies utilizing mobile phone network data not sourced from mobile network providers, such as data from phone applications, GPS, or other app-based platforms. | Location-based service applications |
| 10 | The effect of population mobility on COVID-19 incidence in 314 Latin American cities: a longitudinal ecological study with mobile phone location data | Studies exclusively focus on the use of mobile phone applications to control the COVID-19 outbreak. | Mobile phone application data |
| 11 | Location-Based Services Using Web-Gis By An Android Platform To Improve Students' Navigation During Covid-19 | Studies exclusively focus on the use of mobile phone applications to control the COVID-19 outbreak. | Location-based service applications |
| 12 | Ten GIS-based solutions for managing and controlling COVID-19 pandemic outbreak | Studies exclusively focus on the use of mobile phone applications to control the COVID-19 outbreak. | Location-based service applications |
| 13 | Rapid Development of Location-based Apps: Saving Lives during a Pandemic–the South Korean Experience | Studies exclusively focus on the use of mobile phone applications to control the COVID-19 outbreak. | Location-based service applications |
| 14 | Location-based games and the covid-19 pandemic: An analysis of responses from game developers and players | Studies exclusively focus on the use of mobile phone applications to control the COVID-19 outbreak. | Location-based service applications |
| 15 | Covid-19 pandemic and activity patterns in Milan. Wi-Fi sensors and location-based data | Studies utilizing mobile phone network data not sourced from mobile network providers, such as data from phone applications, GPS, or other app-based platforms. | Wi-Fi data |
| 16 | The case for small-scale, mobile-enhanced COVID-19 epidemiology | Studies utilizing mobile phone network data not sourced from mobile network providers, such as data from phone applications, GPS, or other app-based platforms. | Wi-Fi data |
| 17 | Using passive Wi-Fi for community crowd sensing during the COVID-19 pandemic. | Studies utilizing mobile phone network data not sourced from mobile network providers, such as data from phone applications, GPS, or other app-based platforms. | Wi-Fi data |
| 18 | Application of semantic location awareness computing based on data mining in COVID-19 prevention and control system | Studies exclusively focus on the use of mobile phone applications to control the COVID-19 outbreak. | location-based service applications |
| 19 | Location-aware systems or location-based services: a survey with applications to CoViD-19 contact tracking | Studies exclusively focus on the use of mobile phone applications to control the COVID-19 outbreak. | location-based service applications |
| 20 | Evaluating Apple Inc mobility trend data related to the COVID-19 outbreak in Japan: Statistical analysis | Studies utilizing mobile phone network data not sourced from mobile network providers, such as data from phone applications, GPS, or other app-based platforms. | Apple maps data |
| 21 | Mining Google and Apple mobility data: Temporal anatomy for COVID-19 social distancing | Studies utilizing mobile phone network data not sourced from mobile network providers, such as data from phone applications, GPS, or other app-based platforms. | Apple maps data |
| 22 | Assessing the Governance of Digital Contact Tracing in Response to COVID-19: Results of a Multi-National Study | Studies exclusively focus on the use of mobile phone applications to control the COVID-19 outbreak. | Mobile phone application data |
| 23 | Visualizing social and behavior change due to the outbreak of COVID-19 using mobile phone location data | Studies utilizing mobile phone network data not sourced from mobile network providers, such as data from phone applications, GPS, or other app-based platforms. | GPS location data |
| 24 | Hotspot analysis of COVID-19 infection using mobile-phone location data | Studies utilizing mobile phone network data not sourced from mobile network providers, such as data from phone applications, GPS, or other app-based platforms. | GPS location data |
| 25 | A mobile health application using Geolocation for behavioral activity tracking | Studies utilizing mobile phone network data not sourced from mobile network providers, such as data from phone applications, GPS, or other app-based platforms | GPS location data |
| 26 | A city cluster risk-based approach for Sars-CoV-2 and isolation barriers based on anonymized mobile phone users’ location data | Studies utilizing mobile phone network data not sourced from mobile network providers, such as data from phone applications, GPS, or other app-based platforms. | Mobile phone application data |
| 27 | On-site dining in Tokyo during the COVID-19 pandemic: Time series analysis using mobile phone location data | Studies utilizing mobile phone network data not sourced from mobile network providers, such as data from phone applications, GPS, or other app-based platforms. | GPS location data |
| 28 | Challenges in contact tracing by mining mobile phone location data for COVID-19: Implications for public governance in South Africa | Studies utilizing mobile phone network data not sourced from mobile network providers, such as data from phone applications, GPS, or other app-based platforms. | GPS location data |
| 29 | Data Management and Privacy Policy of COVID-19 Contact-Tracing Apps: Systematic Review and Content Analysis | Studies utilizing mobile phone network data not sourced from mobile network providers, such as data from phone applications, GPS, or other app-based platforms. | GPS location data |
| 30 | How GPs adapted their practices and organisations at the beginning of COVID-19 outbreak: a French national observational survey | Studies utilizing mobile phone network data not sourced from mobile network providers, such as data from phone applications, GPS, or other app-based platforms. | GPS location data |
| 31 | Impact of COVID-19 pandemic on home range in a suburban city in the Osaka metropolitan area | Studies utilizing mobile phone network data not sourced from mobile network providers, such as data from phone applications, GPS, or other app-based platforms. | GPS location data |
| 32 | Mobile phone data reveals spatiotemporal recreational patterns in conservation areas during the COVID pandemic | Studies utilizing mobile phone network data not sourced from mobile network providers, such as data from phone applications, GPS, or other app-based platforms. | GPS location data |
| 33 | Is travel behaviour an equity issue? Using GPS location data to assess the effects of income and supermarket availability on travel reduction during the COVID-19 pandemic | Studies utilizing mobile phone network data not sourced from mobile network providers, such as data from phone applications, GPS, or other app-based platforms. | GPS location data |
| 34 | Health care visits during the COVID-19 pandemic: A spatial and temporal analysis of mobile device data | Studies exclusively focus on the use of mobile phone applications to control the COVID-19 outbreak. | Mobile phone application data |
| 35 | The corona crisis, data protection and tracking apps in the EU: the Czech and Austrian COVID-19 mobile phone apps in the battle against the virus | Studies exclusively focus on the use of mobile phone applications to control the COVID-19 outbreak. | Mobile phone application data |
| 36 | COVID-19 Contact Tracing: Ghana’s Efforts in the Application of Geospatial Technology in Minimizing the Impact of the Pandemic | Studies exclusively focus on the use of mobile phone applications to control the COVID-19 outbreak. | Mobile phone application data |
| 37 | The practicality of mobile applications in healthcare administration and COVID-19 pandemic | Studies exclusively focus on the use of mobile phone applications to control the COVID-19 outbreak. | Mobile phone application data |
| 38 | Mining user reviews of COVID contact-tracing apps: An exploratory analysis of nine European apps | Studies exclusively focus on the use of mobile phone applications to control the COVID-19 outbreak. | Mobile phone application data |
| 39 | Toward a secure global contact tracing app for COVID-19 | Studies exclusively focus on the use of mobile phone applications to control the COVID-19 outbreak. | Mobile phone application data |
| 40 | Privacy-preserving COVID-19 contact tracing solution based on blockchain | Studies exclusively focus on the use of mobile phone applications to control the COVID-19 outbreak. | Mobile phone application data |
| 41 | COVID-19 Contact Tracing Using Blockchain | Studies exclusively focus on the use of mobile phone applications to control the COVID-19 outbreak. | Mobile phone application data |
| 42 | Contact tracing apps for the COVID-19 pandemic: a systematic literature review of challenges and future directions for neo-liberal societies | Studies exclusively focus on the use of mobile phone applications to control the COVID-19 outbreak. | Mobile phone application data |
| 43 | Effectiveness modelling of digital contact-tracing solutions for tackling the COVID-19 pandemic | Studies exclusively focus on the use of mobile phone applications to control the COVID-19 outbreak. | Mobile phone application data |
| 44 | Estimates of the Carbon Impacts of Commute Travel Restrictions due to COVID-19 in the UK | Studies utilizing mobile phone network data not sourced from mobile network providers, such as data from phone applications, GPS, or other app-based platforms. | N/A |
| 45 | Modeling the effects of contact-tracing apps on the spread of the coronavirus disease: Mechanisms, conditions, and efficiency | Studies exclusively focus on the use of mobile phone applications to control the COVID-19 outbreak. | Mobile phone application data |
| 46 | Effectiveness evaluation of digital contact tracing for COVID-19 in New South Wales, Australia | Studies exclusively focus on the use of mobile phone applications to control the COVID-19 outbreak. | Mobile phone application data |
| 47 | Contact tracing apps for self-quarantine in South Korea: rethinking datafication and dataveillance in the COVID-19 age | Studies exclusively focus on the use of mobile phone applications to control the COVID-19 outbreak. | Mobile phone application data |
| 48 | Investigating the effective factors of using mHealth apps for monitoring COVID-19 symptoms and contact tracing: A survey among Iranian citizens | Studies exclusively focus on the use of mobile phone applications to control the COVID-19 outbreak. | Mobile phone application data |
| 49 | A framework for monitoring movements of pandemic disease patients based on GPS trajectory datasets | Studies utilizing mobile phone network data not sourced from mobile network providers, such as data from phone applications, GPS, or other app-based platforms. | GPS location data |
| 50 | Using GPS-enabled mobile phones to evaluate the associations between human mobility changes and the onset of influenza illness | Studies utilizing mobile phone network data not sourced from mobile network providers, such as data from phone applications, GPS, or other app-based platforms. | GPS location data |
| 51 | Community Mobility and COVID-19 Dynamics in Jakarta, Indonesia | Studies utilizing mobile phone network data not sourced from mobile network providers, such as data from phone applications, GPS, or other app-based platforms. | Google mobility data |
| 52 | Exploring the Utility of Google Mobility Data During the COVID-19 Pandemic in India: Digital Epidemiological Analysis | Studies utilizing mobile phone network data not sourced from mobile network providers, such as data from phone applications, GPS, or other app-based platforms. | Google mobility data |
| 53 | Cell Phone Activity in Categories of Places and Associations With Growth in Cases of COVID-19 in the US | Studies utilizing mobile phone network data not sourced from mobile network providers, such as data from phone applications, GPS, or other app-based platforms. | Google mobility data, GPS location data |
| 54 | COVID-19 is spatial: Ensuring that mobile Big Data is used for social good | Studies utilizing mobile phone network data not sourced from mobile network providers, such as data from phone applications, GPS, or other app-based platforms. | Google mobility data, Apple Mobility data |
| 55 | Twitter reveals human mobility dynamics during the COVID-19 pandemic | Studies utilizing mobile phone network data not sourced from mobile network providers, such as data from phone applications, GPS, or other app-based platforms. | Twitter data |
| 56 | Using mobility data in the design of optimal lockdown strategies for the COVID-19 pandemic | Studies utilizing mobile phone network data not sourced from mobile network providers, such as data from phone applications, GPS, or other app-based platforms. | Google mobility data |
| 57 | Comparative analysis of geolocation information through mobile-devices under different COVID-19 mobility restriction patterns in Spain | Studies utilizing mobile phone network data not sourced from mobile network providers, such as data from phone applications, GPS, or other app-based platforms. | Google mobility data and Facebook |
| 58 | Mobility and dissemination of COVID-19 in Portugal: correlations and estimates from Google’s mobility data | Studies utilizing mobile phone network data not sourced from mobile network providers, such as data from phone applications, GPS, or other app-based platforms. | Google mobility data |
| 59 | Risk mapping for COVID-19 outbreaks in Australia using mobility data | Studies utilizing mobile phone network data not sourced from mobile network providers, such as data from phone applications, GPS, or other app-based platforms. | Facebook mobility data |
| 60 | Urban exodus? Understanding human mobility in Britain during the COVID‐19 pandemic using Meta‐Facebook data | Studies utilizing mobile phone network data not sourced from mobile network providers, such as data from phone applications, GPS, or other app-based platforms. | Facebook mobility data |
| 61 | Cross-border mobility responses to COVID-19 in Europe: new evidence from Facebook data | Studies utilizing mobile phone network data not sourced from mobile network providers, such as data from phone applications, GPS, or other app-based platforms. | Facebook mobility data |
| 62 | Understanding components of mobility during the COVID-19 pandemic | Studies utilizing mobile phone network data not sourced from mobile network providers, such as data from phone applications, GPS, or other app-based platforms. | Facebook mobility data |
| 63 | Mobile apps to fight the COVID-19 crisis | Studies exclusively focus on the use of mobile phone applications to control the COVID-19 outbreak. | Mobile phone application data |
| 64 | Smartphone apps in the COVID-19 pandemic | Studies exclusively focus on the use of mobile phone applications to control the COVID-19 outbreak. | Mobile phone application data |
| 65 | From computational indicators to law into technologies: the Internet of Things, data analytics and encoding in COVID-19 contact-tracing apps | Studies exclusively focus on the use of mobile phone applications to control the COVID-19 outbreak. | Mobile phone application data |
