# Supplementary material for: Mobile Phone Network Data in the COVID-19 era: A systematic review of applications, socioeconomic factors affecting compliance to non-pharmaceutical interventions, privacy implications, and post-pandemic economic recovery strategies
Source: PLoS One. 2025 Apr 29;20(4):e0322520. doi: 10.1371/journal.pone.0322520 (PMC12040144; doi:10.1371/journal.pone.0322520)
Supplement: S4 Table — (DOCX) [file pone.0322520.s004.docx]

**S4 Table. List of Included and Excluded Studies**

| **No.** | **Article Title** | **Publication Year** | **DOI** | **Source** | **Publisher** | **MIncluded in SLR (meet all inclusion criteria)** | **Reason (if not included in SLR)** |
| --- | --- | --- | --- | --- | --- | --- | --- |
| 1 | Mobile phone data for informing public health actions across the COVID-19 pandemic life cycle | 2020 | 10.1126/sciadv.abc0764 | Scopus | American Association for the Advancement of Science | Yes | N/A |
| 2 | The Care Burden during COVID-19: A National Database of Child Care Closures in the United States | 2021 | 10.1177/23780231211032028 | Scopus | SAGE Publications Inc. | No | Excluded during title and abstract screening for irrelevance |
| 3 | Countrywide population movement monitoring using mobile devices generated (big) data during the COVID-19 crisis | 2021 | 10.1038/s41598-021-81873-6 | Scopus | Scientific Reports | Yes | N/A |
| 4 | Mobile phone data for informing public health actions across the COVID-19 pandemic life cycle | 2020 | 10.1126/sciadv.abc0764 | Web of Science | American Association for the Advancement of Science | No | Duplicate study |
| 5 | Evaluating the impact of COVID-19 countermeasures on alcohol consumption through wastewater-based epidemiology: A case study in Belgium | 2022 | 10.1016/j.envint.2022.107559 | PubMed | Elsevier Ltd | Yes | N/A |
| 6 | Population flow drives spatio-temporal distribution of COVID-19 in China | 2020 | 10.1038/s41586-020-2284-y | Scopus | Nature | Yes | N/A |
| 7 | The Care Burden during COVID-19: A National Database of Child Care Closures in the United States | 2021 | 10.1177/23780231211032028 | Web of Science | SAGE Publications Inc. | No | Duplicate study |
| 8 | Mobile phone data for informing public health actions across the COVID-19 pandemic life cycle | 2020 | 10.1126/sciadv.abc0764 | PubMed | American Association for the Advancement of Science | No | Duplicate study |
| 9 | COVID-19 Flow-Maps an open geographic information system on COVID-19 and human mobility for Spain | 2021 | 10.1038/s41597-021-01093-5 | PubMed | Scientific Data | Yes | N/A |
| 10 | Evaluating the impact of COVID-19 countermeasures on alcohol consumption through wastewater-based epidemiology: A case study in Belgium | 2022 | 10.1016/j.envint.2022.107559 | Scopus | Elsevier Ltd | No | Duplicate study |
| 11 | Evaluating the impact of COVID-19 countermeasures on alcohol consumption through wastewater-based epidemiology: A case study in Belgium | 2022 | 10.1016/j.envint.2022.107559 | Web of Science | Elsevier Ltd | No | Duplicate study |
| 12 | COVID-19 Flow-Maps an open geographic information system on COVID-19 and human mobility for Spain | 2021 | 10.1038/s41597-021-01093-5 | Scopus | Nature | No | Duplicate study |
| 13 | COVID-19 Flow-Maps an open geographic information system on COVID-19 and human mobility for Spain | 2021 | 10.1038/s41597-021-01093-5 | Web of Science | Nature | No | Duplicate study |
| 14 | Population flow drives spatio-temporal distribution of COVID-19 in China | 2020 | 10.1038/s41586-020-2284-y | PubMed | Nature | No | Duplicate study |
| 15 | Population flow drives spatio-temporal distribution of COVID-19 in China | 2020 | 10.1038/s41586-020-2284-y | Web of Science | Nature | No | Duplicate study |
| 16 | Mass Tracking in Cellular Networks for the COVID-19 Pandemic Monitoring | 2021 | 10.3390/s21103424 | Web of Science | MDPI | Yes | N/A |
| 17 | Effects of social distancing on the spreading of COVID-19 inferred from mobile phone data | 2021 | 10.1038/s41598-021-81308-2 | PubMed | Nature | No | Meet one or more of the exclusion criteria |
| 18 | Effects of social distancing on the spreading of COVID-19 inferred from mobile phone data | 2021 | 10.1038/s41598-021-81308-2 | Web of Science | Nature | No | Duplicate study |
| 19 | Effects of social distancing on the spreading of COVID-19 inferred from mobile phone data | 2021 | 10.1038/s41598-021-81308-2 | Scopus | Nature | No | Duplicate study |
| 20 | Varieties of Mobility Measures: Comparing Survey and Mobile Phone Data during the COVID-19 Pandemic | 2022 | 10.1093/poq/nfac042 | Web of Science | Oxford University Press | No | Meet one or more of the exclusion criteria |
| 21 | Varieties of Mobility Measures: Comparing Survey and Mobile Phone Data during the COVID-19 Pandemic | 2022 | 10.1093/poq/nfac042 | Scopus | Oxford University Press | No | Duplicate study |
| 22 | Using mobile phone data to estimate dynamic population changes and improve the understanding of a pandemic: A case study in Andorra | 2022 | 10.1371/journal.pone.0264860 | Scopus | Public Library of Science | Yes | N/A |
| 23 | Using mobile phone data to estimate dynamic population changes and improve the understanding of a pandemic: A case study in Andorra | 2022 | 10.1371/journal.pone.0264860 | Web of Science | Public Library of Science | No | Duplicate study |
| 24 | Using mobile phone data to estimate dynamic population changes and improve the understanding of a pandemic: A case study in Andorra | 2022 | 10.1371/journal.pone.0264860 | PubMed | Public Library of Science | No | Duplicate study |
| 25 | Effects of human mobility restrictions on the spread of COVID-19 in Shenzhen, China: a modelling study using mobile phone data | 2020 | 10.1016/S25897500(20)30165-5 | Scopus | Elsevier Ltd | Yes | N/A |
| 26 | Are All Urban Parks Robust to the COVID-19 Pandemic? Focusing on Type, Functionality, and Accessibility | 2022 | 10.3390/ijerph19106062 | Scopus | MDPI | Yes | N/A |
| 27 | Psychotropic drug purchases during the COVID-19 pandemic in Italy and their relationship with mobility restrictions | 2022 | 10.1038/s41598-022-22085-4 | Web of Science | Nature | Yes | N/A |
| 28 | Association between mobility patterns and COVID-19 transmission in the USA: a mathematical modelling study | 2020 | 10.1016/S14733099(20)30553-3 | PubMed | Lancet Publishing Group | Yes | N/A |
| 29 | How did human dwelling and working intensity change over different stages of COVID-19 in Beijing? | 2021 | 10.1016/j.scs.2021.103206 | Scopus | Elsevier Ltd | Yes | N/A |
| 30 | Analyzing COVID-19’s impact on the travel mobility of various social groups in China’s Greater Bay Area via mobile phone big data | 2022 | 10.1016/j.tra.2022.03.015 | Scopus | Elsevier Ltd | Yes | N/A |
| 31 | Changes in tourist mobility after COVID-19 outbreaks | 2023 | 10.1016/j.annals.2022.103522 | Scopus | Elsevier Ltd | Yes | N/A |
| 32 | Assessing spread risk of COVID-19 in early 2020 | 2022 | [10.1016/j.dsm.2022.08.004](https://doi.org/10.1016/j.dsm.2022.08.004) | Scopus | Elsevier Ltd | Yes | N/A |
| 33 | Associations between changes in population mobility in response to the COVID-19 pandemic and socioeconomic factors at the city level in China and country level worldwide: a retrospective, observational study | 2021 | 10.1016/S25897500(21)00059-5. | PubMed | Lancet Publishing Group | Yes | N/A |
| 34 | Evaluating the effect of demographic factors, socioeconomic factors, and risk aversion on mobility during the COVID-19 epidemic in France under lockdown: a population-based study | 2020 | 10.1016/S25897500(20)30243-0 | PubMed | Lancet Publishing Group | Yes | N/A |
| 35 | Effects of human mobility restrictions on the spread of COVID-19 in Shenzhen, China: a modelling study using mobile phone data | 2020 | 10.1016/S25897500(20)30165-5 | Web of Science | Elsevier Ltd | No | Duplicate study |
| 36 | Effects of human mobility restrictions on the spread of COVID-19 in Shenzhen, China: a modelling study using mobile phone data | 2020 | 10.1016/S25897500(20)30165-5 | PubMed | Elsevier Ltd | No | Duplicate study |
| 37 | Are All Urban Parks Robust to the COVID-19 Pandemic? Focusing on Type, Functionality, and Accessibility | 2022 | 10.3390/ijerph19106062 | Web of Science | MDPI | No | Duplicate study |
| 38 | Are All Urban Parks Robust to the COVID-19 Pandemic? Focusing on Type, Functionality, and Accessibility | 2022 | 10.3390/ijerph19106062 | PubMed | MDPI | No | Duplicate study |
| 39 | Psychotropic drug purchases during the COVID-19 pandemic in Italy and their relationship with mobility restrictions | 2022 | 10.1038/s41598-022-22085-4 | Scopus | Nature | No | Duplicate study |
| 40 | Psychotropic drug purchases during the COVID-19 pandemic in Italy and their relationship with mobility restrictions | 2022 | 10.1038/s41598-022-22085-4 | PubMed | Nature | No | Duplicate study |
| 41 | Association between mobility patterns and COVID-19 transmission in the USA: a mathematical modelling study | 2020 | 10.1016/S14733099(20)30553-3 | Scopus | Lancet Publishing Group | No | Duplicate study |
| 42 | Association between mobility patterns and COVID-19 transmission in the USA: a mathematical modelling study | 2020 | 10.1016/S14733099(20)30553-3 | Web of Science | Lancet Publishing Group | No | Duplicate study |
| 43 | How did human dwelling and working intensity change over different stages of COVID-19 in Beijing? | 2021 | 10.1016/j.scs.2021.103206 | PubMed | Elsevier Ltd | No | Duplicate study |
| 44 | How did human dwelling and working intensity change over different stages of COVID-19 in Beijing? | 2021 | 10.1016/j.scs.2021.103206 | Web of Science | Elsevier Ltd | No | Duplicate study |
| 45 | Analyzing COVID-19’s impact on the travel mobility of various social groups in China’s Greater Bay Area via mobile phone big data | 2022 | 10.1016/j.tra.2022.03.015 | PubMed | Elsevier Ltd | No | Duplicate study |
| 46 | Changes in tourist mobility after COVID-19 outbreaks | 2023 | 10.1016/j.annals.2022.103522 | Web of Science | Elsevier Ltd | No | Duplicate study |
| 47 | Changes in tourist mobility after COVID-19 outbreaks | 2023 | 10.1016/j.annals.2022.103522 | PubMed | Elsevier Ltd | No | Duplicate study |
| 48 | Evaluating the effect of demographic factors, socioeconomic factors, and risk aversion on mobility during the COVID-19 epidemic in France under lockdown: a population-based study | 2020 | 10.1016/S25897500(20)30243-0 | Scopus | Lancet Publishing Group | No | Duplicate study |
| 49 | Evaluating the effect of demographic factors, socioeconomic factors, and risk aversion on mobility during the COVID-19 epidemic in France under lockdown: a population-based study | 2020 | 10.1016/S25897500(20)30243-0 | Web of Science | Lancet Publishing Group | No | Duplicate study |
| 50 | Political beliefs affect compliance with government mandates | 2021 | [10.1016/j.jebo.2021.03.019](https://doi.org/10.1016/j.jebo.2021.03.019) | Scopus | Elsevier Ltd | Yes | N/A |
| 51 | Political partisanship and mobility restriction during the COVID-19 pandemic | 2020 | 10.1016/j.puhe.2020.08.009 | Scopus | Elsevier Ltd | Yes | N/A |
| 52 | Country-wide Mobility Changes Observed Using Mobile Phone Data During COVID-19 Pandemic | 2020 | 10.1109/BigData50022.2020.9378374 | Web of Science | IEEE | Yes | N/A |
| 53 | Mobility in China, 2020: a tale of four phases | 2020 | 10.1093/nsr/nwab148 | Web of Science | Oxford University Press | Yes | N/A |
| 54 | Association between mobility, non-pharmaceutical interventions, and COVID-19 transmission in Ghana: A modelling study using mobile phone data | 2022 | 10.1371/journal.pgph.0000502 | PubMed | Public Library of Science | Yes | N/A |
| 55 | Intracity Pandemic Risk Evaluation Using Mobile Phone Data: The Case of Shanghai during COVID-19 | 2020 | 10.3390/ijgi9120715 | Scopus | MDPI | Yes | N/A |
| 56 | Social connections with COVID-19–affected areas increase compliance with mobility restrictions | 2020 | [10.1126/sciadv.abc3054](https://doi.org/10.1126/sciadv.abc3054) | PubMed | Science | Yes | N/A |
| 57 | Using mobile phone big data to discover the spatial patterns of rural migrant workers’ return to work in China’s three urban agglomerations in the post-COVID-19 era | 2020 | [10.1177/23998083211069375](https://doi.org/10.1177/23998083211069375) | PubMed | SAGE Publications Inc. | Yes | N/A |
| 58 | Structural changes in intercity mobility networks of China during the COVID-19 outbreak: A weighted stochastic block modeling analysis | 2022 | [10.1016/j.compenvurbsys.2022.101846](https://doi.org/10.1016/j.compenvurbsys.2022.101846) | Scopus | Elsevier Ltd | Yes | N/A |
| 59 | Using mobile phone data for epidemic response in low resource settings—A case study of COVID-19 in Malawi | 2021 | 10.1017/dap.2021.14 | Scopus | Cambridge University Press | Yes | N/A |
| 60 | A Privacy-Preserved and Cost-Efficient Control Scheme for Coronavirus Outbreak Using Call Data Record and Contact Tracing | 2021 | 10.1109/MCE.2020.3038023 | Scopus | IEEE | Yes | N/A |
| 61 | A robust tracking system for COVID-19 like pandemic using advanced hybrid technologies | 2023 | 10.1007/s00607-021-00946-6 | Scopus | IEEE | Yes | N/A |
| 62 | Escaping from Cities during the COVID-19 Crisis: Using Mobile Phone Data to Trace Mobility in Finland | 2021 | 10.3390/ijgi10020103 | Web of Science | MDPI | Yes | N/A |
| 63 | Genomics, social media and mobile phone data enable mapping of SARS-CoV-2 lineages to inform health policy in Bangladesh | 2021 | 10.1038/s41564-021-00955-3 | PubMed | Nature | Yes | N/A |
| 64 | The hidden potential of call detail records in The Gambia | 2021 | 10.1017/dap.2021.7 | Scopus | Cambridge University Press | Yes | N/A |
| 65 | The city turned off: Urban dynamics during the COVID-19 pandemic based on mobile phone data | 2021 | 10.1016/j.apgeog.2021.102524 | Scopus | Elsevier Ltd | Yes | N/A |
| 66 | Impacts of the Covid-19 pandemic in inner areas Remote work and near-home tourism through mobile phone data in Piacenza Apennine | 2022 | [10.6093/1970-9870/8915](https://doi.org/10.6093/1970-9870/8915) | Scopus | TeMA | Yes | N/A |
| 67 | Political partisanship and mobility restriction during the COVID-19 pandemic | 2020 | 10.1016/j.puhe.2020.08.009 | Web of Science | Elsevier Ltd | No | Duplicate study |
| 68 | Political partisanship and mobility restriction during the COVID-19 pandemic | 2020 | 10.1016/j.puhe.2020.08.009 | PubMed | Elsevier Ltd | No | Duplicate study |
| 69 | Country-wide Mobility Changes Observed Using Mobile Phone Data During COVID-19 Pandemic | 2020 | 10.1109/BigData50022.2020.9378374 | Scopus | IEEE | No | Duplicate study |
| 70 | Mobility in China, 2020: a tale of four phases | 2020 | 10.1093/nsr/nwab148 | PubMed | Oxford University Press | No | Duplicate study |
| 71 | Mobility in China, 2020: a tale of four phases | 2020 | 10.1093/nsr/nwab148 | Scopus | Oxford University Press | No | Duplicate study |
| 72 | Association between mobility, non-pharmaceutical interventions, and COVID-19 transmission in Ghana: A modelling study using mobile phone data | 2022 | 10.1371/journal.pgph.0000502 | Web of Science | Public Library of Science | No | Duplicate study |
| 73 | Association between mobility, non-pharmaceutical interventions, and COVID-19 transmission in Ghana: A modelling study using mobile phone data | 2022 | 10.1371/journal.pgph.0000502 | Scopus | Public Library of Science | No | Duplicate study |
| 74 | Escaping from Cities during the COVID-19 Crisis: Using Mobile Phone Data to Trace Mobility in Finland | 2021 | 10.3390/ijgi10020103 | Scopus | MDPI | No | Duplicate study |
| 75 | Genomics, social media and mobile phone data enable mapping of SARS-CoV-2 lineages to inform health policy in Bangladesh | 2021 | 10.1038/s41564-021-00955-3 | Web of Science | Nature | No | Duplicate study |
| 76 | Genomics, social media and mobile phone data enable mapping of SARS-CoV-2 lineages to inform health policy in Bangladesh | 2021 | 10.1038/s41564-021-00955-3 | Scopus | Nature | No | Duplicate study |
| 77 | The city turned off: Urban dynamics during the COVID-19 pandemic based on mobile phone data | 2021 | 10.1016/j.apgeog.2021.102524 | Web of Science | Elsevier Ltd | No | Duplicate study |
| 78 | The city turned off: Urban dynamics during the COVID-19 pandemic based on mobile phone data | 2021 | 10.1016/j.apgeog.2021.102524 | PubMed | Elsevier Ltd | No | Duplicate study |
| 79 | Mobility during the COVID-19 Pandemic: A Data-Driven Time-Geographic Analysis of Health-Induced Mobility Changes | 2021 | 10.3390/su13074027 | Scopus | MDPI | Yes | N/A |
| 80 | COVID-19’s impact on visitation behavior to US national parks from communities of color: evidence from mobile phone data | 2022 | 10.1038/s41598-022-16330-z | PubMed | Nature | Yes | N/A |
| 81 | Analysis of the impact of non-compulsory measures on human mobility in Japan during the COVID-19 pandemic | 2022 | 10.1016/j.cities.2022.103751 | Web of Science | Elsevier Ltd | Yes | N/A |
| 82 | The association between socioeconomic status and mobility reductions in the early stage of England’s COVID-19 epidemic. Health Place | 2021 | 10.1016/j.healthplace.2021.102563 | Web of Science | Elsevier Ltd | Yes | N/A |
| 83 | Socio-Economic Situation in Latvia’s Municipalities in the Context of Administrative-Territorial Division and Unexpected Impact of COVID-19 | 2022 | 10.4018/JGIM.298002 | Scopus | IGI Global | Yes | N/A |
| 84 | The use of mobile phone data to inform analysis of COVID | 2020 | 10.1038/s41467-020-18190-5 | PubMed | Nature | Yes | N/A |
| 85 | Higher risk of death from COVID-19 in low-income and non-White populations of São Paulo, Brazil | 2021 | 10.1136/bmjgh-2021-004959 | Scopus | BMJ Publishing Group | Yes | N/A |
| 86 | The Social Divide of Social Distancing: Shelter-in-Place Behavior in Santiago During the Covid-19 Pandemic | 2022 | 10.1287/mnsc.2021.4240 | Scopus | INFORMS | Yes | N/A |
| 87 | COVID-19 policy analysis: labour structure dictates lockdown mobility behaviour | 2021 | 10.1098/rsif.2020.1035 | Web of Science | Royal Society Publishing | Yes | N/A |
| 88 | Behavioral gender differences are reinforced during the COVID-19 crisis | 2021 | 10.1038/s41598-021-97394-1 | PubMed | Nature | Yes | N/A |
| 89 | Estimating the effect of social inequalities on the mitigation of COVID-19 across communities in Santiago de Chile | 2021 | 10.1038/s41467-021-22601-6 | PubMed | Nature | Yes | N/A |
| 90 | Novel indicator for the spread of new coronavirus disease 2019 and its association with human mobility in Japan | 2023 | 10.1038/s41598-022-27322-4 | PubMed | Nature | Yes | N/A |
| 91 | Estimation of mobility and population in Spain during different phases of the COVID-19 pandemic from mobile phone data | 2023 | 10.1038/s41598-023-36108-1 | PubMed | Nature | Yes | N/A |
| 92 | Socio-economic determinants of mobility responses during the first wave of COVID-19 in Italy: from provinces to neighbourhoods | 2021 | 10.1098/rsif.2021.0092 | Web of Science | Royal Society Publishing | Yes | N/A |
| 93 | COVID-19’s impact on visitation behavior to US national parks from communities of color: evidence from mobile phone data | 2022 | 10.1038/s41598-022-16330-z | Scopus | Nature | No | Duplicate study |
| 94 | COVID-19’s impact on visitation behavior to US national parks from communities of color: evidence from mobile phone data | 2022 | 10.1038/s41598-022-16330-z | Web of Science | Nature | No | Duplicate study |
| 95 | Analysis of the impact of non-compulsory measures on human mobility in Japan during the COVID-19 pandemic | 2022 | 10.1016/j.cities.2022.103751 | Scopus | Elsevier Ltd | No | Duplicate study |
| 96 | Analysis of the impact of non-compulsory measures on human mobility in Japan during the COVID-19 pandemic | 2022 | 10.1016/j.cities.2022.103751 | PubMed | Elsevier Ltd | No | Duplicate study |
| 97 | The association between socioeconomic status and mobility reductions in the early stage of England’s COVID-19 epidemic. Health Place | 2021 | 10.1016/j.healthplace.2021.102563 | Scopus | Elsevier Ltd | No | Duplicate study |
| 98 | The association between socioeconomic status and mobility reductions in the early stage of England’s COVID-19 epidemic. Health Place | 2021 | 10.1016/j.healthplace.2021.102563 | PubMed | Elsevier Ltd | No | Duplicate study |
| 99 | The use of mobile phone data to inform analysis of COVID | 2020 | 10.1038/s41467-020-18190-5 | Web of Science | Nature | No | Duplicate study |
| 100 | The use of mobile phone data to inform analysis of COVID | 2020 | 10.1038/s41467-020-18190-5 | Scopus | Nature | No | Duplicate study |
| 101 | Higher risk of death from COVID-19 in low-income and non-White populations of São Paulo, Brazil | 2021 | 10.1136/bmjgh-2021-004959 | PubMed | BMJ Publishing Group | No | Duplicate study |
| 102 | Higher risk of death from COVID-19 in low-income and non-White populations of São Paulo, Brazil | 2021 | 10.1136/bmjgh-2021-004959 | Web of Science | BMJ Publishing Group | No | Duplicate study |
| 103 | The Social Divide of Social Distancing: Shelter-in-Place Behavior in Santiago During the Covid-19 Pandemic | 2022 | 10.1287/mnsc.2021.4240 | Web of Science | INFORMS | No | Duplicate study |
| 104 | COVID-19 policy analysis: labour structure dictates lockdown mobility behaviour | 2021 | 10.1098/rsif.2020.1035 | Scopus | Royal Society Publishing | No | Duplicate study |
| 105 | COVID-19 policy analysis: labour structure dictates lockdown mobility behaviour | 2021 | 10.1098/rsif.2020.1035 | PubMed | Royal Society Publishing | No | Duplicate study |
| 106 | Behavioral gender differences are reinforced during the COVID-19 crisis | 2021 | 10.1038/s41598-021-97394-1 | Web of Science | Nature | No | Duplicate study |
| 107 | Behavioral gender differences are reinforced during the COVID-19 crisis | 2021 | 10.1038/s41598-021-97394-1 | Scopus | Nature | No | Duplicate study |
| 108 | Estimating the effect of social inequalities on the mitigation of COVID-19 across communities in Santiago de Chile | 2021 | 10.1038/s41467-021-22601-6 | Web of Science | Nature | No | Duplicate study |
| 109 | Estimating the effect of social inequalities on the mitigation of COVID-19 across communities in Santiago de Chile | 2021 | 10.1038/s41467-021-22601-6 | Scopus | Nature | No | Duplicate study |
| 110 | Novel indicator for the spread of new coronavirus disease 2019 and its association with human mobility in Japan | 2023 | 10.1038/s41598-022-27322-4 | Web of Science | Nature | No | Duplicate study |
| 111 | Novel indicator for the spread of new coronavirus disease 2019 and its association with human mobility in Japan | 2023 | 10.1038/s41598-022-27322-4 | Scopus | Nature | No | Duplicate study |
| 112 | Estimation of mobility and population in Spain during different phases of the COVID-19 pandemic from mobile phone data | 2023 | 10.1038/s41598-023-36108-1 | Web of Science | Nature | No | Duplicate study |
| 113 | Estimation of mobility and population in Spain during different phases of the COVID-19 pandemic from mobile phone data | 2023 | 10.1038/s41598-023-36108-1 | Scopus | Nature | No | Duplicate study |
| 114 | Socio-economic determinants of mobility responses during the first wave of COVID-19 in Italy: from provinces to neighbourhoods | 2021 | 10.1098/rsif.2021.0092 | Scopus | Royal Society Publishing | No | Duplicate study |
| 115 | Socio-economic determinants of mobility responses during the first wave of COVID-19 in Italy: from provinces to neighbourhoods | 2021 | 10.1098/rsif.2021.0092 | PubMed | Royal Society Publishing | No | Duplicate study |
| 116 | COVID-19 Mobile Positioning Data Contact Tracing and Patient Privacy Regulations: Exploratory Search of Global Response Strategies and the Use of Digital Tools in Nigeria | 2020 | 10.2196/19139 | Scopus | JMIR Publications Inc | Yes | N/A |
| 117 | Challenges and opportunities in accessing mobile phone data for COVID-19 response in developing countries | 2021 | 10.1017/dap.2021.10 | Scopus | Cambridge University Press | Yes | N/A |
| 118 | Public health research using cell phone derived mobility data in sub-Saharan Africa: Ethical issues | 2023 | 10.17159/sajs.2023/14777 | Scopus | African journals | Yes | N/A |
| 119 | Analysis of call detail records to inform the COVID-19 response in Ghana—opportunities and challenges | 2021 | [10.1017/dap.2021.5](https://doi.org/10.1017/dap.2021.5) | Scopus | Cambridge University Press | Yes | N/A |
| 120 | Data sharing and collaborations with Telco data during the COVID-19 pandemic: A Vodafone case study | 2021 | [10.1017/dap.2021.26](https://doi.org/10.1017/dap.2021.26) | Scopus | Cambridge University Press | Yes | N/A |
| 121 | The ethical dilemma of mobile phone data monitoring during COVID-19: The case for South Korea and the United States | 2022 | 10.1177/22799036221102491 | Scopus | SAGE | Yes | N/A |
| 122 | Measuring mobility to monitor travel and physical distancing interventions: a common framework for mobile phone data analysis | 2020 | 10.1016/S2589-7500(20)30193-X | Scopus | Elsevier Ltd | Yes | N/A |
| 123 | Measuring mobility to monitor travel and physical distancing interventions: a common framework for mobile phone data analysis | 2020 | 10.1016/S2589-7500(20)30193-X | PubMed | Elsevier Ltd | No | Duplicate study |
| 124 | Measuring mobility to monitor travel and physical distancing interventions: a common framework for mobile phone data analysis | 2020 | 10.1016/S2589-7500(20)30193-X | Web of Science | Elsevier Ltd | No | Duplicate study |
| 125 | Mobile phone GPS data and prevalence of COVID-19 infections: Quantifying parameters of social distancing in the U.S. | 2021 | 10.22038/ABJS.2020.48515.2404 | Scopus | Mashhad University of Medical Sciences | No | Meet one or more of the exclusion criteria |
| 126 | Using crowd-sourced data for real-time monitoring of food prices during the COVID-19 pandemic: Insights from a pilot project in northern Nigeria | 2021 | 10.1016/j.gfs.2021.100523 | Scopus | Elsevier Ltd | No | Meet one or more of the exclusion criteria |
| 127 | Measuring the impact of COVID-19 on China's population migration with mobile phone data | 2021 | 10.7498/aps.70.20202084 | Web of Science | Institute of Physics, Chinese Academy of Sciences | No | Meet one or more of the exclusion criteria |
| 128 | Traffic Analysis Zone-Based Epidemic Estimation Approach of COVID-19 Based on Mobile Phone Data: An Example of Wuhan | 2020 | 10.13203/j.whugis20200141 | Scopus | Wuhan University | No | Meet one or more of the exclusion criteria |
| 129 | La Continuidad de la Educación Superior en Brasil en Covid-19: Proyecto Estudiantes Conectados | 2022 | N/A | Scopus | AEIPRO | No | Meet one or more of the exclusion criteria |
| 130 | Ethics of instantaneous contact tracing using mobile phone apps in the control of the COVID-19 pandemic | 2020 | 10.1136/medethics-2020-106314 | PubMed | BMJ Publishing Group | No | Meet one or more of the exclusion criteria |
| 131 | Where do people meet? Time-series clustering for social interaction levels in daily-life spaces during the COVID-19 pandemic | 2023 | 10.1016/j.cities.2023.104298 | Web of Science | Elsevier Ltd | No | Meet one or more of the exclusion criteria |
| 132 | The effect of population mobility on COVID-19 incidence in 314 Latin American cities: a longitudinal ecological study with mobile phone location data | 2021 | 10.1016/S2589-7500(21)00174-6 | Web of Science | Elsevier Ltd | No | Meet one or more of the exclusion criteria |
| 133 | Location-Based Services Using Web-Gis By An Android Platform To Improve Students' Navigation During Covid-19 | 2022 | N/A | Scopus | [Little Lion Scientific](https://www.scimagojr.com/journalsearch.php?q=Little%20Lion%20Scientific&tip=pub) | No | Meet one or more of the exclusion criteria |
| 134 | Ten GIS-based solutions for managing and controlling COVID-19 pandemic outbreak | 2022 | 10.1007/s42979-022-01150-9 | Scopus | Springer | No | Meet one or more of the exclusion criteria |
| 135 | Rapid Development of Location-based Apps: Saving Lives during a Pandemic–the South Korean Experience | 2021 | [10.1201/9781003181590-27](http://dx.doi.org/10.1201/9781003181590-27) | Scopus | Taylor and Francis Ltd | No | Meet one or more of the exclusion criteria |
| 136 | Location-based games and the covid-19 pandemic: An analysis of responses from game developers and players | 2020 | [10.3390/mti4020029](https://doi.org/10.3390/mti4020029) | Web of Science | MDPI | No | Meet one or more of the exclusion criteria |
| 137 | Covid-19 pandemic and activity patterns in Milan. Wi-Fi sensors and location-based data | 2021 | [10.6093/1970-9870/7886](https://doi.org/10.6093/1970-9870/7886) | Scopus | TeMA | No | Meet one or more of the exclusion criteria |
| 138 | The case for small-scale, mobile-enhanced COVID-19 epidemiology | 2021 | [10.23919/WiOpt52861.2021.9589290](https://doi.org/10.23919/WiOpt52861.2021.9589290) | Web of Science | IEEE | No | Meet one or more of the exclusion criteria |
| 139 | Using passive Wi-Fi for community crowd sensing during the COVID-19 pandemic. | 2023 | 10.1186/s40537-022-00675-3 | PubMed | Springer | No | Meet one or more of the exclusion criteria |
| 140 | Application of semantic location awareness computing based on data mining in COVID-19 prevention and control system | 2020 | 10.3233/JIFS-189295 | PubMed | IOS Press | No | Meet one or more of the exclusion criteria |
| 141 | Location-aware systems or location-based services: a survey with applications to CoViD-19 contact tracking | 2020 | 10.1007/s40860-020-00111-4 | PubMed | Springer | No | Meet one or more of the exclusion criteria |
| 142 | Evaluating Apple Inc mobility trend data related to the COVID-19 outbreak in Japan: Statistical analysis | 2020 | [10.2196/20335](https://doi.org/10.2196/20335) | PubMed | JMIR Publications Inc | No | Meet one or more of the exclusion criteria |
| 143 | Mining Google and Apple mobility data: Temporal anatomy for COVID-19 social distancing | 2021 | 10.1038/s41598-021-83441-4 | PubMed | Nature | No | Meet one or more of the exclusion criteria |
| 144 | Assessing the Governance of Digital Contact Tracing in Response to COVID-19: Results of a Multi-National Study | 2022 | [10.1017/jme.2023.20](https://doi.org/10.1017/jme.2023.20) | PubMed | Cambridge University Press | No | Meet one or more of the exclusion criteria |
| 145 | Visualizing social and behavior change due to the outbreak of COVID-19 using mobile phone location data | 2021 | 10.1007/s00354-021-00139-x | Web of Science | Springer | No | Meet one or more of the exclusion criteria |
| 146 | Hotspot analysis of COVID-19 infection using mobile-phone location data | 2022 | 10.1007/s10015-022-00830-2 | PubMed | Springer | No | Meet one or more of the exclusion criteria |
| 147 | A mobile health application using Geolocation for behavioral activity tracking | 2023 | [10.3390/s23187917](https://doi.org/10.3390/s23187917) | PubMed | MDPI | No | Meet one or more of the exclusion criteria |
| 148 | A city cluster risk-based approach for Sars-CoV-2 and isolation barriers based on anonymized mobile phone users’ location data | 2021 | [10.1016/j.scs.2020.102574](https://doi.org/10.1016/j.scs.2020.102574) | PubMed | Elsevier Ltd | No | Meet one or more of the exclusion criteria |
| 149 | On-site dining in Tokyo during the COVID-19 pandemic: Time series analysis using mobile phone location data | 2021 | [10.2196/27342](https://doi.org/10.2196/27342) | PubMed | JMIR Publications Inc | No | Meet one or more of the exclusion criteria |
| 150 | Challenges in contact tracing by mining mobile phone location data for COVID-19: Implications for public governance in South Africa |  | [10.28945/4736](http://dx.doi.org/10.28945/4736) | Web of Science | Information Sciences Institute | No | Meet one or more of the exclusion criteria |
| 151 | Data Management and Privacy Policy of COVID-19 Contact-Tracing Apps: Systematic Review and Content Analysis | 2022 | [10.2196/35195](https://doi.org/10.2196/35195) | PubMed | JMIR Publications Inc | No | Meet one or more of the exclusion criteria |
| 152 | How GPs adapted their practices and organisations at the beginning of COVID-19 outbreak: a French national observational survey | 2020 | [10.1136/bmjopen-2020-042119](https://doi.org/10.1136/bmjopen-2020-042119) | PubMed | BMJ Publishing Group | No | Meet one or more of the exclusion criteria |
| 153 | Impact of COVID-19 pandemic on home range in a suburban city in the Osaka metropolitan area | 2021 | [10.3390/su13168974](https://doi.org/10.3390/su13168974) | Web of Science | MDPI | No | Meet one or more of the exclusion criteria |
| 154 | Mobile phone data reveals spatiotemporal recreational patterns in conservation areas during the COVID pandemic | 2023 | 10.1038/s41598-023-47326-y | Web of Science | Nature | No | Meet one or more of the exclusion criteria |
| 155 | Is travel behaviour an equity issue? Using GPS location data to assess the effects of income and supermarket availability on travel reduction during the COVID-19 pandemic | 2021 | [10.1080/12265934.2021.1952890](https://doi.org/10.1080/12265934.2021.1952890) | Scopus | Taylor and Francis Ltd | No | Meet one or more of the exclusion criteria |
| 156 | Health care visits during the COVID-19 pandemic: A spatial and temporal analysis of mobile device data | 2021 | [10.1016/j.healthplace.2021.102679](https://doi.org/10.1016/j.healthplace.2021.102679) | PubMed | Elsevier Ltd | No | Meet one or more of the exclusion criteria |
| 157 | The corona crisis, data protection and tracking apps in the EU: the Czech and Austrian COVID-19 mobile phone apps in the battle against the virus | 2021 | [10.32422/mv-cjir.1764](https://doi.org/10.32422/mv-cjir.1764) | Scopus | [Institute of International Relations Prague](https://www.scimagojr.com/journalsearch.php?q=Institute%20of%20International%20Relations%20Prague&tip=pub) | No | Meet one or more of the exclusion criteria |
| 158 | From computational indicators to law into technologies: the Internet of Things, data analytics and encoding in COVID-19 contact-tracing apps | 2021 | [10.1017/S174455232100032X](https://doi.org/10.1017/S174455232100032X) | Scopus | Cambridge University Press | No | Meet one or more of the exclusion criteria |
| 159 | COVID-19 Contact Tracing: Ghana’s Efforts in the Application of Geospatial Technology in Minimizing the Impact of the Pandemic | 2021 | 10.24018/ejdevelop.2021.1.4.47 | Scopus | [Palgrave Macmillan Ltd](https://www.scimagojr.com/journalsearch.php?q=Palgrave%20Macmillan%20Ltd.&tip=pub) | No | Meet one or more of the exclusion criteria |
| 160 | The practicality of mobile applications in healthcare administration and COVID-19 pandemic | 2021 | 10.33102/uij.vol33no1.300 | Scopus | Universiti Sains Islam Malaysia | No | Meet one or more of the exclusion criteria |
| 161 | Mining user reviews of COVID contact-tracing apps: An exploratory analysis of nine European apps | 2022 | [10.1016/j.jss.2021.111136](https://doi.org/10.1016/j.jss.2021.111136) | PubMed | Elsevier Ltd | No | Meet one or more of the exclusion criteria |
| 162 | Toward a secure global contact tracing app for COVID-19 | 2022 | [10.1145/3530019.353133](https://doi.org/10.1145/3530019.3531339) | Scopus | ACM | No | Meet one or more of the exclusion criteria |
| 163 | Privacy-preserving COVID-19 contact tracing solution based on blockchain | 2023 | [10.1016/j.csi.2022.103643](https://doi.org/10.1016/j.csi.2022.103643) | PubMed | Elsevier Ltd | No | Meet one or more of the exclusion criteria |
| 164 | **COVID-19 Contact Tracing Using Blockchain** | 2021 | [10.1109/ACCESS.2021.3074753](https://doi.org/10.1109/ACCESS.2021.3074753) | PubMed | IEEE | No | Meet one or more of the exclusion criteria |
| 165 | Contact tracing apps for the COVID-19 pandemic: a systematic literature review of challenges and future directions for neo-liberal societies | 2020 | 10.1007/s13755-021-00147-7 | Web of Science | Springer | No | Meet one or more of the exclusion criteria |
| 166 | Effectiveness modelling of digital contact-tracing solutions for tackling the COVID-19 pandemic | 2021 | [10.1017/S0373463321000175](http://dx.doi.org/10.1017/S0373463321000175) | Scopus | Cambridge University Press | No | Meet one or more of the exclusion criteria |
| 167 | Estimates of the Carbon Impacts of Commute Travel Restrictions due to COVID-19 in the UK | 2021 | [10.32866/001C.21574](https://doi.org/10.32866/001C.21574) | Scopus | Finings | No | Meet one or more of the exclusion criteria |
| 168 | Modeling the effects of contact-tracing apps on the spread of the coronavirus disease: Mechanisms, conditions, and efficiency | 2021 | [10.1371/journal.pone.0256151](https://doi.org/10.1371/journal.pone.0256151) | Web of Science | Public Library of Science | No | Meet one or more of the exclusion criteria |
| 169 | Effectiveness evaluation of digital contact tracing for COVID-19 in New South Wales, Australia | 2022 | 1[0.1016/S2468-2667(22)00010-X](https://doi.org/10.1016/S2468-2667(22)00010-X) | PubMed | Elsevier Ltd | No | Meet one or more of the exclusion criteria |
| 170 | Contact tracing apps for self-quarantine in South Korea: rethinking datafication and dataveillance in the COVID-19 age | 2021 | [10.1108/OIR-08-2020-0377](http://dx.doi.org/10.1108/OIR-08-2020-0377) | Web of Science | Emerald Group Publishing | No | Meet one or more of the exclusion criteria |
| 171 | Investigating the effective factors of using mHealth apps for monitoring COVID-19 symptoms and contact tracing: A survey among Iranian citizens | 2021 | [10.1016/j.ijmedinf.2021.104571](https://doi.org/10.1016/j.ijmedinf.2021.104571) | PubMed | Elsevier Ltd | No | Meet one or more of the exclusion criteria |
| 172 | A framework for monitoring movements of pandemic disease patients based on GPS trajectory datasets | 2022 | 10.1007/s11276-021-02819-4 | PubMed | Springer | No | Meet one or more of the exclusion criteria |
| 173 | Using GPS-enabled mobile phones to evaluate the associations between human mobility changes and the onset of influenza illness | 2022 | [10.1016/j.sste.2021.100458](https://doi.org/10.1016/j.sste.2021.100458) | PubMed | Elsevier Ltd | No | Meet one or more of the exclusion criteria |
| 174 | Community Mobility and COVID-19 Dynamics in Jakarta, Indonesia | 2022 | [**10.3390/ijerph19116671**](https://doi.org/10.3390/ijerph19116671) | PubMed | MDPI | No | Meet one or more of the exclusion criteria |
| 175 | Exploring the Utility of Google Mobility Data During the COVID-19 Pandemic in India: Digital Epidemiological Analysis | 2021 | [10.2196/29957](https://doi.org/10.2196/29957) | PubMed | JMIR Publications Inc | No | Meet one or more of the exclusion criteria |
| 176 | **Cell Phone Activity in Categories of Places and Associations With Growth in Cases of COVID-19 in the US** | 2020 | 10.1001/jamainternmed.2020.4288 | PubMed | American Medical Association | No | Meet one or more of the exclusion criteria |
| 177 | COVID-19 is spatial: Ensuring that mobile Big Data is used for social good | 2020 | [10.1177/2053951720952088](https://doi.org/10.1177/2053951720952088) | PubMed | SAGE | No | Meet one or more of the exclusion criteria |
| 178 | Twitter reveals human mobility dynamics during the COVID-19 pandemic | 2020 | [10.1371/journal.pone.0241957](https://doi.org/10.1371/journal.pone.0241957) | PubMed | Public Library of Science | No | Meet one or more of the exclusion criteria |
| 179 | Using mobility data in the design of optimal lockdown strategies for the COVID-19 pandemic | 2021 | [10.1371/journal.pcbi.1009236](https://doi.org/10.1371/journal.pcbi.1009236) | PubMed | Public Library of Science | No | Meet one or more of the exclusion criteria |
| 180 | Comparative analysis of geolocation information through mobile-devices under different COVID-19 mobility restriction patterns in Spain | 2021 | [10.3390/ijgi10020073](https://doi.org/10.3390/ijgi10020073) | Web of Science | MDPI | No | Meet one or more of the exclusion criteria |
| 181 | Mobility and dissemination of COVID-19 in Portugal: correlations and estimates from Google’s mobility data | 2022 | [10.3390/data7080107](https://doi.org/10.3390/data7080107) | Web of Science | MDPI | No | Meet one or more of the exclusion criteria |
| 182 | Risk mapping for COVID-19 outbreaks in Australia using mobility data | 2021 | [10.1098/rsif.2020.0657](https://doi.org/10.1098/rsif.2020.0657) | Web of Science | Royal Society Publishing | No | Meet one or more of the exclusion criteria |
| 183 | Urban exodus? Understanding human mobility in Britain during the COVID‐19 pandemic using Meta‐Facebook data | 2022 | [10.1002/psp.2637](https://doi.org/10.1002/psp.2637) | Web of Science | Wiley | No | Meet one or more of the exclusion criteria |
| 184 | Cross-border mobility responses to COVID-19 in Europe: new evidence from Facebook data | 2022 | 10.1186/s12992-022-00832-6 | Web of Science | Nature | No | Meet one or more of the exclusion criteria |
| 185 | Understanding components of mobility during the COVID-19 pandemic | 2021 | [10.1098/rsta.2021.0118](https://doi.org/10.1098/rsta.2021.0118) | Web of Science | Royal Society Publishing | No | Meet one or more of the exclusion criteria |
| 186 | Mobile apps to fight the COVID-19 crisis | 2021 | [10.3390/data6100106](https://doi.org/10.3390/data6100106) | Scopus | MDPI | No | Meet one or more of the exclusion criteria |
| 187 | Smartphone apps in the COVID-19 pandemic | 2022 | 10.1038/s41587-022-01350-x | Web of Science | Nature | No | Meet one or more of the exclusion criteria |
| 188 | Visualizing social and behavior change due to the outbreak of COVID-19 using mobile phone location data | 2021 | 10.1007/s00354-021-00139-x | PubMed | Springer | No | Duplicate study |
| 189 | Hotspot analysis of COVID-19 infection using mobile-phone location data | 2022 | 10.1007/s10015-022-00830-2 | Web of Science | Springer | No | Duplicate study |
| 190 | A mobile health application using Geolocation for behavioral activity tracking | 2023 | [10.3390/s23187917](https://doi.org/10.3390/s23187917) | Web of Science | MDPI | No | Duplicate study |
| 191 | A city cluster risk-based approach for Sars-CoV-2 and isolation barriers based on anonymized mobile phone users’ location data | 2021 | [10.1016/j.scs.2020.102574](https://doi.org/10.1016/j.scs.2020.102574) | Web of Science | Elsevier Ltd | No | Duplicate study |
| 192 | Location-aware systems or location-based services: a survey with applications to CoViD-19 contact tracking | 2020 | 10.1007/s40860-020-00111-4 | Web of Science | Springer | No | Duplicate study |
| 193 | Evaluating Apple Inc mobility trend data related to the COVID-19 outbreak in Japan: Statistical analysis | 2020 | [10.2196/20335](https://doi.org/10.2196/20335) | Web of Science | JMIR Publications Inc | No | Duplicate study |
| 194 | Mining Google and Apple mobility data: Temporal anatomy for COVID-19 social distancing | 2021 | 10.1038/s41598-021-83441-4 | Web of Science | Nature | No | Duplicate study |
| 195 | Hotspot analysis of COVID-19 infection using mobile-phone location data | 2022 | 10.1007/s10015-022-00830-2 | Scopus | Springer | No | Duplicate study |
| 196 | A mobile health application using Geolocation for behavioral activity tracking | 2023 | [10.3390/s23187917](https://doi.org/10.3390/s23187917) | Scopus | MDPI | No | Duplicate study |
| 197 | A city cluster risk-based approach for Sars-CoV-2 and isolation barriers based on anonymized mobile phone users’ location data | 2021 | [10.1016/j.scs.2020.102574](https://doi.org/10.1016/j.scs.2020.102574) | Scopus | Elsevier Ltd | No | Duplicate study |
| 198 | Location-aware systems or location-based services: a survey with applications to CoViD-19 contact tracking | 2020 | 10.1007/s40860-020-00111-4 | Scopus | Springer | No | Duplicate study |
| 199 | Evaluating Apple Inc mobility trend data related to the COVID-19 outbreak in Japan: Statistical analysis | 2020 | [10.2196/20335](https://doi.org/10.2196/20335) | Scopus | JMIR Publications Inc | No | Duplicate study |
| 200 | Using passive Wi-Fi for community crowd sensing during the COVID-19 pandemic. | 2023 | 10.1186/s40537-022-00675-3 | Scopus | Springer | No | Duplicate study |
| 201 | Application of semantic location awareness computing based on data mining in COVID-19 prevention and control system | 2020 | 10.3233/JIFS-189295 | Scopus | IOS Press | No | Duplicate study |
| 202 | Location-aware systems or location-based services: a survey with applications to CoViD-19 contact tracking | 2020 | 10.1007/s40860-020-00111-4 | Scopus | Springer | No | Duplicate study |
| 203 | Evaluating Apple Inc mobility trend data related to the COVID-19 outbreak in Japan: Statistical analysis | 2020 | [10.2196/20335](https://doi.org/10.2196/20335) | Scopus | JMIR Publications Inc | No | Duplicate study |
| 204 | Mining Google and Apple mobility data: Temporal anatomy for COVID-19 social distancing | 2021 | 10.1038/s41598-021-83441-4 | Scopus | Nature | No | Duplicate study |
| 205 | Assessing the Governance of Digital Contact Tracing in Response to COVID-19: Results of a Multi-National Study | 2022 | [10.1017/jme.2023.20](https://doi.org/10.1017/jme.2023.20) | Scopus | Cambridge University Press | No | Duplicate study |
| 206 | Using passive Wi-Fi for community crowd sensing during the COVID-19 pandemic. | 2023 | 10.1186/s40537-022-00675-3 | Web of Science | Springer | No | Duplicate study |
| 207 | Application of semantic location awareness computing based on data mining in COVID-19 prevention and control system | 2020 | 10.3233/JIFS-189295 | Web of Science | IOS Press | No | Duplicate study |
| 208 | Location-aware systems or location-based services: a survey with applications to CoViD-19 contact tracking | 2020 | 10.1007/s40860-020-00111-4 | Web of Science | Springer | No | Duplicate study |
| 209 | Evaluating Apple Inc mobility trend data related to the COVID-19 outbreak in Japan: Statistical analysis | 2020 | [10.2196/20335](https://doi.org/10.2196/20335) | Web of Science | JMIR Publications Inc | No | Duplicate study |
| 210 | Mining Google and Apple mobility data: Temporal anatomy for COVID-19 social distancing | 2021 | 10.1038/s41598-021-83441-4 | Web of Science | Nature | No | Duplicate study |
| 211 | Assessing the Governance of Digital Contact Tracing in Response to COVID-19: Results of a Multi-National Study | 2022 | [10.1017/jme.2023.20](https://doi.org/10.1017/jme.2023.20) | Web of Science | Cambridge University Press | No | Duplicate study |
| 212 | Investigating the effective factors of using mHealth apps for monitoring COVID-19 symptoms and contact tracing: A survey among Iranian citizens | 2021 | [10.1016/j.ijmedinf.2021.104571](https://doi.org/10.1016/j.ijmedinf.2021.104571) | Scopus | Elsevier Ltd | No | Duplicate study |
| 213 | A framework for monitoring movements of pandemic disease patients based on GPS trajectory datasets | 2022 | 10.1007/s11276-021-02819-4 | Scopus | Springer | No | Duplicate study |
| 214 | Using GPS-enabled mobile phones to evaluate the associations between human mobility changes and the onset of influenza illness | 2022 | [10.1016/j.sste.2021.100458](https://doi.org/10.1016/j.sste.2021.100458) | Scopus | Elsevier Ltd | No | Duplicate study |
| 215 | Community Mobility and COVID-19 Dynamics in Jakarta, Indonesia | 2022 | [**10.3390/ijerph19116671**](https://doi.org/10.3390/ijerph19116671) | Scopus | MDPI | No | Duplicate study |
| 216 | Exploring the Utility of Google Mobility Data During the COVID-19 Pandemic in India: Digital Epidemiological Analysis | 2021 | [10.2196/29957](https://doi.org/10.2196/29957) | Scopus | JMIR Publications Inc | No | Duplicate study |
| 217 | **Cell Phone Activity in Categories of Places and Associations With Growth in Cases of COVID-19 in the US** | 2020 | 10.1001/jamainternmed.2020.4288 | Scopus | American Medical Association | No | Duplicate study |
| 218 | COVID-19 is spatial: Ensuring that mobile Big Data is used for social good | 2020 | [10.1177/2053951720952088](https://doi.org/10.1177/2053951720952088) | Scopus | SAGE | No | Duplicate study |
| 219 | Twitter reveals human mobility dynamics during the COVID-19 pandemic | 2020 | [10.1371/journal.pone.0241957](https://doi.org/10.1371/journal.pone.0241957) | Scopus | Public Library of Science | No | Duplicate study |
| 220 | Using mobility data in the design of optimal lockdown strategies for the COVID-19 pandemic | 2021 | [10.1371/journal.pcbi.1009236](https://doi.org/10.1371/journal.pcbi.1009236) | Scopus | Public Library of Science | No | Duplicate study |
| 221 | Investigating the effective factors of using mHealth apps for monitoring COVID-19 symptoms and contact tracing: A survey among Iranian citizens | 2021 | [10.1016/j.ijmedinf.2021.104571](https://doi.org/10.1016/j.ijmedinf.2021.104571) | Web of Science | Elsevier Ltd | No | Duplicate study |
| 222 | A framework for monitoring movements of pandemic disease patients based on GPS trajectory datasets | 2022 | 10.1007/s11276-021-02819-4 | Web of Science | Springer | No | Duplicate study |
| 223 | Using GPS-enabled mobile phones to evaluate the associations between human mobility changes and the onset of influenza illness | 2022 | [10.1016/j.sste.2021.100458](https://doi.org/10.1016/j.sste.2021.100458) | Web of Science | Elsevier Ltd | No | Duplicate study |
| 224 | Community Mobility and COVID-19 Dynamics in Jakarta, Indonesia | 2022 | [10.3390/ijerph19116671](https://doi.org/10.3390/ijerph19116671) | Web of Science | MDPI | No | Duplicate study |
| 225 | Exploring the Utility of Google Mobility Data During the COVID-19 Pandemic in India: Digital Epidemiological Analysis | 2021 | [10.2196/29957](https://doi.org/10.2196/29957) | Web of Science | JMIR Publications Inc | No | Duplicate study |
| 226 | Cell Phone Activity in Categories of Places and Associations With Growth in Cases of COVID-19 in the US | 2020 | 10.1001/jamainternmed.2020.4288 | Web of Science | American Medical Association | No | Duplicate study |
| 227 | COVID-19 is spatial: Ensuring that mobile Big Data is used for social good | 2020 | [10.1177/2053951720952088](https://doi.org/10.1177/2053951720952088) | Web of Science | SAGE | No | Duplicate study |
| 228 | Twitter reveals human mobility dynamics during the COVID-19 pandemic | 2020 | [10.1371/journal.pone.0241957](https://doi.org/10.1371/journal.pone.0241957) | Web of Science | Public Library of Science | No | Duplicate study |
| 229 | Using mobility data in the design of optimal lockdown strategies for the COVID-19 pandemic | 2021 | [10.1371/journal.pcbi.1009236](https://doi.org/10.1371/journal.pcbi.1009236) | Web of Science | Public Library of Science | No | Duplicate study |
| 230 | Comparative analysis of geolocation information through mobile-devices under different COVID-19 mobility restriction patterns in Spain | 2021 | [10.3390/ijgi10020073](https://doi.org/10.3390/ijgi10020073) | Scopus | MDPI | No | Duplicate study |
| 231 | Mobility and dissemination of COVID-19 in Portugal: correlations and estimates from Google’s mobility data | 2022 | [10.3390/data7080107](https://doi.org/10.3390/data7080107) | Scopus | MDPI | No | Duplicate study |
| 232 | Risk mapping for COVID-19 outbreaks in Australia using mobility data | 2021 | [10.1098/rsif.2020.0657](https://doi.org/10.1098/rsif.2020.0657) | Scopus | Royal Society Publishing | No | Duplicate study |
| 233 | Urban exodus? Understanding human mobility in Britain during the COVID‐19 pandemic using Meta‐Facebook data | 2022 | [10.1002/psp.2637](https://doi.org/10.1002/psp.2637) | Scopus | Wiley | No | Duplicate study |
| 233 | Cross-border mobility responses to COVID-19 in Europe: new evidence from Facebook data | 2022 | 10.1186/s12992-022-00832-6 | Scopus | Nature | No | Duplicate study |
| 234 | Understanding components of mobility during the COVID-19 pandemic | 2021 | [10.1098/rsta.2021.0118](https://doi.org/10.1098/rsta.2021.0118) | Scopus | Royal Society Publishing | No | Duplicate study |
| 235 | Comparative analysis of geolocation information through mobile-devices under different COVID-19 mobility restriction patterns in Spain | 2021 | [10.3390/ijgi10020073](https://doi.org/10.3390/ijgi10020073) | PubMed | MDPI | No | Duplicate study |
| 236 | Mobility and dissemination of COVID-19 in Portugal: correlations and estimates from Google’s mobility data | 2022 | [10.3390/data7080107](https://doi.org/10.3390/data7080107) | PubMed | MDPI | No | Duplicate study |
| 237 | Risk mapping for COVID-19 outbreaks in Australia using mobility data | 2021 | [10.1098/rsif.2020.0657](https://doi.org/10.1098/rsif.2020.0657) | PubMed | Royal Society Publishing | No | Duplicate study |
| 238 | Urban exodus? Understanding human mobility in Britain during the COVID‐19 pandemic using Meta‐Facebook data | 2022 | [10.1002/psp.2637](https://doi.org/10.1002/psp.2637) | PubMed | Wiley | No | Duplicate study |
| 239 | Cross-border mobility responses to COVID-19 in Europe: new evidence from Facebook data | 2022 | 10.1186/s12992-022-00832-6 | PubMed | Nature | No | Duplicate study |
| 240 | Understanding components of mobility during the COVID-19 pandemic | 2021 | [10.1098/rsta.2021.0118](https://doi.org/10.1098/rsta.2021.0118) | PubMed | Royal Society Publishing | No | Duplicate study |
| 241 | COVID-19 Mobile Positioning Data Contact Tracing and Patient Privacy Regulations: Exploratory Search of Global Response Strategies and the Use of Digital Tools in Nigeria | 2020 | 10.2196/19139 | Web of Science | JMIR Publications Inc | No | Duplicate study |
| 242 | Challenges and opportunities in accessing mobile phone data for COVID-19 response in developing countries | 2021 | 10.1017/dap.2021.10 | Web of Science | Cambridge University Press | No | Duplicate study |
| 243 | Public health research using cell phone derived mobility data in sub-Saharan Africa: Ethical issues | 2023 | 10.17159/sajs.2023/14777 | Web of Science | African journals | No | Duplicate study |
| 244 | Analysis of call detail records to inform the COVID-19 response in Ghana—opportunities and challenges | 2021 | [10.1017/dap.2021.5](https://doi.org/10.1017/dap.2021.5) | Web of Science | Cambridge University Press | No | Duplicate study |
| 245 | Data sharing and collaborations with Telco data during the COVID-19 pandemic: A Vodafone case study | 2021 | [10.1017/dap.2021.26](https://doi.org/10.1017/dap.2021.26) | Web of Science | Cambridge University Press | No | Duplicate study |
| 246 | The ethical dilemma of mobile phone data monitoring during COVID-19: The case for South Korea and the United States | 2022 | 10.1177/22799036221102491 | Web of Science | SAGE | No | Duplicate study |
| 247 | Measuring mobility to monitor travel and physical distancing interventions: a common framework for mobile phone data analysis | 2020 | 10.1016/S2589-7500(20)30193-X | Web of Science | Elsevier Ltd | No | Duplicate study |
| 248 | The ethical dilemma of mobile phone data monitoring during COVID-19: The case for South Korea and the United States | 2022 | 10.1177/22799036221102491 | PubMed | SAGE | No | Duplicate study |
| 249 | Measuring mobility to monitor travel and physical distancing interventions: a common framework for mobile phone data analysis | 2020 | 10.1016/S2589-7500(20)30193-X | PubMed | Elsevier Ltd | No | Duplicate study |
| 250 | Public health research using cell phone derived mobility data in sub-Saharan Africa: Ethical issues | 2023 | 10.17159/sajs.2023/14777 | PubMed | African journals | No | Duplicate study |
| 251 | A robust tracking system for COVID-19 like pandemic using advanced hybrid technologies | 2023 | 10.1007/s00607-021-00946-6 | Web of Science | IEEE | No | Duplicate study |
| 252 | Using crowd-sourced data for real-time monitoring of food prices during the COVID-19 pandemic: Insights from a pilot project in northern Nigeria | 2021 | 10.1016/j.gfs.2021.100523 | Web of Science | Elsevier Ltd | No | Duplicate study |
| 253 | Scale-free dynamics of COVID-19 in a Brazilian city | 2023 | 10.1016/j.apm.2023.03.039 | Web of Science | Elsevier Ltd | No | Excluded during title and abstract screening for irrelevance |
| 254 | Investigating the Use of Digital Health Technology to Monitor COVID-19 and Its Effects: Protocol for an Observational Study (Covid Collab Study) | 2021 | 10.2196/32587 | Web of Science | JMIR Publications Inc. | No | Excluded during title and abstract screening for irrelevance |
| 255 | Segregation and the pandemic: The dynamics of daytime social diversity during COVID-19 in Greater Stockholm | 2023 | 10.1016/j.apgeog.2023.102926 | Web of Science | Elsevier Ltd | No | Excluded during title and abstract screening for irrelevance |
| 257 | Community Mobility and COVID-19 Dynamics in Jakarta, Indonesia | 2022 | 10.3390/ijerph19116671 | Web of Science | MDPI | No | Excluded during title and abstract screening for irrelevance |
| 258 | Forecasting hospital-level COVID-19 admissions using real-time mobility data | 2023 | 10.1038/s43856-023-00253-5 | Web of Science | Nature | No | Excluded during title and abstract screening for irrelevance |
| 259 | The impact of the Covid-19 pandemic and government intervention on active mobility | 2022 | 10.1016/j.tra.2022.09.007 | Web of Science | Elsevier Ltd | No | Excluded during title and abstract screening for irrelevance |
| 260 | Thinning out spectators: Did football matches contribute to the second COVID-19 wave in Germany? | 2022 | 10.1515/ger-2021-0060 | Web of Science | De Gruyter Open Ltd | No | Excluded during title and abstract screening for irrelevance |
| 261 | Who were the losers and winners during the Covid-19 pandemic? The rise of remote working in suburban areas | 2022 | 10.1080/21681376.2022.2139194 | Web of Science | Routledge | No | Excluded during title and abstract screening for irrelevance |
| 262 | Access to Maternal Health Services During the COVID-19 Pandemic: Experiences of Indigent Mothers and Health Care Providers in Kilifi County, Kenya | 2021 | 10.3389/fsoc.2021.613042 | Web of Science | Frontiers | No | Excluded during title and abstract screening for irrelevance |
| 263 | Do the lockdown-imposed changes in a wastewater treatment plant catchment's socio-demographics impact longitudinal temporal trends in psychoactive pharmaceutical use? | 2023 | 10.1016/j.scitotenv.2023.162342 | Web of Science | Elsevier Ltd | No | Excluded during title and abstract screening for irrelevance |
| 264 | Visits in Forests During the Covid-19 Pandemic in the Cross-Border Area of Poland, the Czech Republic and Germany | 2023 | 10.14746/quageo-2023-0016 | Web of Science | Sciendo | No | Excluded during title and abstract screening for irrelevance |
| 265 | Structural changes in intercity mobility networks of China during the COVID-19 outbreak: A weighted stochastic block modeling analysis | 2022 | [10.1016/j.compenvurbsys.2022.101846](https://doi.org/10.1016/j.compenvurbsys.2022.101846) | Web of Science | Elsevier Ltd | No | Duplicate study |
| 266 | Using mobile phone data for epidemic response in low resource settings—A case study of COVID-19 in Malawi | 2021 | 10.1017/dap.2021.14 | Web of Science | Cambridge University Press | No | Duplicate study |
| 267 | A Privacy-Preserved and Cost-Efficient Control Scheme for Coronavirus Outbreak Using Call Data Record and Contact Tracing | 2021 | 10.1109/MCE.2020.3038023 | Web of Science | IEEE | No | Duplicate study |
